# Bayesian inference of origin firing time distributions, origin interference and licencing probabilities from Next Generation Sequencing data

**DOI:** 10.1093/nar/gkz094

**Published:** 2019-02-14

**Authors:** Alina Bazarova, Conrad A Nieduszynski, Ildem Akerman, Nigel J Burroughs

**Affiliations:** 1 Centre for Computational Biology, Institute of Cancer and Genomic Sciences, University of Birmingham, Birmingham B15 2TT, UK; 2 Sir William Dunn School of Pathology, Oxford University, Oxford OX1 3RE, UK; 3 Institute of Metabolism and Systems Research, Institute of Biomedical Research, University of Birmingham, Birmingham B15 2TT, UK; 4 Mathematics Institute and Zeeman Institute (SBIDER), University of Warwick, Coventry CV4 7AL, UK

## Abstract

DNA replication is a stochastic process with replication forks emanating from multiple replication origins. The origins must be licenced in G1, and the replisome activated at licenced origins in order to generate bi-directional replication forks in S-phase. Differential firing times lead to origin interference, where a replication fork from an origin can replicate through and inactivate neighbouring origins (origin obscuring). We developed a Bayesian algorithm to characterize origin firing statistics from Okazaki fragment (OF) sequencing data. Our algorithm infers the distributions of firing times and the licencing probabilities for three consecutive origins. We demonstrate that our algorithm can distinguish partial origin licencing and origin obscuring in OF sequencing data from *Saccharomyces cerevisiae* and human cell types. We used our method to analyse the decreased origin efficiency under loss of Rat1 activity in *S. cerevisiae*, demonstrating that both reduced licencing and increased obscuring contribute. Moreover, we show that robust analysis is possible using only local data (across three neighbouring origins), and analysis of the whole chromosome is not required. Our algorithm utilizes an approximate likelihood and a reversible jump sampling technique, a methodology that can be extended to analysis of other mechanistic processes measurable through Next Generation Sequencing data.

## INTRODUCTION

In eukaryotes, replication of DNA is achieved by establishment of multiple bi-directional replication forks at genomic sites called replication origins (1,2). In order to ensure that the genome is replicated once and only once per cell cycle, a two-step process takes place. First, the pre-replicative complex (Pre-RC), which contains the origin recognition complex and minichromosome maintenance (MCM) helicases is loaded onto origins during G1 phase. This is referred to as origin licencing and is temporally restricted to the G1 phase. During S-phase, when Pre-RC formation is no longer permitted, the Pre-RCs are activated through the action of cyclin-dependent kinases. It is estimated that many origins are licenced during each G1 phase, and only a fraction (approximately one-fourth) of these licenced origins are activated in S-phase (3,4).

The DNA replication machinery is relatively well understood in *Saccharomyces cerevisiae* and has been reconstituted *in vitro*, (5) where replication kinetics were similar to those of *in vivo* replication rates (6). Despite our understanding of the DNA replication machinery, our understanding of its regulation and kinetic control *in vivo* is sparse. Replication origin activation (firing) is a highly regulated but stochastic process. Replication occurs in replication domains with similar replication timing, giving rise to origin clustering (7,8). A number of factors have been reported to control firing time; in budding yeast this includes (2) chromosome location, in particular proximity to centromeres (early) and telomeres (late), local chromatin organization, the number of loaded MCMs during licencing (9) and proximal recruitment of activating or inhibitory factors, e.g. (10,11). Following activation, replication forks are proposed to move away from the origin at on average constant speeds (12). In particular, it has been proposed that forks emanating from neighbouring origins have similar speeds (13,14). DNA synthesis of a strand ends when the fork collides with an incoming fork from an adjacent fired origin, which is largely a passive phenomenon (15,16).

The time to achieve complete DNA duplication is a complex function of the licenced origins' firing times within a replication cycle. With the emergence of powerful sequencing technologies, it is reasonable to expect that this stochastic process can be parametrized from experimental data, thereby achieving a new level of understanding. This is the question we tackle here: can the stochastic origin replication process incorporating probabilistic origin licencing and variability in origin firing times be inferred from sequencing data? We develop a Bayesian approach to fit the model of Retkute *et al.* (17,18), generating a full parametrization of origin use and firing times from Okazaki fragment (OF) sequencing data. This model has been well tested against a variety of data types (16) and accounts for both firing time variability and differential origin activation. The latter effectively subsumes origin licencing and the probability that a licenced origin matures to an active replisome in absence of passive replication by its neighbours. We follow the terminology of (17) and simply refer to this as the licencing probability.

In this study, we present a computational Bayesian algorithm to fit a mechanistic stochastic replication model to sequencing data. Applying our method to budding yeast OF sequencing data, we present examples of origins with different levels of licencing and obscuring (passive replication) from neighbouring origins, and we analyse the whole of chromosome 10. We demonstrate how, even with noisy sequencing pile-up profiles, important biological insight can be achieved. Namely we recover origin firing times and licencing probabilities along with their distributions, therefore allowing us to quantify origin interference in budding yeast. We also explore origin characteristics of the *S. cerevisiae rat1-1* mutant. Rat1 is a ribo-exonuclease which participates in the transcription termination, namely in a process known as the torpedo process (19). We show that our method is able to detect the decreased origin efficiency in this mutant compared to the WT and decompose that efficacy loss in terms of reduced licencing and increased obscuring, with the stronger effect exhibited by the latter. Finally, we also demonstrate our algorithm on human data to identify origin replication parameters.

## MATERIALS AND METHODS

### Model and inference algorithm

Replication of a single genome will generate OFs from one strand, giving an OF profile with constant OF density up to the replication fork, Figure 2A and B. An OF sequencing experiment is however a population average, on the scale of *M* > 10^6^ cells, giving an averaged profile Figure 2C–F. The profile of Figure 2C corresponds to forks from fully licenced origins that terminate between neighbouring origins with negligible probability of terminating close to either neighbour. In this case there is no origin interference; between two origins the profile is only a function of their firing time distributions giving a simple tanh-like transition between the two origins. There are two factors that reduce the fork generation frequency. First, origins need to be licenced in order to fire. Secondly, as origin firing is delayed, the probability that a left-moving or right-moving fork from another origin reaching that origin before it fires increases. This is ‘obscuring’ from the right and left, respectively; see Supplementary Figures S1-3 for simulation examples. These two events essentially lead to the same outcome—an origin fails to generate replication forks. The jump, or step, in the profile at an origin in fact corresponds to the fraction of dividing cells where the origin produces replication forks, both of the above processes reducing this step size. Unravelling which event has occurred, and thus correctly estimating the obscuring probability and the licencing probability requires reconstruction of the firing distributions from the profile shape. These two processes, obscuring and partial licencing, can produce complex profiles as illustrated for the middle origin in Figure 2D–F and Supplementary Figures S2 and S3. We refer to an origin as strong if it has a jump of over 50%, i.e. the probability of being obscured is low and it has a high licencing probability. It is weak otherwise.

**Figure 1.**
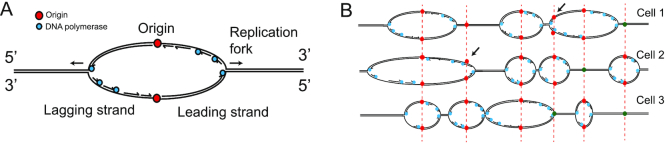
DNA replication schematic. (**A**) A fired origin showing bi-directional forks. Forward strand (3′ to 5′) is synthesized from the reverse strand (5′-3′, illustrated as top strand). The leading strand, with template 3′5′ is replicated as a continuous strand, whilst the reverse strand is replicated in discrete fragments called OF. Polymerases refer to Polε on the leading strand and Polα/Polδ on the lagging strand. (**B**) Schematic of DNA replication in three cells showing stochastic nature of replication, with different origins firing in individual cells, and origins firing at different times. Licenced origins shown in red, unlicenced in green. Origins replicated passively by obscuring from neighbours shown by arrows.

**Figure 2.**
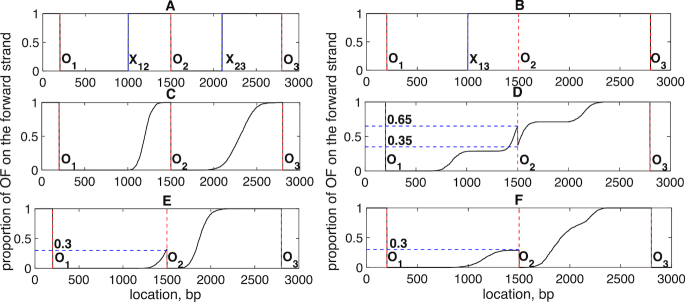
Simulated OF density profiles. (**A**) An example of a replication profile from a single cell with three origins at *O*_1_ (200 bp), *O*_2_ (1500 bp) and *O*_3_ (2800 bp). There are two replication fork collision points, their positions determined by the blue lines: *x*_12_ (1000 bp) (collision of *O*_1_ and *O*_2_ replication fork), *x*_23_ (2100 bp) (collision of *O*_2_ and *O*_3_ replication fork). (**B**) An example of a single replication profile with three origins (as A) where middle origin *O*_2_ overrun by the left-moving fork. *x*_13_ is the collision point of the *O*_1_, *O*_3_ replication forks. Vertical blue dashed lines are as A. (**C**) Simulated averaged profile for origins as A. Based on averaging 1000 single profiles with firing time differences *t*_2_ − *t*_1_ and *t*_3_ − *t*_2_ that are Normally distributed, *N*(700, 141^2^) (mean 700 bp, S.D. 141 bp) and *N*(300, 282^2^), respectively. (**D**) Simulated averaged profile with partial licencing of middle origin (equal firing time distributions). Middle origin *O*_2_ is not licenced in 70% of the cases. Firing time distributions are *t*_1_ ∼ *N*(100, 100^2^), *t*_2_ ∼ *N*(100, 100^2^), *t*_3_ ∼ *N*(100, 100^2^). (**E**) Simulated averaged profile with obscured middle origin. Middle origin *O*_2_ is obscured from the left in 70% of the cases. Firing time distributions are *t*_1_ ∼ *N*( − 602, 196^2^), *t*_2_ ∼ *N*(800, 100^2^), *t*_3_ ∼ *N*(100, 100^2^). (**F**) Simulated averaged profile with partially licenced middle origin. Middle origin *O*_2_ is not licenced in 70% of the cases. Firing time distributions are *t*_1_ ∼ *N*( − 602, 196^2^), *t*_2_ ∼ *N*(0, 100^2^), *t*_3_ ∼ *N*(100, 100^2^). Vertical red dashed lines indicate location of origins: *O*_1_ (200 bp), *O*_2_ (1500 bp) and *O*_3_ (2800 bp). Horizontal blue dashed lines correspond to licencing/obscuring levels. In panels D, E, F, *F*_ave_ constructed from 5000 duplications.

We utilize the model of (18). The two origin version of this model was analysed in (17), where it was shown that the origin firing time distribution could be estimated from the replication time profile, and later generalized to *N*-origins (18). This model has also previously been fitted to data (16); our methods extend this model fitting, giving full estimates of all the parameters and their confidence.

For our Bayesian inference algorithm, we model the system by constructing an (approximate) forward strand (3′ to 5′) profile as an average of *M* single cell profiles, $F^{{\rm ave}} = \frac{1}{M}\sum _k F^k$, where *F*^*k*^ is a single profile, such as Figure 2A, the average being a smoothed profile because of the variability in the firing times, e.g. Figure 2C (this profile is still piece-wise constant but the steps are now 1/*M* so the profile looks smooth for sufficiently large *M*). We will use an *M* in the thousands as an approximation to the OF experimental profile, this being computationally tractable and sufficiently accurate. Sequencing introduces measurement noise, noise that scales with the signal, Supplementary Data S1.8, and Supplementary Figures S4 and S5, a key hall-mark of log-Normal noise. We define the OF counts model for the forward strand (*f*, 3′-5′), counts $X_j^f$, and reverse strand (*r*, 5′-3′), counts $X_j^r$, at (boxed) genome position *j*,

(1) \begin{eqnarray*} &&X_j^f\sim ((1-b)F_j^{{\rm ave}}+0.5b)\exp \left(N(-0.5\tau ^{-1},\tau ^{-1})\right),\cr && X_j^r\sim ((1-b)(1-F_j^{{\rm ave}})+0.5b)\exp \left( N(-0.5\tau ^{-1},\tau ^{-1})\right),\cr && j\in \lbrace 1,\dots N_{{\rm boxes}}\rbrace . \end{eqnarray*}

Here, the parameter *b* ∈ [0, 1] determines the relative weighting of random DNA fragments generated, for example, during the extraction process, to the OF profile signal, τ is the measurement noise parameter and *N*_boxes_ is the number of boxed sites the genome is split into (we use boxing by 50 bp). Since replication on the reverse strand is complementary to the forward strand the replication profile on the reverse strand $F_j^{{\rm ave},r}$ satisfies $F_j^{{\rm ave},r} = 1-F_j^{{\rm ave}}$. Measurement noise is assumed log-Normal as suggested by the data, Supplementary Figures S4 and S5; *N*(−0.5τ^−1^, τ^−1^) is the Gaussian distribution with precision τ = variance^−1^ and mean −0.5τ^−1^. The mean is non-zero to impose the condition *E*[exp *N*(−0.5τ^−1^, τ^−1^)] = 1, which corresponds to the data normalization condition: the data are assumed normalized such that on average the summed normalized counts on both strands sum to 1, i.e. ${ E}[X^f_j+X^r_j] = 1$. The distances between the origins are *O*_1_*O*_2_ = *N*_1_, *O*_2_*O*_3_ = *N*_2_.

We use a Markov chain Monte Carlo (MCMC) algorithm to sample from the posterior probability of the parameters, i.e. the probability of the parameters conditioned on the experimental data (the posterior), Supplementary Data S1.3. Convergence was determined using a multiple chain protocol and the Gelman–Rubin statistic (20), Supplementary Data S1.5. On simulated data the true parameter values are accurately inferred, Supplementary Data S1.6, and e.g. Supplementary Figure S8. We use *M* = 4992 throughout, lower *M* gave discretization artefacts (Supplementary Figure S15). In the presented analysis we use a model that assumes the same noise τ and random fragmentation *b* (background noise) parameters on the two strands. More general models, Supplementary Data S1.9, with differing noise levels on the forward and reverse strands indicated that the forward strand is 30% noisier than the reverse, but has less contamination by random fragmentation, Supplementary Figure S6. However, allowing for different noise levels had negligible effect on the posterior distributions of the other parameters, Supplementary Figure S6C, so this generalization was not used any further.

In our origin firing model random fragmentation parametrized by *b*, mixes the OF profile with a uniform profile. However, certain firing configurations lead to uniform profiles in this triple origin model; if an end origin obscures the two others the profile in the whole region *O*_1_*O*_3_ is flat and equal to 0 or 1. Since this profile is indistinguishable from the background noise we prohibit these double overrun states in the inference algorithm. By estimating their frequency from the examples we analysed, they are in fact extremely rare and have negligible impact on the parameters in our examples.

### Data and data processing

Data from (21) were analysed using our algorithm, first, with sets of three or four consecutive origins to demonstrate various scenarios, and secondly, for 92% of chromosome 10, chosen since the OF profiles demonstrate the highest quality across the whole genome. Origin locations are taken from (22). Criteria for choosing the triples/quadruplets examples were that they showed good negative correlation between the two strands, and the end origins were strong, i.e. the region between two inner origins of a quadruplet is independent of forks coming from outside the analysed region.

We processed the data as follows: we aligned the raw paired-end sequencing data from (21) using bowtie2 (23), extracting only the reads with mapping quality >10. For each pair of reads we identified the fragment spanned by the reads and a section between them (if any). We discard fragments that are shorter than 120 bp and longer than 200 bp based on the distribution of the OF length. The remaining fragments were pooled to create coverage data for each strand. For the single end sequencing data of the *rat1-1* mutant (24) we aligned sequences with bowtie2 (23) and use the genome coverage tool (25) to create coverage data for each strand based on the reads alone. Pre-processed pile-up data for HeLa cells was obtained from (26).

We processed the pile-up data as follows. For each chromosome we computed the strand bias *b*_chr_ by summing up the read counts on the whole chromosome on each strand and taking their ratio

(2) \begin{equation*} b_{{\rm chr}} = \frac{\sum _{i = 1}^Nc_i^f}{\sum _{i = 1}^Nc_i^r}, \end{equation*}

where *N* is the length of the chromosome (in bps), and $c_i^f$ and $c_i^r$ are the read counts at position *i* ∈ {1, …, *N*} on the forward and reverse strands, respectively. We use *b*_chr_ to correct the counts for this measurement bias on the reverse strand. Each data set was boxed by *s*_box_ = 50 bp (to decrease count variance) and locally normalized by the average count *n*(*O*_1_, *O*_3_) between origins *O*_1_, *O*_3_, defining the normalized, boxed, forward strand count

(3) \begin{eqnarray*} &&c_i^{f^{\prime }} = \frac{1}{s_{{\rm box}}n(O_1,O_3)}\sum _{j = s_{{\rm box}}(i-1)+1}^{s_{{\rm box}}i}c_j^f ,\cr &&i \in \lbrace 1,\dots ,\lfloor (O_3-O_1)/s_{{\rm box}}\rfloor \rbrace \end{eqnarray*}

and similarly for $c_i^{r^{\prime }}$, but weighted by *b*_chr_. Here, $n(O_1,O_3) = \frac{1}{O_3-O_1}\sum\nolimits_{i = O_1}^{O_3} \left(c_i^f+b_{{\rm chr}}c_i^r\right)$, with *O*_1_, *O*_3_ the locations of the first and third origins of our triplet. When comparing inference across overlapping triplets we normalize over four origins, i.e. normalized by *n*(*O*_1_, *O*_4_). Hence, the normalized counts satisfy $\frac{1}{\lfloor (O_3-O_1)/s_{{\rm box}}\rfloor }\sum\nolimits _{i = 1}^{\lfloor (O_3-O_1)/s_{{\rm box}}\rfloor } \left(c_i^{f^{\prime }}+c_i^{r^{\prime }}\right) = 1$ analogous to the model’s normalization of *X*^*f*^, *X*^*r*^.

## RESULTS

### Analysing OF sequencing data using an MCMC algorithm

Here, we use a Bayesian MCMC algorithm to analyse the OF data from two protocols: (i) *S. cerevisiae* OF sequencing from ligase mutants, where OFs were harvested after 2.5 h of ligase inactivation. The DNA damage checkpoint was deactivated by deleting the *RAD9* gene to ensure that the S phase is completed and therefore would not affect the replication dynamics. Paired-end (WT) and single-end sequencing (*rat1-1* mutant) was used. (ii) Human OK-seq (HeLa) based on immuno-pull down and sequencing of OFs labelled with EdU. Both protocols isolate and sequence OFs, an intermediate formed during DNA replication. OF sequencing allows us to obtain the proportion of left- and right-moving forks across the genome.

In our model, following (17), we assume that the licencing probability and firing times are independent of each other (1,27). In contrast, the models of (28) as well as the ones of (29,30), which use the Kolmogorov-Johnson-Mehl-Avrami model framework (31), do not have an explicit process for differential origin use except through passive replication so the differential impact of origin firing times and origin selection cannot be analysed. Models have been fitted previously to individual replication profiles to infer origin firing characteristics. In particular, (17,18) demonstrated that firing time variability determined termination site width, directly linking the model firing time parameters to profile shape. This analysis was influential in demonstrating that stochastic firing of origins could reproduce differential origin timings, thus supporting the notion that replicon programmes with temporally regulated origin firing do not need to be present (32). The effect of chromatin conformation on origin firing was modelled in (33) using a non-local model of DNA replication. However, a full parametrization of a stochastic replication model has not been achieved from data to date.

Our analysis, based on three neighbouring origins, demonstrates that model parameter inference can be performed locally in many cases and a whole genome analysis is not necessary. We show that our results agree with previous analysis using different methods/data (using the same model see Supplementary Table S4, (16), using a different model see Supplementary Table S4, (15)).

A fork collision model comprises three origins (origin triplet), labelled consecutively *O*_1_, *O*_2_, *O*_3_, with distances *N*_1_, *N*_2_ between origins *O*_1_*O*_2_ and *O*_2_*O*_3_, respectively. We assume no forks come in from outside this triplet of origins. Origins are assumed to be licenced to fire with probabilities *q*_1_, *q*_2_, *q*_3_, and when licenced have potential firing times *t*_*i*_ that are Gaussian distributed; an incoming fork may arrive before firing (obscuring). Gaussian firing distributions were previously explored in the literature (see (18)) and we address this question in more detail in the Supplementary Data S1.7. Forks are assumed to move at the same speed and fork termination is passive through fork collision; this allows us to measure time in replicated base-pairs (rbps). In ‘Discussion’ section, we address the question of variable fork speed, specifically if the expected signature for speed variability is present in the data, Figure 12 and if our results are robust to speed variability, Supplementary Data S1.11.

In Supplementary Figure S17, we demonstrate that simulated data using the inferred parameters look similar to the experimental data which justifies the use of our model.

### Lack of origin obscuring in strong origin triplets: *ARS717-20*

**Figure 3.**
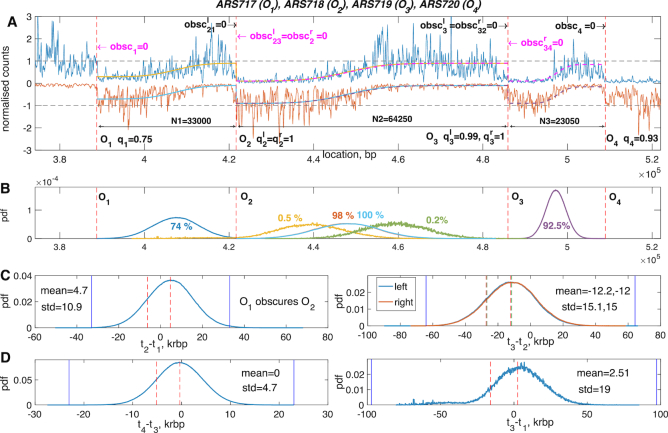
Four strong consecutive origins *ARS717-20*. Analysis of the region between *ARS717* and *ARS720* on chromosome 7 obtained by applying the algorithm separately on the left (*ARS717, ARS718, ARS719*) and right (*ARS718, ARS719, ARS720*) triplets. (**A**) Annotation of licencing and obscuring probabilities of origins shown with NGS data on the forward (blue) and reverse (red) strands and reconstructed fragment profiles (dashed magenta and purple for the right triplet, solid yellow and blue for the left one). Dashed vertical lines indicate the locations of the four origins *O*_1_, *O*_2_, *O*_3_, *O*_4_. Inferred licencing probability $q_i^{l/r}$ of origin *i*; inferred obscuring rate ${\rm obsc}_{ij}^{l/r}$ of the *i*th origin by *j*th one. In case of the end origin the only subscript is *i*. Superscripts *r* and *l* refer to whether the quantity was obtained by running the algorithm on the right or left triplet, respectively. Arrows next to obscuring rates indicate the direction of the replication fork coming from the neighbouring origin. Magenta text corresponds to forks replicating the forward strand in OF fragments, black text corresponds to forks replicating the forwards strand continuously. (**B**) Probability density distributions of the realized collisions between origins *O*_1_ and *O*_2_ (blue), *O*_2_ and *O*_3_ (red inferred from the left triple, light blue inferred from the right one), *O*_1_ and *O*_3_ (yellow), *O*_3_ and *O*_4_ (purple), *O*_2_ and *O*_4_ (green). Percentages correspond to the amount of time fork collision was realized (text colour corresponds to the colour of the distribution). (**C**) and (**D**) Inferred distributions of the firing time differences between neighbouring origins conditioned on origin being licenced. *t*_2_ and *t*_1_ (C, left panel), *t*_3_ and *t*_2_ for the left (blue) and right (red) triplets (C, right panel), *t*_4_ and *t*_3_ (D, left panel), *t*_3_ and *t*_1_ (D, right panel). Time is given in terms of krbp. Vertical blue lines divide the plots into three parts—the middle part corresponds to the case of no obscuring with respect to those two origins, the left to obscuring of the left origin by the right one, and the right obscuring of the right origin by the left one. The mean and S.D. of the firing time difference are given. Dashed vertical lines indicate the mean and 1 S.D. to its left for the firing time difference distributions. In case of *t*_3_−*t*_2_ the first value for the mean and S.D. correspond to inference from the left triplet (red dashed lines) and the second one corresponds to the right one (green dashed lines). Only MCMC samples for the firing time difference when both origins were licenced are used. Inference based on a single MCMC run with burn-in 100 000, and 100 000 samples post burn-in. See Supplementary data for MCMC information (Supplementary Data S1.1), and Table 1 for posterior parameters.

Here, we provide an example of a region with strong origins exhibiting minimal obscuring as quantified by our algorithm. Namely, we analyse the two overlapping origin triplets *ARS717-19* and *ARS718-20* on chromosome 7 allowing comparison of inferred model parameters on their overlap, labelling the origins *O*_1_, *O*_2_, *O*_3_, *O*_4_. The OF sequencing data in this region show clear tanh-like OF profiles on both the forward and reverse strands, Figure 3, similar to the example in Figure 2C, suggesting that the replication forks meet between neighbouring origins for all three consecutive origin pairs.

**Figure 4.**
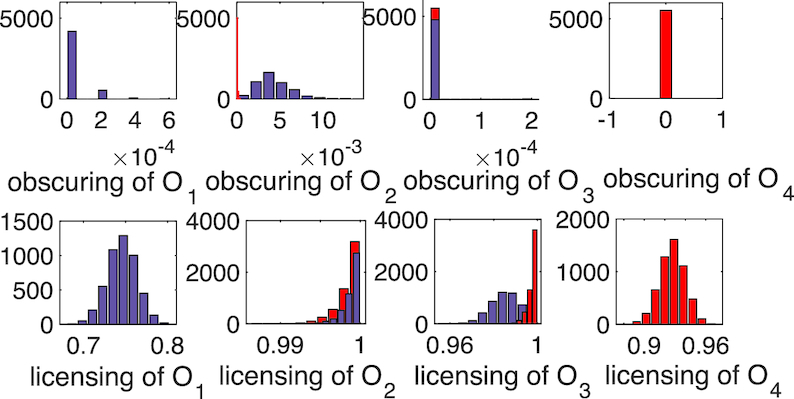
*ARS717-720* obscuring (upper panel) and licencing (lower panel) probabilities. Posterior probability distributions for obscuring and licencing in the population. Red are the histograms inferred from the right triplet, blue are inferred from the left triplet.

By fitting the fork replication model the mean OF density of the sampled population can be reconstructed, effectively removing measurement noise, Figure 3A, and the fork collision point density distribution can also be inferred, Figure 3B. Stochasticity in origin licencing is reflected in the distributions of Figure 4, and the differential firing times in Figure 3C and D. Obscuring can also decrease the probability that two origin forks meet; however obscuring amongst these origins is low, with 0.46% obscuring being the highest (median, upper/lower quartiles 0.30%, 0.68%), Figure 4 (upper panel). Quartiles are given for obscuring and licencing probabilities throughout since these distributions can be highly skewed. This low obscuring probability is in fact apparent from the shape of the fork collision distributions, which appear Gaussian—if significant obscuring was present, the collision point distributions would extend towards the obscured origin and result in fork termination distributions that are truncated Gaussians. As regards to partial licencing in the *ARS717*-*ARS720* region the two end regions have origins that meet only 74% (median, upper/lower quartiles 73%, 76%) and 92.5% (median, upper/lower quartiles 91.7%, 93.2%) of the time because of partial licencing, Figure 4. There are a small number of fork collisions between forks from *O*_2_ and *O*_4_ at 0.2% of the time (median, upper/lower quartiles 0.1%, 0.3%) because of partial licencing of *O*_3_, these collisions occurring between *O*_2_, *O*_3_ (analysis of triplet *O*_234_).

We inferred the relative firing times between the origins (when both origins are licenced), Figure 3C and D. The first immediate observation is that all these distributions are approximately centred around zero, i.e. these four origins thus all fire at similar times. A key factor in understanding the impact that the spread of the firing time difference distribution has on the profile is the distance between the origins. In this example the distance *O*_2_*O*_3_ is relatively large and *O*_3_*O*_4_ is small. This gives time thresholds when a fork from the neighbour reaches that origin (and obscures it), which gives a scale to the firing time distributions, Figure 3C and D (recall we measure time in terms of rbp since fork speed is constant). This confirms that obscuring is negligible amongst these four origins as the firing times are all sufficiently tight and firing times are not too disparate, Figure 3C and D. In all cases the mean firing time difference of neighbouring origins < 0.2 |*N*_*i* ± 1_ − *N*_*i*_| which is sufficient to give sharp profiles. What is surprising is that the closer origins *O*_3_, *O*_4_ have a significantly tighter distribution, i.e. their firing times are closer than between the more distant origins, Table 1. This raises a question of whether the firing time S.D. are correlated with inter-origin distances and whether any conclusions can be made about fork velocity variability based on this. We address this later in ‘Discussion’ section. We can also compute the probability that one origin fires before another, *t*_*i* + 1_ < *t*_*i*_, factoring out the licencing probability, Table 1. This shows that *O*_3_, *O*_4_ typically fire at the same time, π(*t*_4_ < *t*_3_) = 0.47, *O*_2_ fires 67% earlier than *O*_1_, and *O*_2_ fires 21% earlier than *O*_3_. Hence, when *O*_3_ is not licenced, the fork from *O*_4_ traverses the small distance *N*_4_−*N*_3_ sufficiently quickly that the *O*_2_*O*_4_ forks collide to the left of *O*_3_ as observed in Figure 3B. These conclusions are consistent with the time series data of (16), which indicate that *O*_3_ (*ARS719*) and *O*_4_ (*ARS720*) are strong origins, *ARS720* being slightly weaker than *ARS719*, corresponding to our lower licencing probability *q*_4_ = 0.93 (median, upper/lower quartiles 92%, 94%). The time series suggests that *O*_3_ is the earliest to fire which is reproduced by our analysis; specifically *O*_3_ fires earlier than *O*_1_, *O*_2_, *O*_4_, Supplementary Figure S18, such that its fork travels 9, 12 and 0.4 kb on average before the others fire, respectively.

**Table 1.** Inferred origin characteristics *ARS717-20*

	*O*_1_	*O*_2_	*O*_3_	*O*_4_	*O*_1_ *O*_2_	*O*_2_ *O*_3_	*O*_3_ *O*_4_
Mean μ	958	5625, 7842	−6581, −3741	−4101	4667	−12205,−11582	−360
S.D. μ	526	345,407	457,199	285	770	585, 535	341
Mean σ	7295	7880, 14 833	12 728, 3345	3340	10 873	15 050,15 138	4749
S.D. σ	1302	1268, 520	1061,278	285	620	556,503	252
Mean *q*	0.75*	1	0.99,1	0.93*			
S.D. *q*	0.02*	0	0.01,0	0.01*			
Mean π(*t*_*i* + 1_ < *t*_*i*_)					0.33	0.79,0.78	0.47
S.D. π(*t*_*i* + 1_ < *t*_*i*_)					0.03	0.01	0.03

Posterior mean and S.D. of the firing time parameters μ*_i_* and σ*_i_* for origin O*_i_*, and their differences between pairs of neighbouring origins. Time measured in rbp. For each triplet ∑*_i_*μ*_i_* = 0 (up to sampling error) because of the normalization of realized firing times to sum to zero. The origin licencing probability *q_i_* is given in row 5, with S.D. (row 6), given as 0 when the MCMC output was degenerate (*q_i_* always 1). * indicates licencing probabilities significantly different from 0 and 1 (*P* < 0.05 assuming a normal distribution, fitting the mean and variance). The mean and S.D. of the probabilities π(*t*_*i* + 1_ < *t_i_*), *i* = 1, 2, 3 are computed for each neighbouring pair of origins based on a Gaussian model with mean μ_*i* + 1_ − μ*_i_* and S.D. $\sqrt{\sigma _i^2+\sigma _{i+1}^2}$. For pair *O*_2_*O*_3_ the first value is inferred from the triplet *O*_1_*O*_2_*O*_3_ and the second one from *O*_2_*O*_3_*O*_4_.

We note that there are only slight differences between the inference based on the overlapping triples *O*_123_ and *O*_234_; the profile between *O*_2_*O*_3_ and the firing time differences between *O*_2_ and *O*_3_ can be reconstructed from both triplets *O*_123_, *O*_234_ and are practically indistinguishable, Figure 3A. Thus, both analyses indicate that *O*_2_, *O*_3_ are a strong pair of neighbouring origins, with two partially licenced neighbours, all firing at similar times. Because obscuring is low, the region between *O*_2_ (*ARS718*) and *O*_3_ (*ARS719*) can be considered almost independent of the influence of the forks coming from *ARS717* and *ARS720*. Thus, comparisons of the inference algorithm on this region from the left and right triplets are predominantly consistent. Of note is that *O*_3_ is not licenced 1% of the time on the left triplet, whilst on the right triplet failure to licence is near zero. This is reconciled by the fact that there is an increase in background fragmentation *b* by 1% in the right triple; these effects correspond to shifting the section *O*_2_*O*_3_ in the OF profile up/down relative to neighbouring regions. Resolving whether this small shift in parameters is due to a normalization problem or an inadequacy of the model will require further work. Finally, we note that the origin location for *ARS719* (*O*_3_) seems too far to the right. However, simulation-based investigation of origin misplacement of up to 5 krbp showed the inference of firing times and licencing probabilities are robust, robustness increasing with the profile simulation size *M* (data not shown).

### Triplets with higher obscuring rates: *ARS813-18*

**Figure 5.**
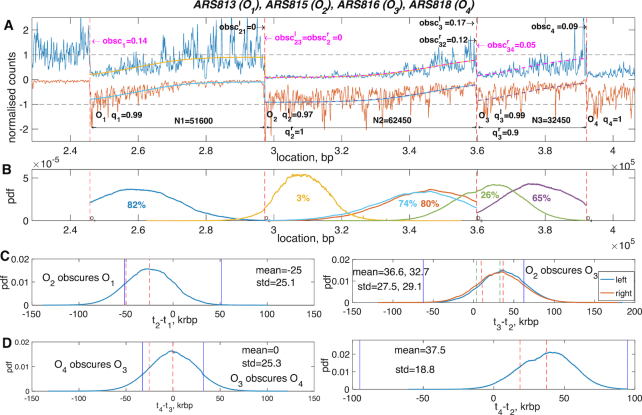
Relative firing times for origins *ARS813*-*ARS818*. (**A**) Annotation of profile with licencing and abscuring probabilities. (**B**) Probability density distributions of the realized collision points between origins. (**C**) and (**D**) Inferred distributions of the firing time differences between neighbouring origins conditioned on origin licencing. See Figure 3 for notation. Inference based on a single converged MCMC run with a burn-in 100 000 and 160 000 samples post burn-in. For licencing and obscuring histograms see Supplementary Figure S20, and Supplementary Table S1 for posterior parameters.

The OF profiles in this case, Figure 5A, show a number of distinct features not present in the previous example. In particular the profiles are not as sharp, with a large flat region near *O*_2_ and distinct gradients at *O*_1_ and *O*_3_ indicating that forks from *O*_2_ are obscuring *O*_1_, *O*_3_, i.e. *O*_2_ must fire earlier than its neighbours sufficient for its forks to reach *O*_1_, *O*_3_. Our model fit substantiates this, the inferred profile highlighting these trends, Figure 5A, whilst the fork collision distributions have truncated tails at *O*_1_, *O*_3_ and *O*_4_, Figure 5B indicative of high levels of obscuring. Obscuring of *O*_1_ (by *O*_2_) is 14% (median, lower/upper quartiles 13%, 15%), *O*_3_ by *O*_2_ is 17% (median, lower/upper quartiles 16%, 17.5%) (left triple analysis), the latter being split in the right triple analysis into 11.6 and 4.4% (median values) obscuring of *O*_3_ by *O*_2_ and *O*_4_, respectively, Figure 5C (overall obscuring of *O*_3_ quartiles on right triple are 13, 16, 19%). These obscuring probabilities are all significantly different than zero *P* < 10^−10^ (using a Gaussian approximation to the obscuring probability distribution, fitting the mean and variance). The firing time distributions, Figure 5C, clarifies that *O*_2_ fires earlier than the others by 25 krbp (*O*_1_), and 37 krbp (*O*_3_, left triple analysis). Further, *O*_2_ fires at least 84% of the time earlier than its neighbours when licenced, Supplementary Table S1. Origin obscuring is a population phenomena, obscuring occurring in a fraction of cells due to stochasticity in the firing times. Thus, although the distances between origins *O*_1_*O*_2_ and *O*_2_*O*_3_ are relatively large, the firing time differences have a large variation with a S.D. of the same order as the mean difference. This results in *O*_2_ obscuring its neighbours only in a fraction of the replicated cells. Our inference is consistent with the time series data of (16), which indicate that *O*_2_ (*ARS815*) is the strongest origin and that *O*_3_ (*ARS816*) and *O*_4_ (*ARS818*) fire later at approximately the same time, see median replication time reconstruction in Supplementary Figure S18.

Although reconstruction of the OF profile is practically identical on triples *O*_123_ and *O*_234_, Figure 5A, there are small differences in their common parts. For instance, the triplet *O*_234_ does not appear to contain sufficient information to unravel the obscuring events from partial licencing of *O*_3_; hence their broad distributions, Supplementary Figure S20.

### Early, poorly licenced origin: *ARS207.5, ARS207.8, ARS208*

**Figure 6.**
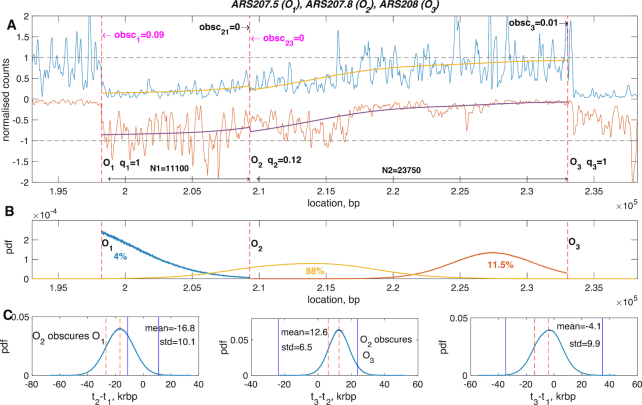
Replication profile for a weak origin: *ARS207.5* - *ARS208*. Upper panel: The data on the forward (blue) and reverse (red) strands and the reconstructed fragment profiles (yellow and purple, respectively). Dashed vertical lines determine the locations of the three origins *O*_1_, *O*_2_, *O*_3_. Inferred licencing probability *q*_*i*_ of origin *i*; inferred obscuring rate *obsc*_*ij*_ of the *i*th origin by *j*th one. In case of the end origin the only subscript is *i*. Arrows next to obscuring rates indicate the direction of the replication fork coming from the neighbouring origin. Magenta text corresponds to forks replicating the forward strand in OF fragments, black text corresponds to forks replicating the forwards strand continuously. Middle panel: Probability density functions of the realized collision points between *O*_1_ and *O*_2_ (blue), *O*_2_ and *O*_3_ (red) and *O*_1_ and *O*_3_ (yellow). Lower panel: probability density plots of the firing time differences between *t*_2_ and *t*_1_ (left panel), *t*_3_ and *t*_2_ (middle panel), *t*_3_ and *t*_1_ (right panel). Vertical blue lines divide the plots into three parts, where the middle part corresponds to the case of no obscuring, the left—to the obscuring of the left origin by the right one, and the rightobscuring of the right origin by the left one; mean is the mean and std is the S.D. of the firing time difference (vertical dashed lines). Only the firing time differences where both origins were licenced are taken into account. Vertical dashed lines. Inference based on a single MCMC run with burn-in 100 000, and 260 000 samples post burn-in.

In this example, the profile shows little evidence of an origin at *ARS207.8* (classified as confirmed in OriDB), Figure 6A. We investigate whether our algorithm can estimate the characteristics of such weak origins. We determined that the licencing probability of the middle origin is very low (median 12%, lower/upper quartiles 11%, 13% but significantly different from zero (*P* < 10^−30^, using a Gaussian approximation, *N*(0.12, 0.01), for the *q* posterior distribution, Supplementary Figure S21). Thus, the majority of the time the forks coming from *O*_1_ (*ARS 207.5*) and *O*_3_ (*ARS208*) collide between these two origins, Figure 6B. However, when *O*_2_ is licenced it fires earlier than either *O*_1_ and *O*_3_ which fire at roughly similar times, Figure 6C, in fact earlier than both neighbouring origins at least 95% of the time, Supplementary Table S2. This results in significant obscuring of its neighbours, specifically with only 12% licencing *O*_2_ obscures *O*_1_ 8.6% (median, lower/upper quartiles 8%, 9%) of the time and *O*_3_ 0.5% (median, lower/upper quartiles 0.2%, 0.7%) of the time. Therefore, the collision point distributions *O*_1_*O*_2_ and *O*_2_*O*_3_ are truncated Gaussian distributions, Figure 6B. This example shows poor correlation with (16), Supplementary Table S4, suggesting that the origin has different activation statistics in the (21) data set; this is also suggested by the high licencing of the end origins *ARS207.5, ARS208* in (15), Supplementary Table S4.

### Analysis of chromosome 10

**Figure 7.**
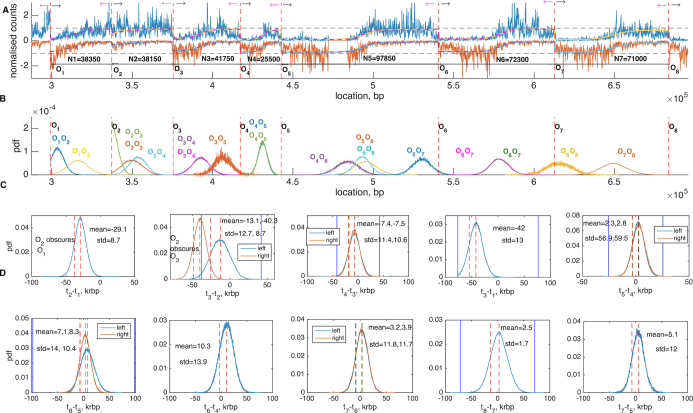
Chromosome 10 analysis: consecutive origins *ARS1010-21*. Analysis of the region between *ARS1010* and *ARS1021* on chromosome 10 obtained by applying algorithm separately on six consecutive triplets including eight origins *ARS1010, ARS1011, ARS1013, ARS1014, ARS1015, ARS1018, ARS1019, ARS1021*. (**A**) Data on the forward (blue) and reverse (red) strands and reconstructed fragment profiles (dashed magenta and purple for the left triplets, solid yellow and blue for right ones). Dashed vertical lines indicate the locations of the eight origins *O*_1_−*O*_8_. Arrows correspond to OF replication forks direction (magenta) and continuous replication forks direction (black) of the forward strand. (**B**) Probability density distributions of the realized collision points between neighbouring origins as indicated, and non neighbours *O*_1_ and *O*_3_ (yellow, lying between *O*_1_ and *O*_2_), *O*_2_ and *O*_4_ (light blue, lying between *O*_2_ and *O*_3_), *O*_3_ and *O*_5_ (orange, lying between *O*_3_ and *O*_5_), *O*_4_ and *O*_6_ (purple, lying between *O*_5_ and *O*_6_), *O*_5_ and *O*_7_ (blue, lying between *O*_5_ and *O*_7_), *O*_6_ and *O*_8_ (yellow, lying between *O*_6_ and *O*_8_). Text-colour corresponds to the distribution of the same colour. (**C**) and (**D**) Inferred distributions of the firing time differences between neighbouring origins conditioned on origin being licenced. Notation as in Figure 3. For licencing and obscuring histograms see Supplementary Figure 23. Inference based on a single MCMC run with burn-in 100 000, and 100 000 samples post burn-in. See Supplementary data for MCMC information (Supplementary Data S1.9).

We analysed most of the chromosome 10 from genome position 64 to 683 817 (92% of the chromosome). Analysis of eight consecutive origins from genome positions 298 471 to 683 817 (52% of chromosome) is summarized in the Figure 7 and the analysis of 10 consecutive origins (position 64 to 298 471) is summarized in the Supplementary Figure S26. Our analysis indicates that origins between 298 471 and 683 827 are predominantly strong non-interfering origins except at the far left where *O*_2_ (*ARS1011*) is a weak origin. Thus, fork termination distributions between neighbouring origins in section *O*_3_—*O*_8_ lie between the origins and obscuring is negligible, Figure 7B and Table 2. licencing is high at >80% but not 100%, giving rise to fork terminations between non-neighbouring origins. The firing time differences are all approximately centred around zero, with mean firing time difference < S.D. (firing time difference) < 0.25 (*N*_*i* + 1_ − *N*_*i*_), giving rise to the observed sharp profiles over *O*_3_−*O*_8_, Figure 7A. Overlapping triples in this region predominantly agree with each other.

**Table 2.** Annotation for the mean obscuring and licencing probabilities for each of the six consecutive triplets

	*O*_1_ *O*_2_ *O*_3_	*O*_2_ *O*_3_ *O*_4_	*O*_3_ *O*_4_ *O*_5_	*O*_4_ *O*_5_ *O*_6_	*O*_5_ *O*_6_ *O*_7_	*O*_6_ *O*_7_ *O*_8_
*q*_1_	1	–	–	–	–	–
*q*_2_	0.26	1	–	–	–	–
*q*_3_	1	0.87	0.87	–	–	–
*q*_4_	–	1	1	1	–	–
*q*_5_	–	−	0.9	0.93	0.99	–
*q*_6_	–	–	–	1	0.95	0.96
*q*_7_	–	−	–	–	0.99	1
*q*_8_	–	–	–	–	–	0.9
obsc1	0.04	–	–	–	–	–
obsc21	0	–	–	–	–	–
obsc23	0.01	0.52	–	–	–	–
obsc32	0	0	–	–	–	–
obsc34	–	0	0	–	–	–
obsc43	–	0	0	–	–	–
obsc45	–	–	0	0	–	–
obsc54	–	–	0	0	–	–
obsc56	–	–	–	0	0	–
obsc65	–	–	–	0	0	–
obsc67	–	–	–	–	0	0
obsc76	–	–	–	–	0	0
obsc8	–	–	–	–	–	0

q*_i_* corresponds to licencing probability of *O_i_*, obsc*ij* corresponds to the probability of the origin *O_i_* being obscured by the origin *O_j_* (note that. *O_1_* and *O_8_* can be obscured only from one side).

The origin *O*_2_ shows significant mismatch between the analyses on triples *O*_123_ and *O*_234_. It is a weak origin on both analyses, but triple *O*_123_ indicates it has a low licencing and thus the profile between *O*_12_ is a consequence of fork collisions between *O*_1_ and *O*_3_. This suggests that region *O*_23_ cannot be understood correctly without region *O*_12_ (on triple *O*_234_ origin *O*_2_ is denoted as highly obscured by *O*_3_). Thus, *O*_2_ is likely a poorly licenced origin, with *O*_1_ a late origin, as inferred on triplet *O*_123_.

**Figure 8.**
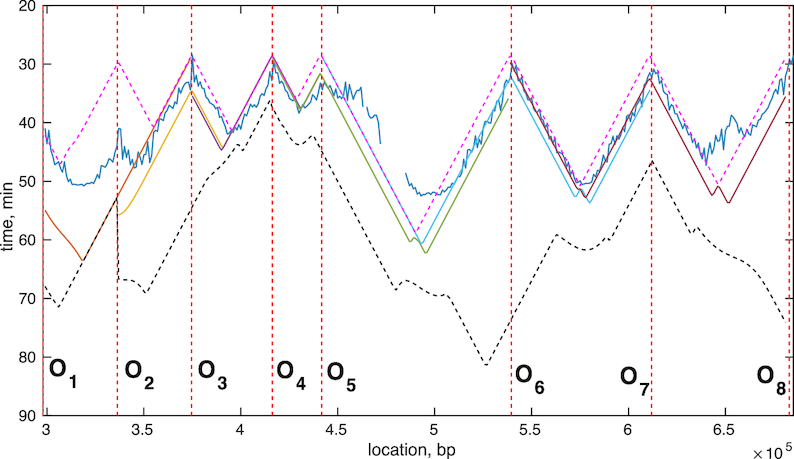
Genome median replication times in chromosome 10. Median replication times *T*_*rep*_ across chromosome 10 as estimated in (34) (blue) and the median replication times derived from our inferred parameters for the region *ARS1010-1021* (different colours correspond to different triplets). Locations of origins given as vertical dashed lines. 5th (pink) and 95th (black) percentiles are given as dashed lines. See Supplement S1.13.

Replication times across this region can be reconstructed from the inferred (median) time differences; since we have only time differences we do not have an absolute time scale within S-phase, so it would be natural to expect mismatch between the reconstruction obtained by using our model and the experimental data. Our reconstructed replication time is compared with the time series data in (34), Figure 8. Here, we matched the earliest origin firing time and scaled the time by the fork speed ($\nu = 1.6$ kb/min reported in (16)), but otherwise our reconstructed firing time profile is independent of the time series data. The reconstruction captures the main features of DNA replication timing, in particular the reconstructed replication time around the early origins is excellent (recall only the time of the earliest origin is matched) but poor on some of the later replicated regions. The mismatch on the far right between *ARS1019* and *ARS1021* may be due to the intervening weak origin *ARS1020*, the time series data of (34) suggests it is active but there is no evidence of it being active in the OF profile so we have not included it in the analysis. Mismatch can also occur because the time series were only taken at 25, 30, 35, 40, 45, 50, 90 min; the time course data at 50 min indicate that the copy number has not exceeded 50% across the genome, failing to reach 50% ∼325 and 500 kbp, Supplementary Figure S33. Thus, replication is incomplete in >50% of cells by 50 min at some locations, whilst the median replication time estimated in (34) suggests replication is more complete. Similarly in case of *ARS1001-1011* (Supplementary Figure S26D) there is some mismatch between 250 and 300 kbp, where the copy number hardly reaches 20% by 50 min. In general, the time series reconstruction across *ARS1001-1011* of chromosome 10 exhibits broad termination zones and good agreement with experimental data of (34), Supplemetary Figure S26D.

In the Supplementary data (Supplementary Figure S26, Supplementary Table S3) we demonstrate that our algorithm works well not only in case of highly licenced non-interfering origins but also when there is a lot of obscuring present. For example there is an obscuring of *ARS1007* 60% of the time (*O*_5_) by *ARS1006* (*O*_4_), 20% of *ARS1007.5* (*O*_6_) by *ARS1008* (*O*_7_), more than 30% of *ARS1009* (*O*_8_) by *ARS1008* (*O*_7_). This part of the chromosome 10 also exhibits lower licencing values. The agreement between the triplets is very good.

**Figure 9.**
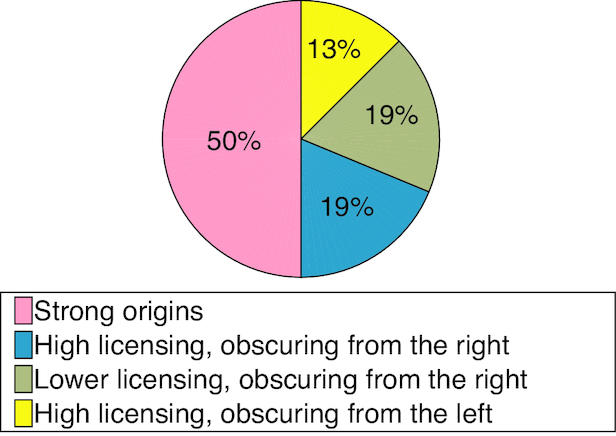
Statistics of licencing and obscuring occurrence for chromosome 10.

Overall 50% of the analysed origins on chromosome 10 are non-obscured and highly licenced origins, with average inferred value of licencing higher than 0.9, 12.5% of the origins are highly licenced and obscured from the left more frequently than from the right, 18.75% of origins are highly licenced and obscured more frequently from the right than from the left, and 18.75% of origins exhibit lower values of licencing (less than 0.9 on average) and are obscured more frequently from the right than from the left (Figure 9).

### *rat1-1* inactivation reduces licencing and increases obscuring

**Figure 10.**
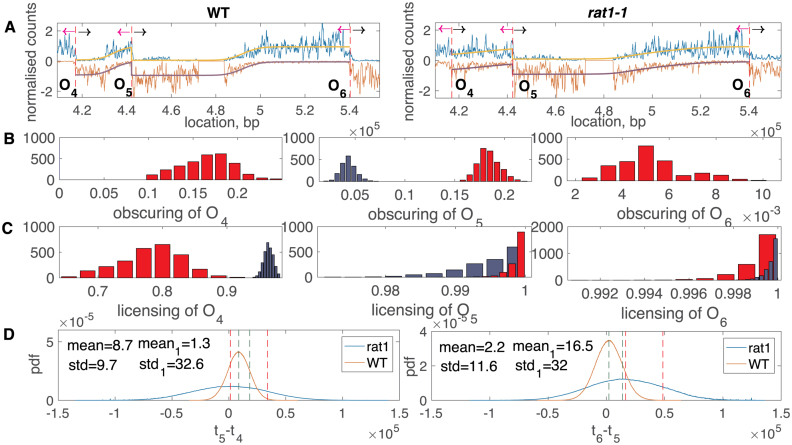
Wild–type and *rat1-1* inactivation data for *ARS1015* (*O*_4_), *ARS1018* (*O*_5_), *ARS1019* (*O*_6_). Comparison of wild–type and *rat1-1* mutant single end sequencing data. Origin labels *O*_*i*_ refer to region analysed in Supplementary Figure S29. (**A**) Profile reconstructions for WT (left panel) and *rat1-1* (right panel). Arrows correspond to OF replication forks direction (magenta) and continuous replication forks direction (black) of the forward strand. (**B**) obscuring data. (**C**) licencing data. Parameters inferred from WT (blue) and *rat1-1* (red). (**D**) *t*_5_−*t*_4_ (left panel) and *t*_6_−*t*_5_ (right panel) distributions inferred from WT (red) and *rat1-1* (blue) plotted with their mean and S.D. values (green and red vertical dashed lines respectively), subscript 1 corresponds to *rat1-1* mutant.

By using a temperature-sensitive *rat1-1* mutant it was demonstrated that the RNA polymerase (RNAP), when not prevented from moving through origins (by *rat1-1*), moves the origin in the direction of transcription. Shifts are ∼2 and 0.5 kb in the direction of transcription on the forward and reverse strands, respectively, (24), negligible for our analysis relative to the distance between origins. The OF profile shows distinct changes indicating that origin efficiency is reduced by collision with the RNAP. Here, we analysed this loss of efficiency in terms of the origin licencing probability and its probability of being obscured. We examined chromosome 10 (336 976 to 683 817 bp, excluding the first weak origin *ARS1010*) where both the WT and the *rat1-1* inactivation strain data were good. As with the previous data set, our model fit allowed inference of all model parameters and their confidence. For instance, in Figure 10 we show the fit for both WT (control) and *rat1-1* inactivation for a typical origin triple. This example shows significant loss of efficiency through obscuring, *O*_4_, *O*_5_, *O*_6_ and a reduction in licencing, *O*_4_ in the *rat1-1* mutant. The increase in obscuring occurs because of the broadening of the firing time distributions, Figure 10D. This dual effect of increased obscuring and loss of licencing was seen across the extended region of chromosome 10, see *ARS1015-19* in Supplementary Figures S27 and S28. Profiles and fork termination distributions are shown for chromosome 10 in Supplementary Figure S29, demonstrating the flatter profiles under *rat1-1* inactivation, and broader termination distributions. Agreement of the analysis between overlapping origins was poorer than in the other data set. On these six origins (pooling results across different triple analyses), obscuring was the greater effect, increasing by 4% (median) under *rat1-1* inactivation, whilst licencing decreased by 0.05% on average. Origin variation was however large with S.D. of 20% for obscuring and 23% for licencing. We also observed that the *rat1-1* inactivation data exhibited higher background noise *b* than the WT, Supplementary Figure S30. This background represents random fragmentation, so it is unclear why this should be the case.

### A region with minimal origin obscuring and strong origins in human cells

The position of DNA replication origins in the human genome is determined by a number of factors and unlike *S. cerevisiae* the question of sequence specificity of origins still remains unresolved (35). Although our algorithm requires that origin positions are specified, it can still be applied to human data when coupled with a origin location algorithm. Using data from (26) (OK-seq) we analysed a region between 98.25 and 99.3 Mb on chromosome 2 of the HeLa cell profiles.

**Figure 11.**
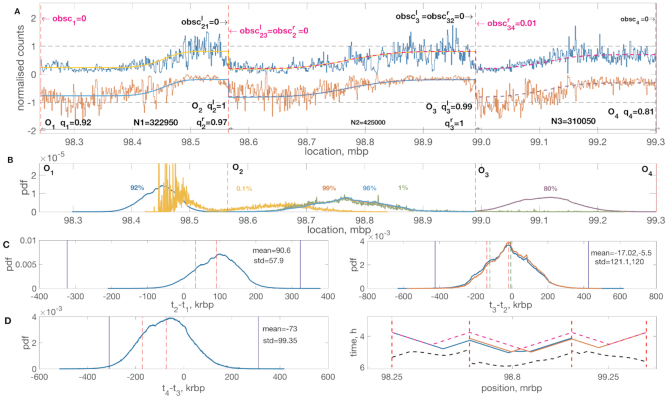
Four strong consecutive human (HeLa) origins, chromosome 2, region 98.25 to 99.3 Mb. Analysis of the region between 98.22 and 99.3 Mb on chromosome 2, HeLa obtained by applying the algorithm separately on the left (*O*_1_, *O*_2_, *O*_3_) and right (*O*_2_, *O*_3_, *O*_4_) triplets. (**A**) Annotation of licencing and obscuring probabilities. (**B**) Probability density distributions of the realized collisions between origins *O*_1_ and *O*_2_ (blue), *O*_2_ and *O*_3_ (red inferred from the left triple, light blue inferred from the right one), *O*_1_ and *O*_3_ (yellow), *O*_3_ and *O*_4_ (purple), *O*_2_ and *O*_4_ (green). Percentages corresponding to the amount of time fork collision were realized (text colour corresponds to the colour of the distribution). (**C**) and (**D**) (left panel): Inferred distributions of the firing time differences between neighbouring origins conditioned on origin being licenced. *t*_2_ and *t*_1_ (C, left panel), *t*_3_ and *t*_2_ (C, right panel) and *t*_4_ and *t*_3_ (D, left panel). See Figure 3 for notation. (**D**) (right panel): Median replication times derived from our inferred parameters for the region 98.25-99.3 Mb for the left (blue) and right (red) triplets. Locations of origins given as vertical dashed lines. 5th (pink) and 95th (black) percentile are given as dashed lines. For licencing and obscuring histograms see Supplementary Figure S31, and Supplementary Table S1 for posterior parameters. Inference based on a single MCMC run with burn-in 100 000, and 100 000 samples post burn-in. See Supplementary data for MCMC information (Supplementary Data S1.1), and Supplementary Table S5 for posterior parameters.

We used the abrupt drop in OF counts at origins to identify origin locations. CUSUM statistics is a standard tool to detect abrupt changes in the behaviour of a sample (36). The human origins detected by CUSUM are in agreement with origin positioning obtained by an alternative origin mapping method (Core origins, SNS-seq, Akerman *et al.*, unpublished results). We detected four strong origins reflecting the three clear tanh-like profiles in this region, Figure 11A. We used our algorithm to analyse the left and the right triple, Figure 11. Our analysis indicates that the 4 origins are all highly licenced with zero, or near zero obscuring rates, the maximum obscuring probability is 0.6% (median, upper/lower quartiles 0.5%, 0.7%) for origin *O*_3_ obscured from the right, whilst the lowest mean licencing probability is 80.5% (median, upper/lower quartiles 80%, 81%) for origin O_4_. The parameters inferred from the left and right triplets demonstrated a very good accordance with each other. The distributions of firing time differences inferred from right and left triplet analysis are almost indistinguishable from one another and indicate that the *O*_2_ and *O*_3_ fire at approximately the same time (Figure 11C, left), probability π(*t*_3_ > *t*_2_) being ∼0.5, whilst *O*_1_ and *O*_4_ fire earlier than *O*_2_ and *O*_3_, respectively: *π*(*t*_2_ > *t*_1_) = 0.9, *π*(*t*_3_ > *t*_4_) = 0.76 (see Supplementary Table S5 for more details). Almost all the time there are three fork termination zones between *O*_1_ and *O*_2_, *O*_2_ and *O*_3_, *O*_3_ and *O*_4_. Terminations between *O*_1_ and *O*_3_, *O*_2_ and *O*_4_ are rare, 0.1% and 1% of the time, respectively, which happens mainly when either *O*_2_ or *O*_3_ are not licenced.

Analogous to previous sections, we also performed analysis of median replication times, Figure 11D, right. In previous studies of the human genome (37,38) results on the median and mean replication times were obtained experimentally, so we compared our inferred replication times with those in (37). In our analysis, we used the fork velocity value of 3.3 kb min^–1^ which is consistent with (37) for that region. The region between origins *O*_2_ and *O*_3_ agrees very well with the analysis shown in the Supplementary data of (37). The range of the median replication times in this region is exactly the same, between 4 and 5 h. Moreover, our analysis also reproduced two local minima in this region which are present in (37) as well. The remaining regions demonstrate poorer agreement with the experimental profiles, although the latter are still within 5–95th percentile of the data and general trends are still there. The mismatch could be caused by a number of reasons. In addition to the ones discussed in section ‘Analysis of chromosome 10’ and the increased complexity of the organism we are dealing with, this is possibly due to a fact that a number of low-efficiency origins which were not strong enough to be detected by a change-point analysis of the OF data were not taken into account, which was also reported as a potential problem in (26). The general patterns, however, of the experimental and inferred profiles are similar. In summary, we demonstrate that our algorithm can be used to analyse origin characteristics in human cells.

## DISCUSSION

We have developed a unique Bayesian analysis methodology for Next Generation Sequencing (NGS) data, directly fitting a generative model to the pile-up data. Our model is stochastic, reflecting population stochasticity of DNA replication; thus, we quantify not only mean population behaviour, but also the sources of biological variability and measurement noise. Our MCMC algorithm tackled two analysis challenges, the first is the statistical tractability of the model (having an intractable likelihood), which we solved by using a suitable approximation; and second, the model dimension varies depending on the number of origins with realized forks, a manifestation of origin interference. This was dealt with through a reversible jump algorithm (39). Our algorithm achieves robust parameter inference in yeast and human data, across different sequencing procedures, using only information from origin triples and not from the whole chromosome.

Our origin firing analysis in *S. cerevisiae* demonstrated that obscuring and partial origin licencing can be distinguished. We provide examples of a triplet of origins with and without origin obscuring, Figures 3, 5 respectively, and partial licencing Figure 6. In general however, both origin obscuring and partial licencing may be present to varying degrees underpinning origin flexibility. Our analysis of chromosome 10 demonstrated that firing time trends across larger regions can be reconstructed, indicating that region 375–684 kb is dominated by strong origins with negligible obscuring whilst the licencing probability of some origins can be as low as 87%. The region 64–300 kb contains origins with higher obscuring rates, including examples with over 50% obscuring, and lower licencing, at times below 80%. Our inferred origin characteristics can be compared to previous fits of this model ((15,16) based on maximum-likelihood methods therefore giving point estimates only) and to independent time series data (34). Our analysis very much accords with previous fits as regards to origin efficiency, Supplementary Table S4, and reconstruction of median replication time is excellent across triples, Supplementary Figure S18 and extended regions, Figure 8, Supplementary Figure S26D. Example ‘Early, poorly licenced origin: *ARS207.5, ARS207.8, ARS208*’ is particularly illuminating, as it in fact discriminates the processes of licencing and firing. Under the MCM loading model, (28), licencing involves loading of MCMs to the origin, whilst the greater number of loaded MCMs the earlier the firing. This directly links licencing efficiency with (earlier) firing time. However, we have an example of an origin that has poor licencing (on the MCM model this would indicate low MCM loading efficiency), but it fires early when licenced (on the MCM model this would indicate a high MCM load). In our model, licencing denotes all processes that are required such that the origin can fire in that replication cycle, and would do so if obscuring from neighbours is prevented. Further analysis is required to both ascertain the link between licencing (MCM loading) and this firing capacity, and determine if this is an isolated example.

Our analysis of *rat1-1* inactivation data (a temperature sensitive mutant) demonstrates that our model is able to unravel subtle phenotypes, decomposing the observed reduction of the origin firing efficiency into an increase in obscuring and loss of licencing. Our analysis, albeit on seven origins, indicates that obscuring causes a greater loss in efficiency, however effects were diverse across our sample of origins indicative of origin-specific dependence. This suggests that the mechanisms that control origin firing are degraded under loss of *rat1-1*, in particular, there is a substantial increase in firing time widths of origins coupled with a loss of licencing, both effects result in an increase in the stochasticity of origin firing. Thus, origins predominantly retain licencing indicating that MCM proteins remain bound (sufficient for licencing) consistent with the *in vitro* data of (24). The small shift in origin (MCM) locations may explain these effects through shifting MCMs from activating factors, such as FKH-Dbf4.

**Figure 12.**
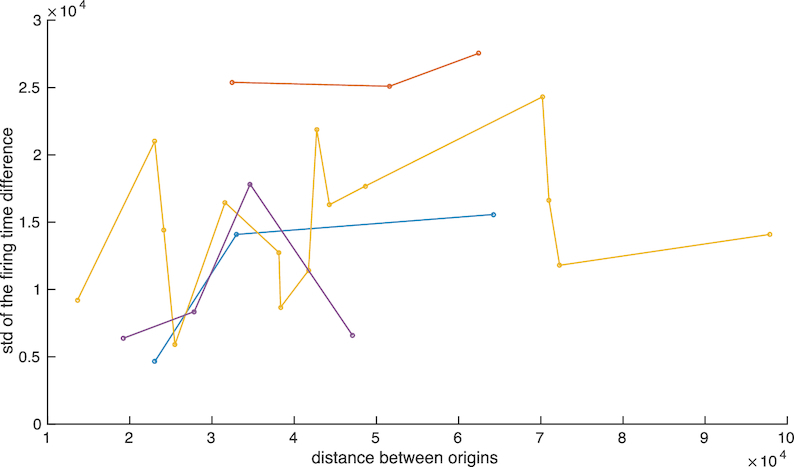
Standard deviation of the firing times of the neighbouring origins versus the distance between them. Colours distinguish different origin sets, i.e. from different chromosomal locations. Total number of triplets 21, with 2 from chromosomes 7 and 8, 3 from chromosome 5 and 14 from chromosome 10.

The source of noise is an important aspect of model-dependent data analysis. We assumed firing time variability, origin licencing and measurement noise dominate, whilst fork speed variability has negligible impact on the OF profile data. A key question is if fork progression adds significant noise; if this was the case it would impart a relationship whereby the S.D. of the firing time difference would increase with distance between origins. Analysis of the time difference variability with distance between neighbours shows that there is no correlation between firing time difference S.D. and origin separation, (*r* = 0.29, *P* = 0.13), suggesting that fork speed variability is negligible, Figure 12. Also, clustering of the firing time difference variability by chromosome is significant (*P* < 0.0001), suggesting that origin firing time variability has a chromosomal dependence.

Our analysis of overlapping triplets typically resulted in good accordance between the inferred parameters. Specifically, we show that in cases when no replication forks come from outside the origin triple our model works very well and does not require information from the whole chromosome. One possible generalization of our algorithm is to extend it to analyse higher numbers of consecutive origins in order to allow for origin interference over greater distances.

Given the variety of technologies that can be used to study DNA replication, the integration of data sets into a single analysis is a natural step forward. Bayesian techniques, as used here, enable this. The first step in this direction would be the integration of the temporal data of (16) with the OF profile data (21), and/or polymerase strand specificity data (40–42). Data integration will enable the power of different techniques to be used to construct a fuller picture, potentially leading to a predictive model. Another potential avenue of development is single cell sequencing which would allow cell variability to be included into the models, for instance origin activation levels may vary between cells, potentially being an important contribution to population stochasticity. Analysis of organisms where origin location is only partially known (35) ideally requires origin location to also be inferred within the Bayesian analysis, to correctly allow for the effect of location error on the other parameters. Finally, the methodology we deploy is very general, and could be extended to the inference of a range of mechanistic problems from sequencing data.

## DATA AVAILABILITY

Implementation of the inference algorithm is available via public repository on GItHub (https://github.com/albazarova/DNAorigins).

## Supplementary Material

Supplementary Data
