# Supplementary material for: Bayesian inference of origin firing time distributions, origin interference and licencing probabilities from Next Generation Sequencing data
Source: Nucleic Acids Res. 2019 Feb 14;47(5):2229–43. doi: 10.1093/nar/gkz094 (PMC6412128; doi:10.1093/nar/gkz094)
Supplement: Supplementary Data [file gkz094_supplemental_files.zip › BazarovaFigsS1-S17.pdf]

S3 Supplementary Figures

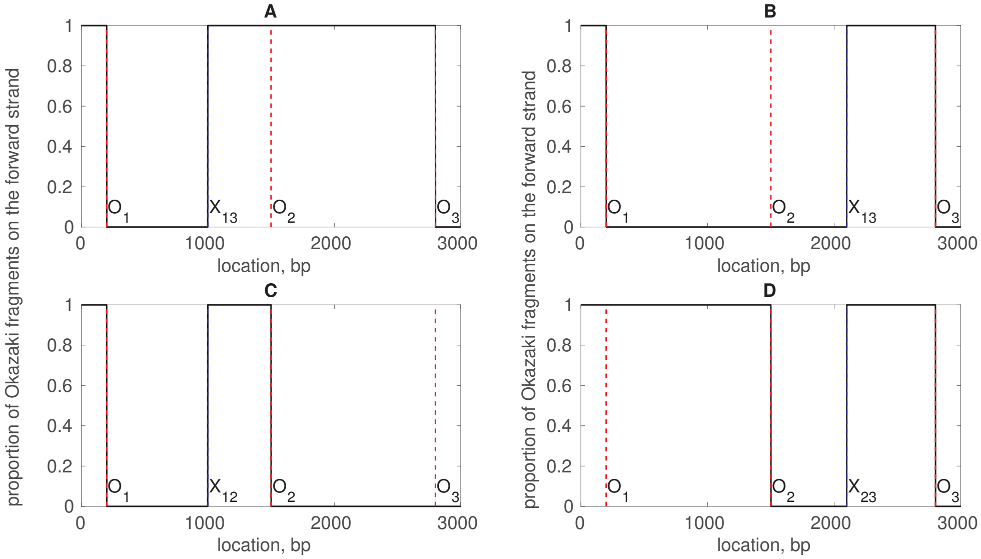

Figure S1 **Examples of replication forks passing through neighbouring origins**. Single genome duplication (forward strand) profiles for 3 origins. Vertical dashed red lines are the locations of the origins O_1 (200 bp), O_2 (1500 bp) and O_3 (2800 bp). Vertical blue lines are the locations of collision points between replication forks. **A**: Middle origin O_2 overrun by the left-moving fork. X_{13} is the collision point of the O_1, O_3 replication forks. **B**: Middle origin O_2 overrun by the right-moving fork. X_{13} is the collision point of the O_1, O_3 replication forks. **C**: Right origin O_3 is overrun. X_{12} is the collision point of the O_1, O_2 replication forks. **D**: Left origin O_1 is overrun. X_{23} is the collision point of the O_2, O_3 replication forks.

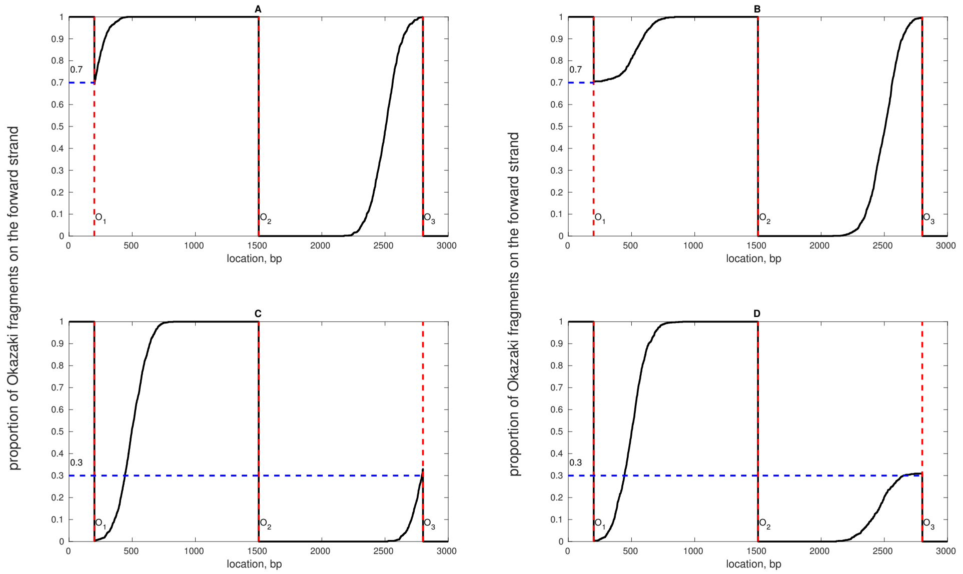

Figure S2 **Examples of OF profile F_{ave} caused by partial licensing and obscuring of end origins**. **A**: Left origin O_1 is obscured in 70% of the cases. Firing time distributions are $t_1 \sim N(800, 100^2)$, $t_2 \sim N(-602, 196^2)$, $t_3 \sim N(100, 100^2)$. **B**: Left origin O_1 is not licensed in 70% of the cases. Firing time distributions are $t_1 \sim N(0, 100^2)$, $t_2 \sim N(-602, 196^2)$, $t_3 \sim N(100, 100^2)$. **C**: Right origin O_3 is obscured in 70% of the cases. Firing time distributions are $t_1 \sim N(100, 100^2)$, $t_2 \sim N(-602, 196^2)$, $t_3 \sim N(800, 100^2)$. **D**: Right origin O_3 is not licensed in 70% of the cases. Firing time distributions are $t_1 \sim N(100, 100^2)$, $t_2 \sim N(-602, 196^2)$, $t_3 \sim N(0, 100^2)$. Vertical red dashed lines indicate locatation of origins at O_1 (200 bp), O_2 (1500 bp) and O_3 (2800 bp). F_{ave} constructed from 5000 duplications.

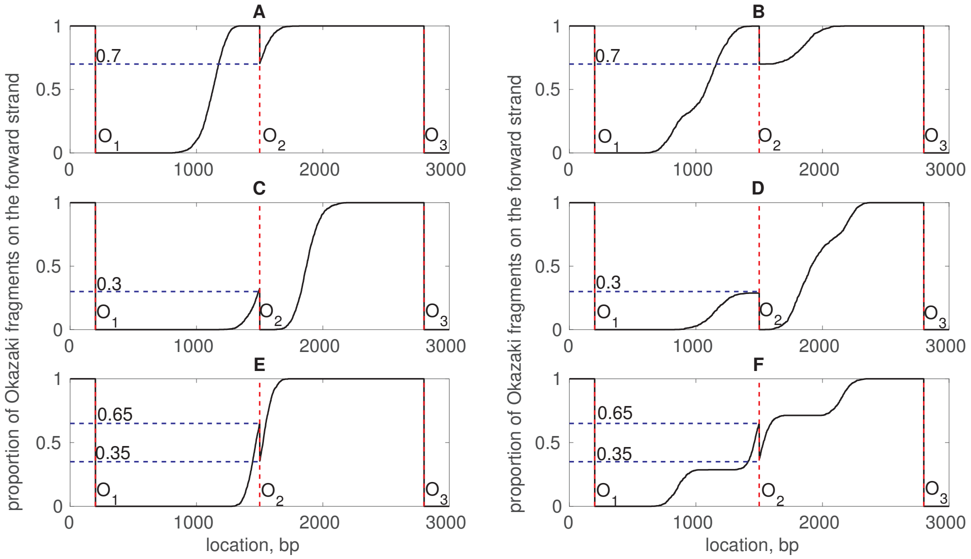

Figure S3 **Examples of OF profiles F_{ave} caused by obscuring and partial licensing of mid origins**. Left panels: obscuring, right panels: partial licensing. **A**: Middle origin O_2 is obscured from the right in 70% of the cases. Firing time distributions are $t_1 \sim N(100, 100^2)$, $t_2 \sim N(800, 100^2)$, $t_3 \sim N(-602, 196^2)$. **B**: Middle origin O_2 is not licensed in 70% of the cases. Firing time distributions are $t_1 \sim N(100, 100^2)$, $t_2 \sim N(0, 100^2)$, $t_3 \sim N(-602, 196^2)$. **C**: Middle origin O_2 is obscured from the left in 70% of the cases. Firing time distributions are $t_1 \sim N(-602, 196^2)$, $t_2 \sim N(800, 100^2)$, $t_3 \sim N(100, 100^2)$. **D**: Middle origin O_2 is not licensed in 70% of the cases. Firing time distributions are $t_1 \sim N(-602, 196^2)$, $t_2 \sim N(0, 100^2)$, $t_3 \sim N(100, 100^2)$. **E**: Middle origin O_2 is obscured from both sides in 70% of the cases (35% from the right and 35% from the left). Firing time distributions are $t_1 \sim N(100, 100^2)$, $t_2 \sim N(1407, 100^2)$, $t_3 \sim N(100, 100^2)$. **F**: Middle origin O_2 is not licensed in 70% of the cases. Firing time distributions are $t_1 \sim N(100, 100^2)$, $t_2 \sim N(100, 100^2)$, $t_3 \sim N(100, 100^2)$. Vertical red dashed lines indicate location of origins: O_1 (200 bp), O_2 (1500 bp) and O_3 (2800 bp). F_{ave} constructed from 5000 duplications.

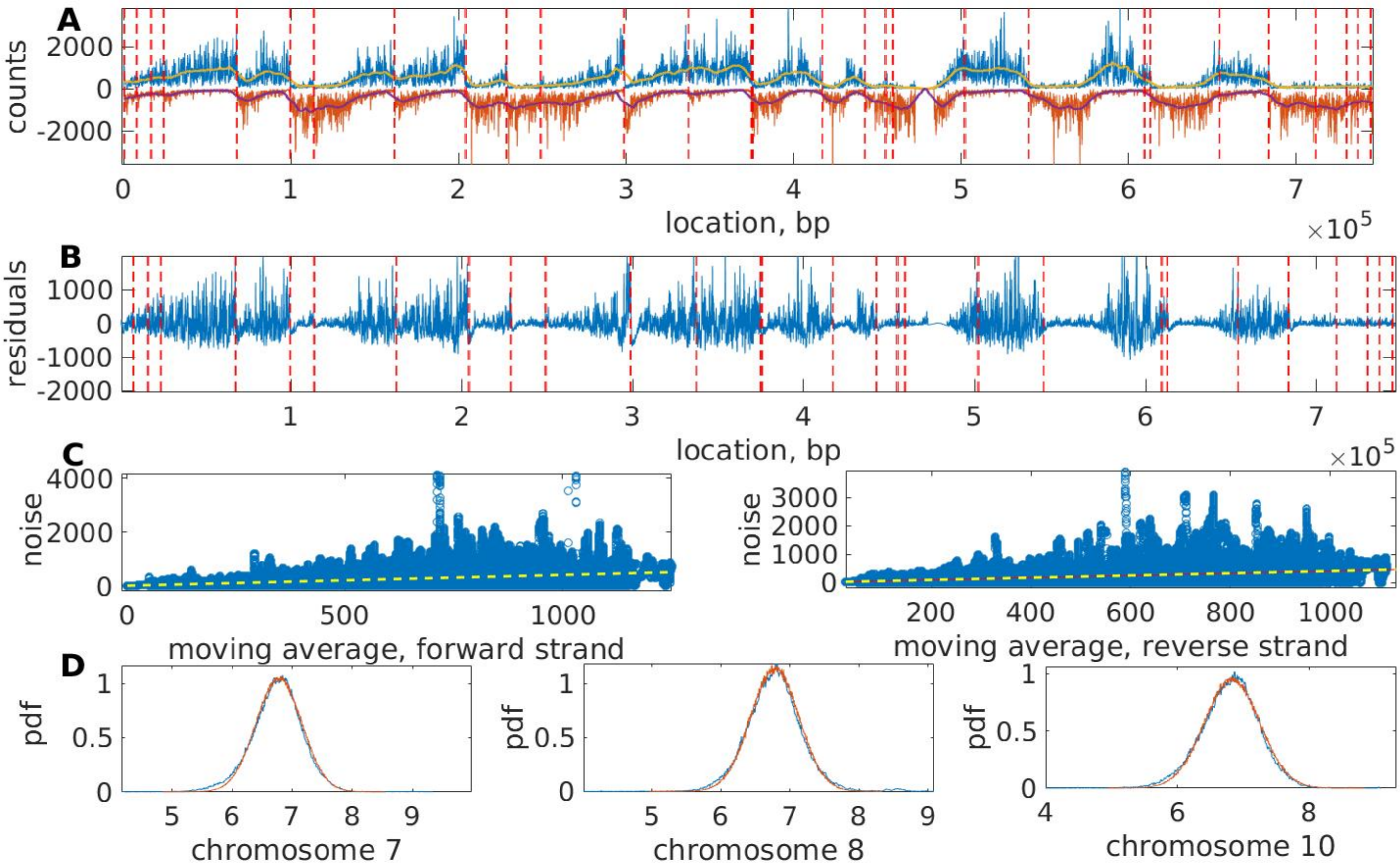

Figure S4 **Noise scales with the signal, chromosome 10.** **A**: data on the forward (blue) and reverse (red) strands and moving average with a window size 1000 (yellow and purple respectively). Vertical dashed lines determine the positions of the origins. **B**: residual on the forward strand of chromosome 10 obtained by subtracting the moving average from the raw data. **C**: scatter plots of the noise (absolute values of residuals) against the signal on the forward (left) and reverse (right) strands and a regression line (dashed). **D**: Distribution of a log-transform of the bias corrected, strand summed read counts data (blue), normal approximation (red) for chromosome 7 (left panel), 8 (middle panel) and 10 (right panel).

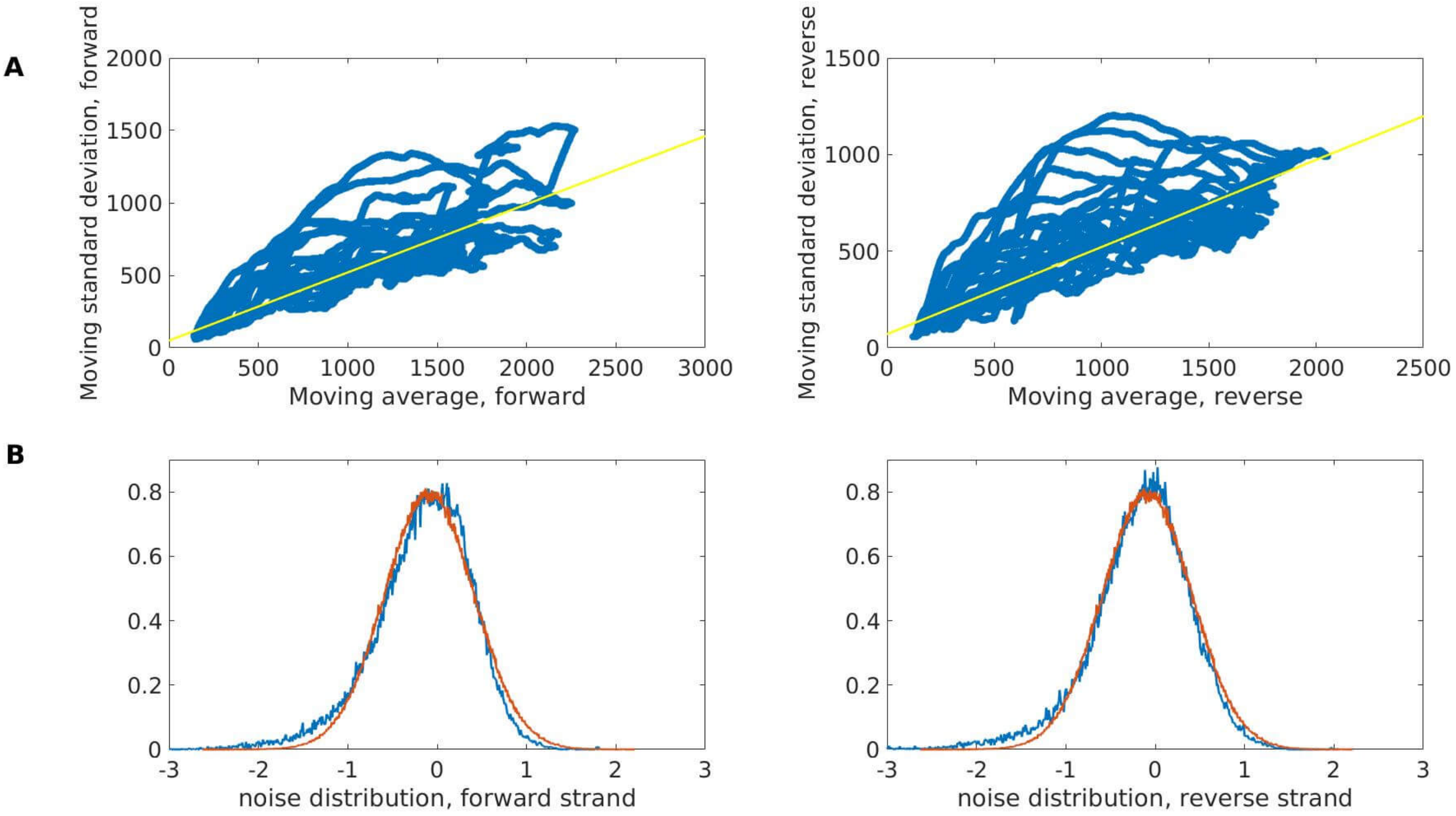

Figure S5 **Log-normal noise, chromosome 10.** **A**: Moving standard deviation vs moving average of the read counts (signal approximation) with a window size 1000 computed across the chromosome 10 on the forward (left panel) and reverse (right panel) strands. Solid yellow line is the least squares line indicating linear relations. **B**: The distribution of logged multiplicative noise (blue line, read counts divided by the moving average) together with its approximation via normal N(-0.09,0.5) (red line) for the forward (left panel) and reverse (right panel) strands.

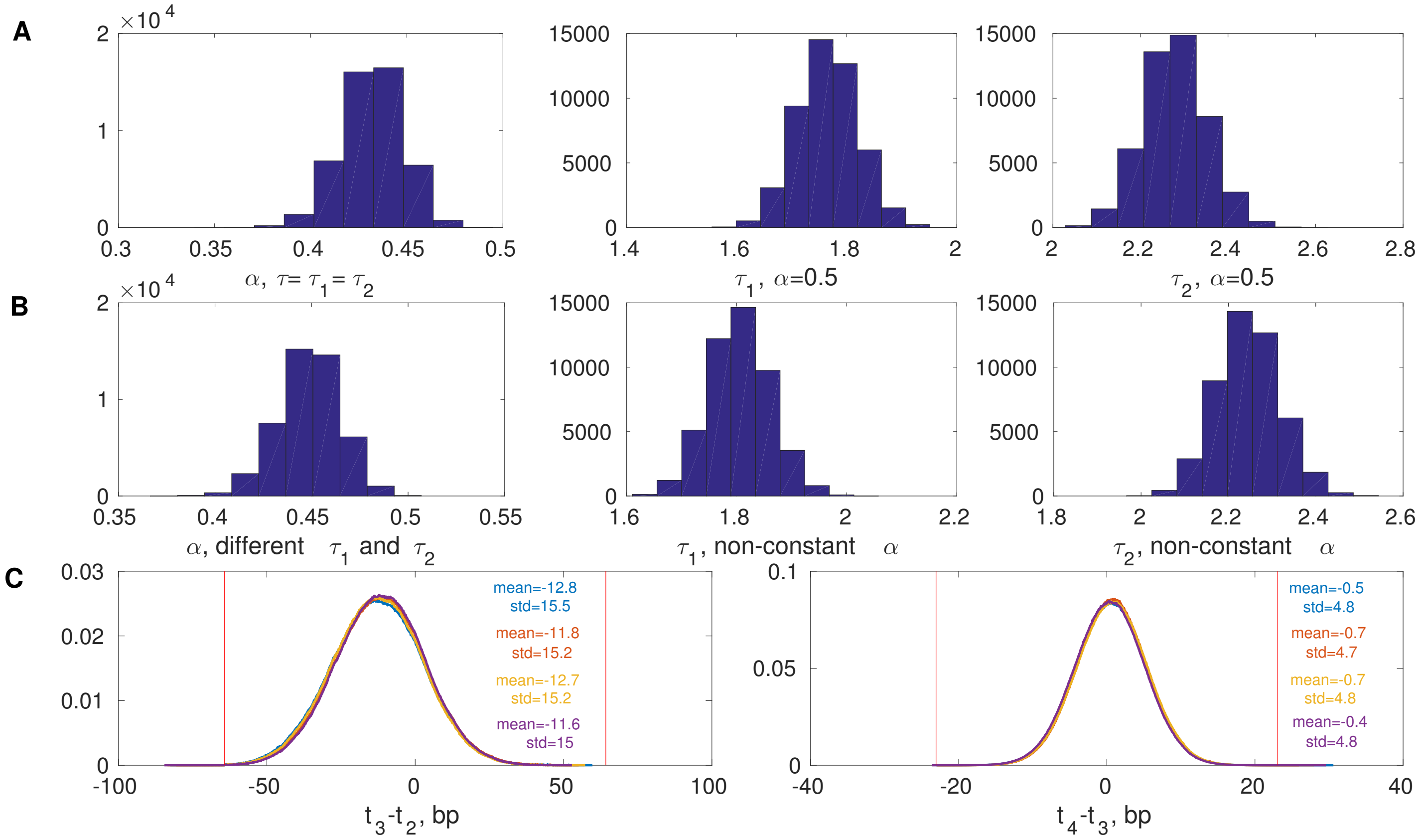

Figure S6 **Inference of noise parameters for generalised model 4 for chromosome 7,** *ARS718-720*. **A, left panel**: Histogram of α for model with $\tau = \tau_1 = \tau_2$. **middle and right panels**: Histogram of τ_1 (middle), τ_2 (right), on model with $\alpha = 0.5$. **B**: Histogram of α (left panel), τ_1 (middle panel), τ_2 (right panel) on fully general model with $\alpha, \tau_{1,2}$ as parameters. **C**: Firing time difference distributions for model with parameter α, $\tau_1 = \tau_2$ (blue), $\tau_1 \neq \tau_2$ and $\alpha = 0.5$ (orange), $\tau_1 \neq \tau_2$, parameter α (yellow), $\alpha = 0.5$, $\tau_1 = \tau_2$ (purple). Inference from on a single MCMC run with a burn-in 100000 and 50000 post burn-in.

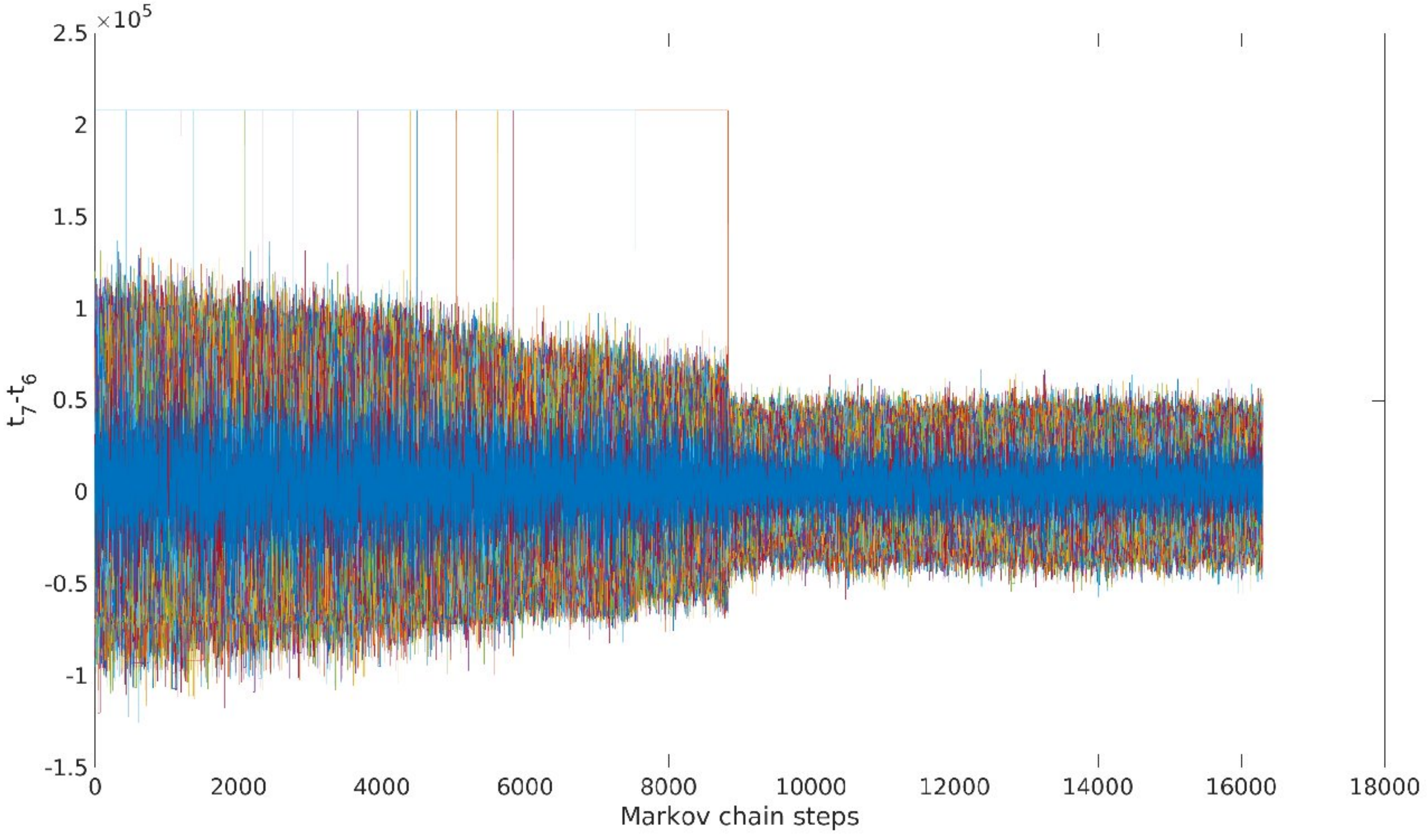

Figure S7 **An example of a converged Markov chain.** Time difference $t_7 - t_6$ for $M = 4992$ realisations, chromosome 10, *ARS1018* and *ARS1019*. Chain is subsampled by 10, first 100000 steps are discarded.

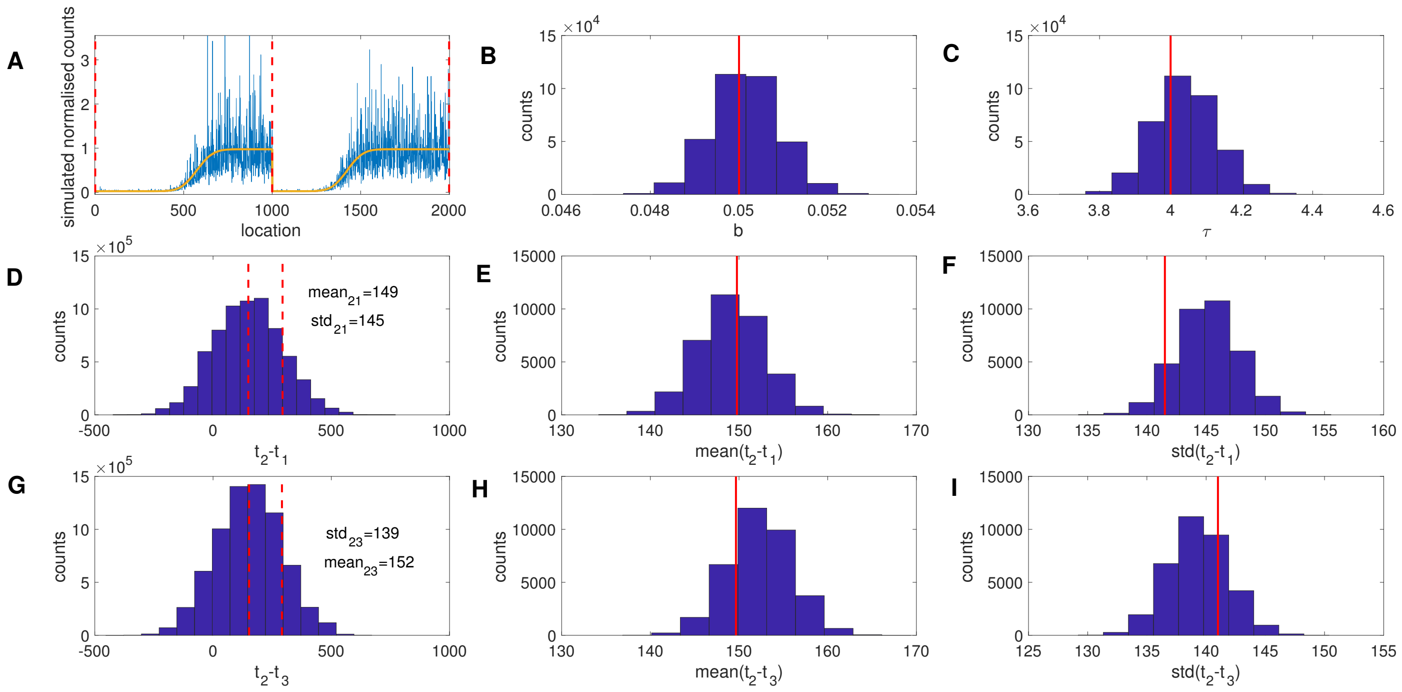

Figure S8 Simulation studies for $t_1 \sim N(-50, 100)$, $t_2 \sim N(100, 100)$, $t_3 \sim N(-50, 100)$, $q_1 = q_2 = q_3 = 1$, $b = 0.05$, $\tau = 4$. **A**:Simulated data (blue), profile reconstruction (yellow), origin locations (vertical dashed lines). **B**: Histogram of b (blue), true value (red line). **C**: Histogram of τ (blue), true value (red line). **D**: Histogram of $t_2 - t_1$, mean of $t_2 - t_1$ (left vertical dashed line), one standard deviation of $t_2 - t_1$ from the mean (right vertical dashed line). **E**: Histogram of mean of $t_2 - t_1$ over M realisations (blue), true value (red line). **F**: Histogram of standard deviation of $t_2 - t_1$ over M realisations (blue), true value (red line). **G**: Histogram of $t_2 - t_3$, mean of $t_2 - t_3$ (left vertical dashed line), one standard deviation of $t_2 - t_3$ from the mean (right vertical dashed line).**H**: Histogram of mean of $t_2 - t_3$ over M realisations (blue), true value (red line). **I**: Histogram of standard deviation of $t_2 - t_3$ over M realisations (blue), true value (red line).

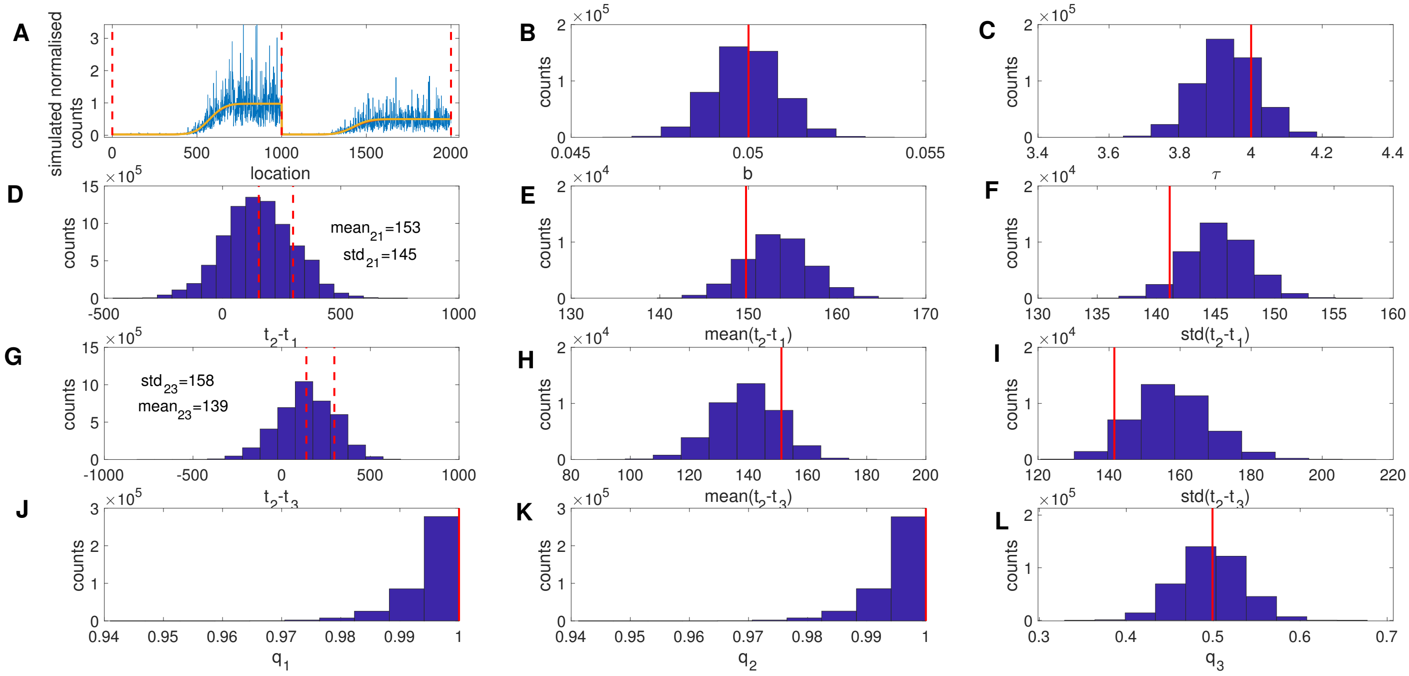

Figure S9 Simulation studies for $t_1 \sim N(-50, 100)$, $t_2 \sim N(100, 100)$, $t_3 \sim N(-50, 100)$, $q_1 = q_2 = 1$, $q_3 = 0.5$, $b = 0.05$, $\tau = 4$. **A - I**: As figure S8. **J**: Histogram of q_1 (blue), true value (red line). **K**: Histogram of q_2 (blue), true value (red line). **L**: Histogram of q_3 (blue), true value (red line).

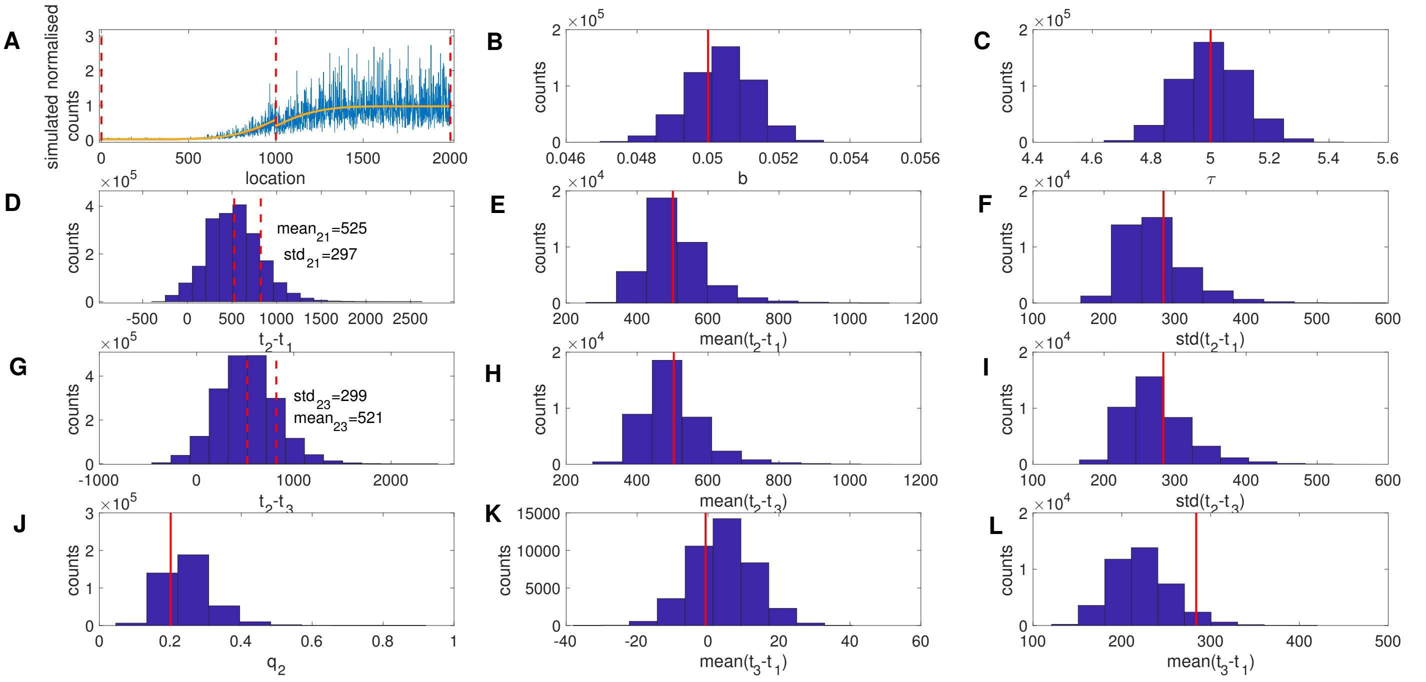

Figure S10 Simulation studies for $t_1 \sim N(-250, 150)$, $t_2 \sim N(250, 150)$, $t_3 \sim N(-250, 150)$, $q_1 = q_3 = 1$, $q_1 = 0.2$, $b = 0.05$, $\tau = 5$. **A - I**: As figure S8. **J**: Histogram of q_2 (blue), true value (red line). **K**:Histogram of mean of $t_3 - t_1$ over M realisations (blue), true value (red line). **L**: Histogram of standard deviation of $t_3 - t_1$ over M realisations (blue), true value (red line).

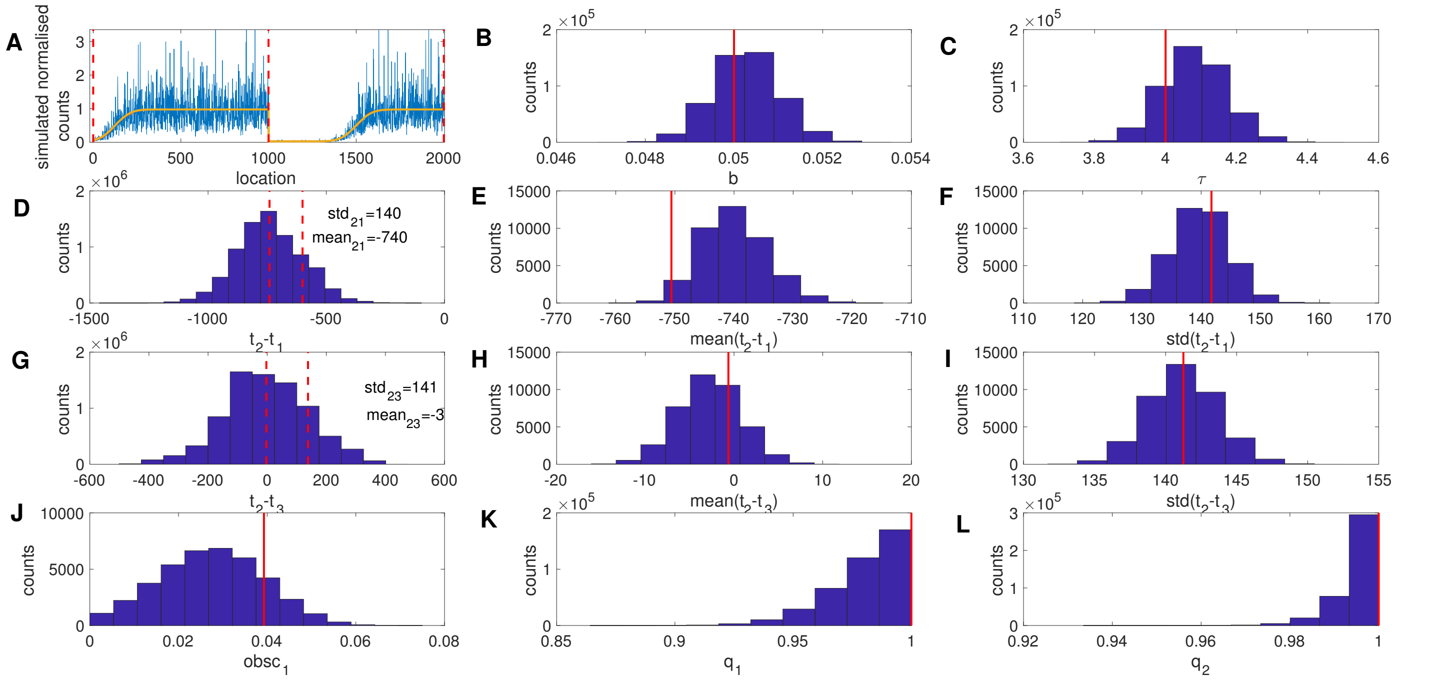

Figure S11 Simulation studies for $t_1 \sim N(500, 100)$, $t_2 \sim N(-250, 100)$, $t_3 \sim N(-250, 100)$, $q_1 = q_2 = q_3 = 1$, $b = 0.05$, $\tau = 4$. **A - I**: As figure S8. **J**: Histogram of obscuring rate of the left origin over M realisations (blue), true value (red line). **K**: Histogram of q_1 (blue), true value (red line). **L**: Histogram of q_2 (blue), true value (red line).

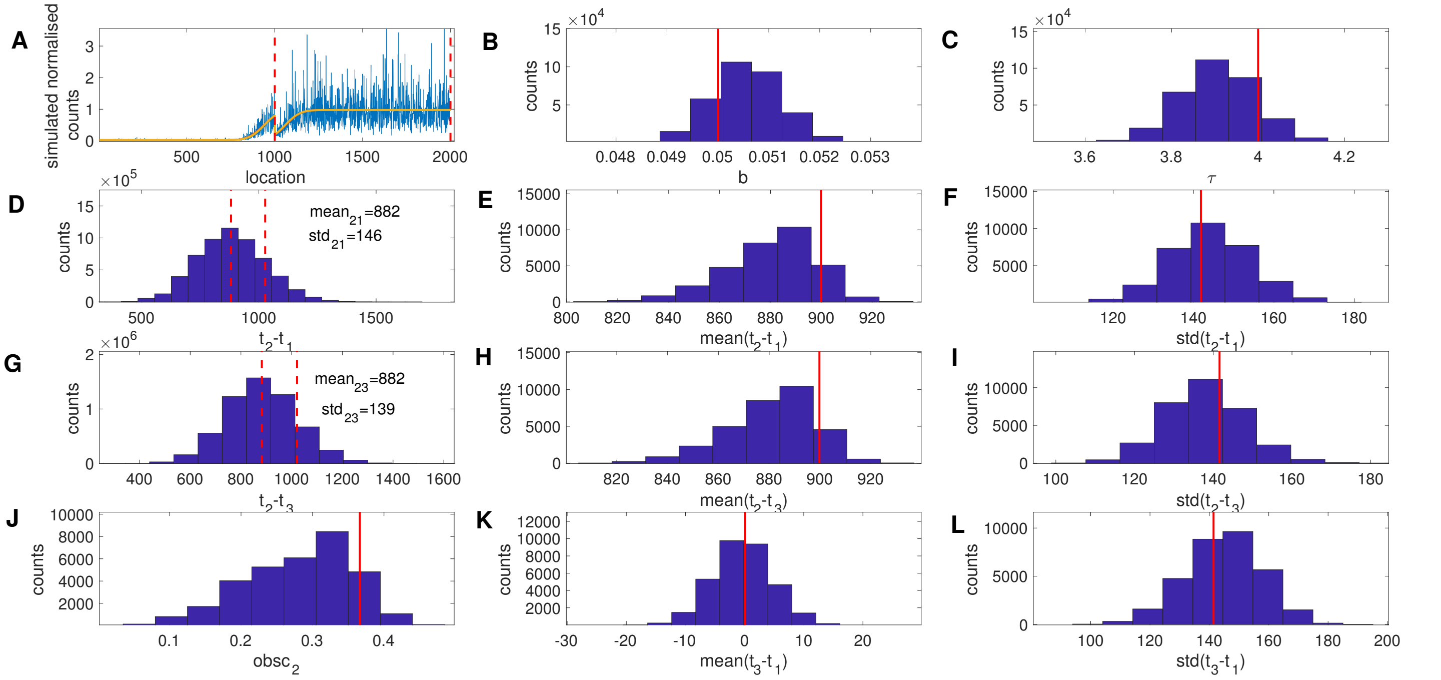

Figure S12 Simulation studies for $t_1 \sim N(-300, 100)$, $t_2 \sim N(600, 100)$, $t_3 \sim N(-300, 100)$, $q_1 = q_2 = q_3 = 1$, $b = 0.05$, $\tau = 4$. **A - I**: As figure S8. **J**: Histogram of obscuring rate of the middle origin over M realisations (blue), true value (red line). **K-L**: As figure S10).

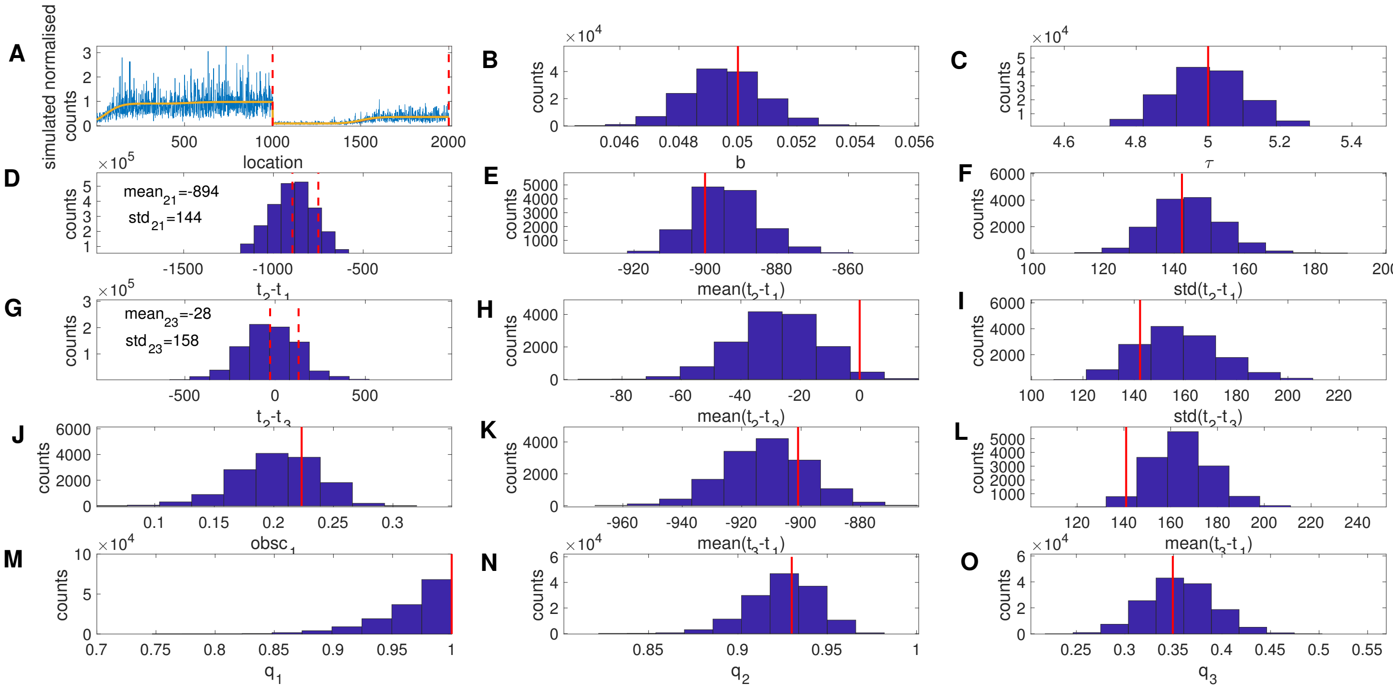

Figure S13 Simulation studies for $t_1 \sim N(600, 100)$, $t_2 \sim N(-300, 100)$, $t_3 \sim N(-300, 100)$, $q_1 = 1$, $q_2 = 0.92$, $q_3 = 0.35$, $b = 0.05$, $\tau = 5$. **A - I**: As figure S8. **J**: Histogram of obscuring rate of the left origin over M realisations (blue), true value (red line). **K-L**: As figure S10). **M - O**: As **J - L** on figure S9.

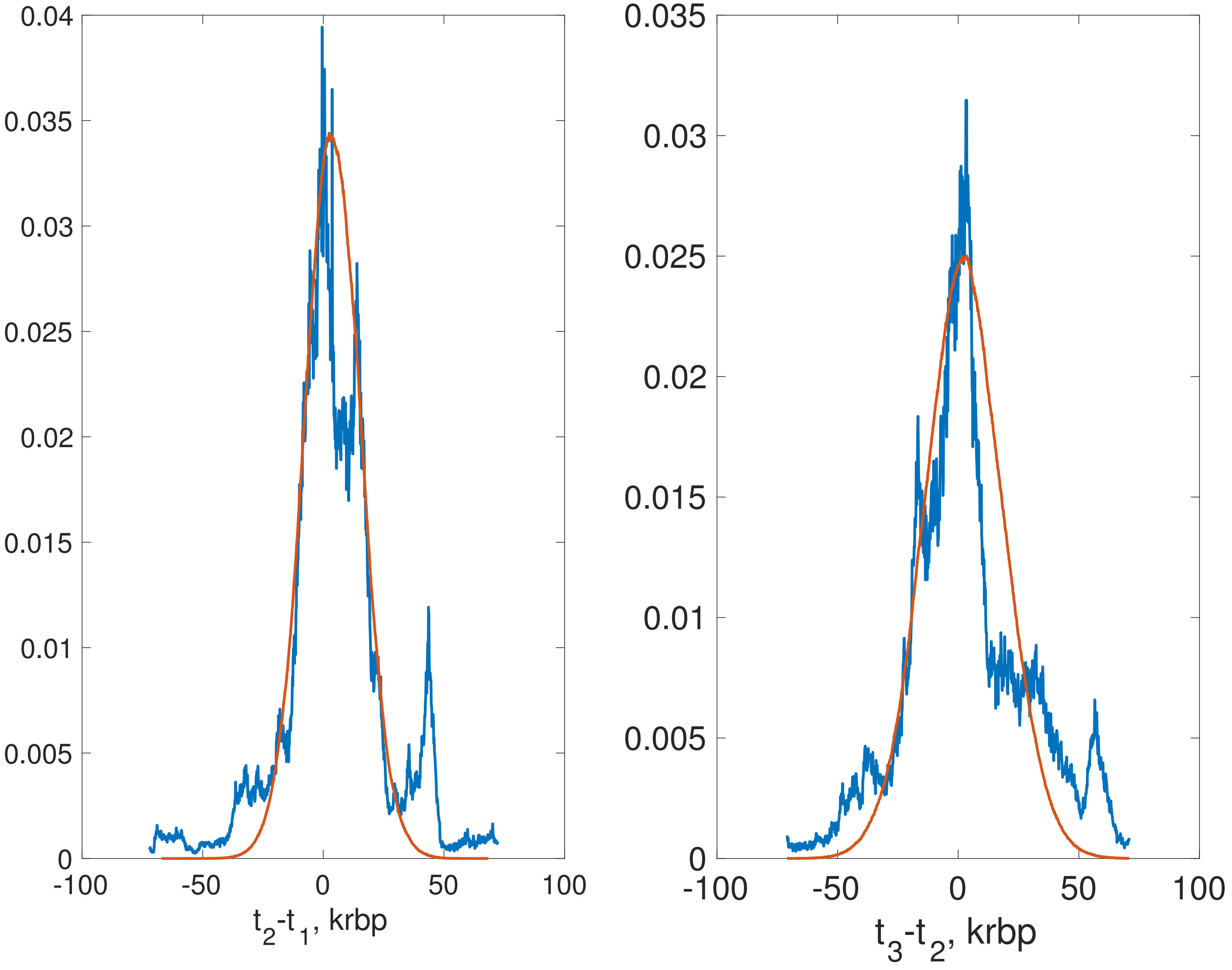

Figure S14 **Priors on firing times, chromosome 10.** Inference of the firing time differences using strong Gaussian (red) and weak, almost flat, (blue) priors. Inference is done for an origin triple *ARS1018, ARS1019, ARS1021.*

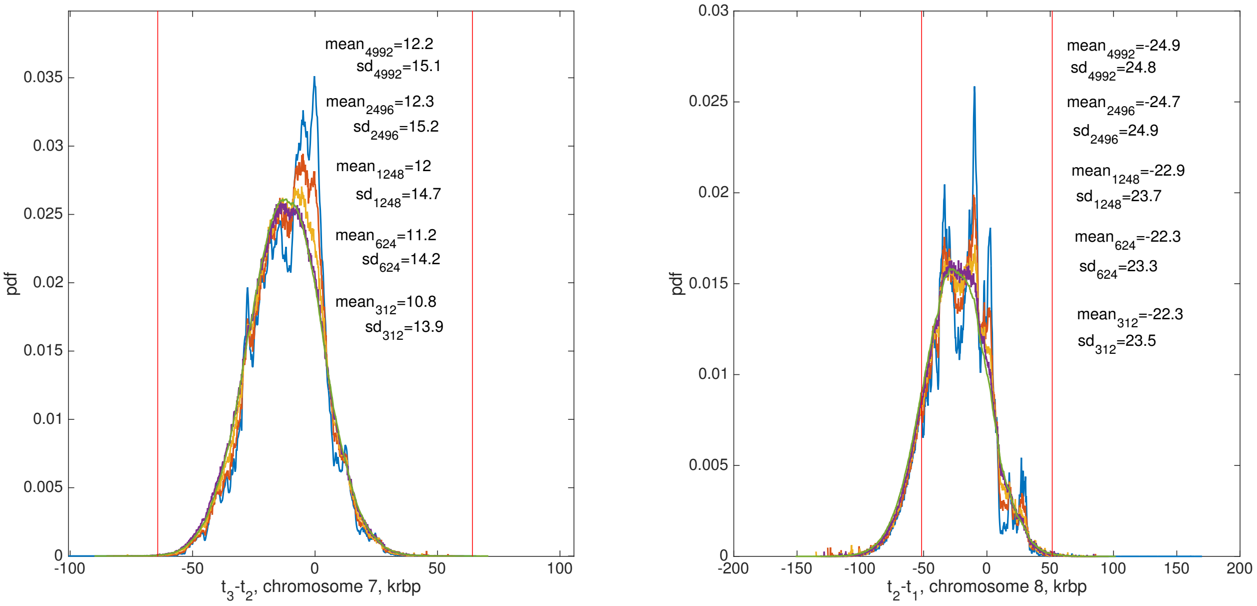

Figure S15 **Distribution of the difference between firing times for different values of M. A:** *ARS719* and *ARS718*, **B:** *ARS815* and *ARS813*. $M = 312$ (blue), $M = 624$ (red), $M = 1248$ (yellow), $M = 2496$ (purple) and $M = 4992$ (green). Vertical red lines indicate obscuring. Lower index correspond to different values of M. Inference based on a single convrged MCMC run with a burn-in 100000 and 100000 samples post burn-in.

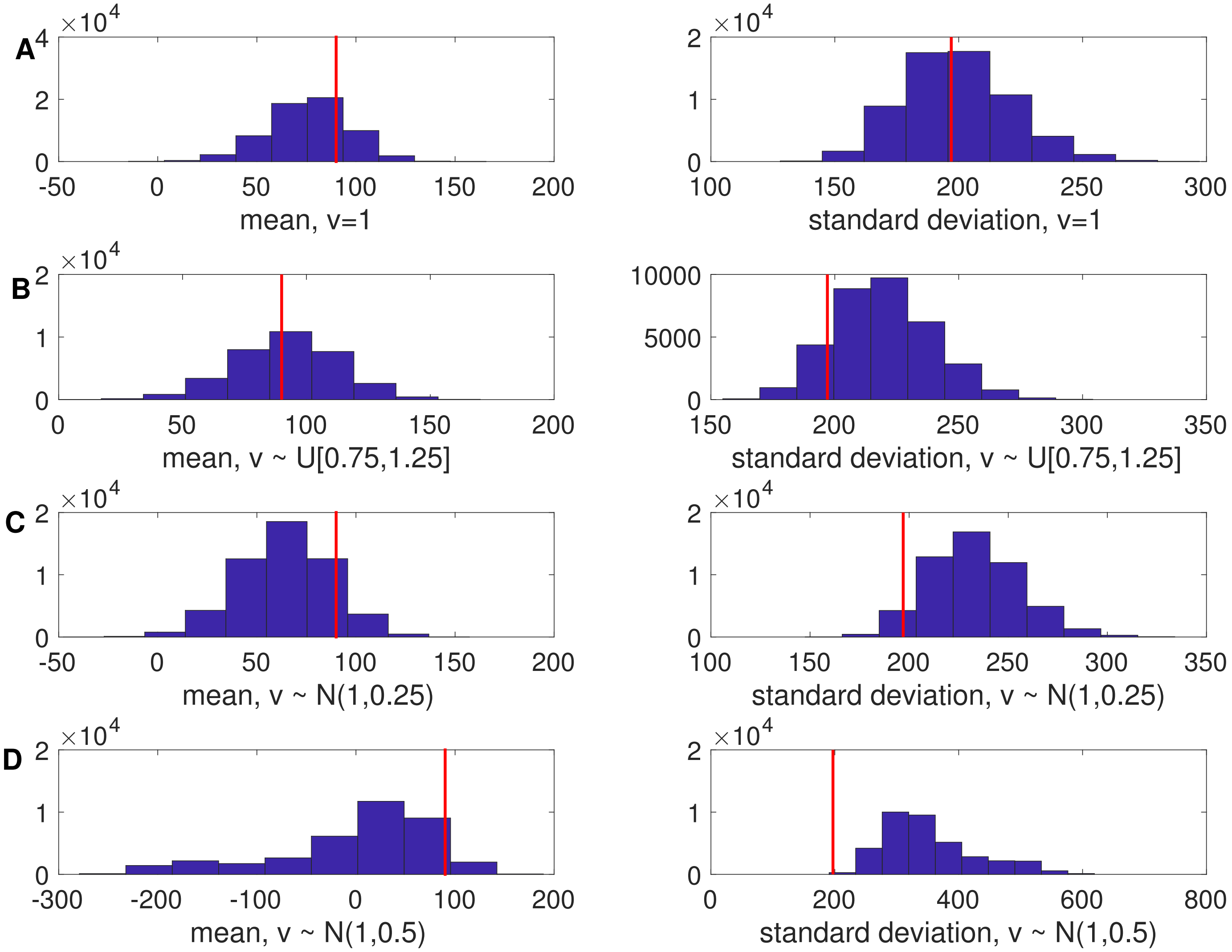

Figure S16 Simulation studies for $t_1 \sim N(600,100)$, $t_2 - t_1 \sim N(90,197)$, $t_3 - t_2 \sim N(-240,297)$, $q_1 = 0.75$, $q_2 = q_1 = 1$, $b = 0.19$, $\tau = 2.1$. **A**: Histograms of mean (left panel) and standard deviation (right panel) of $t_2 - t_1$ with constant fork velocity $v_1 = v_2 = v_3 = 1$. **B**: Histograms of mean (left panel) and standard deviation (right panel) of $t_2 - t_1$ with uniformly distributed fork velocities $v_i \sim U[0.75, 1.25]$, $i \in \{1,2,3\}$. **C,D**: Histograms of mean (left panel) and standard deviation (right panel) of $t_2 - t_1$ with normally distributed fork velocities $v_i \sim N(1, 0.25)$ (**C**), $v_i \sim N(1, 0.5)$ (**D**), $i \in \{1,2,3\}$. Vertical red line indicates the true value of the mean or standard deviation value.

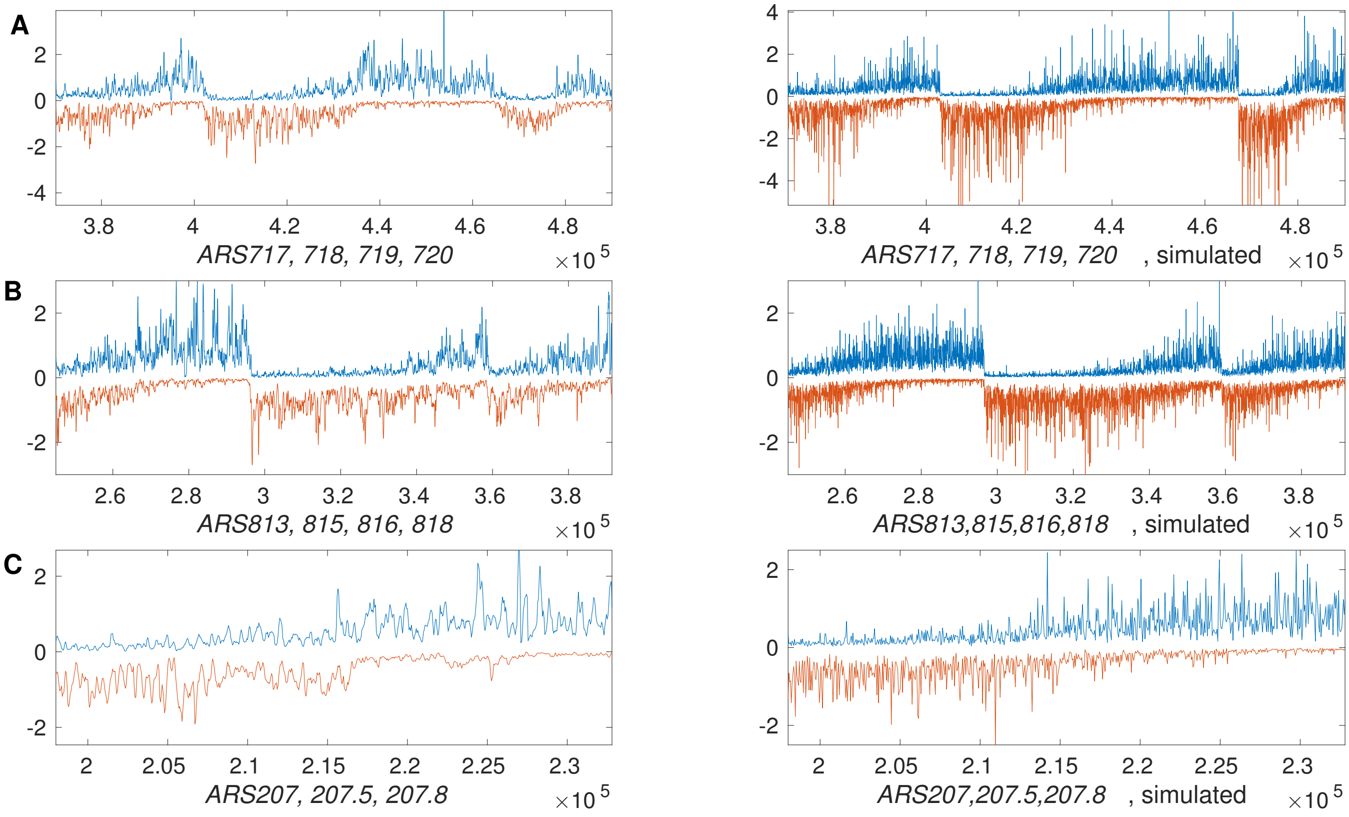

Figure S17 NGS data (left panels) vs simulations (right panels) with the parameters inferred from the RESULTS section, forward (blue) and reverse (red) strands. **A**: chromosome 7 from the section Lack of origin obscuring in strong origin triplets: *ARS717-20*. **B**: chromosome 8 from the sectionTriplets with higher obscuring rates: *ARS813-18*. **C**: chromosome 2 from the section Early, poorly licenced origin: *ARS207.5*, *ARS207.8*, *ARS208*.
