# Supplementary material for: Bayesian inference of origin firing time distributions, origin interference and licencing probabilities from Next Generation Sequencing data
Source: Nucleic Acids Res. 2019 Feb 14;47(5):2229–43. doi: 10.1093/nar/gkz094 (PMC6412128; doi:10.1093/nar/gkz094)
Supplement: Supplementary Data [file gkz094_supplemental_files.zip › BazarovaSuppData.pdf]

Supplementary Data S1: Bayesian inference of origin firing time distributions, origin interference and licensing probabilities from NGS data.
Authors: Alina Bazarova, Conrad Nieduszynski, Ildem Akerman, Nigel Burroughs.
Correspondence: a.bazarova@bham.ac.uk

S1 Supplement

S1.1 Origin Firing model

Consider a set of origins $\{O_i\}$ (numbered consecutively from left to right), with positions x_i and licensing (firing) probability q_i with a firing time distribution $t_i \sim N(\mu_i, \sigma_i^2)$ when licensed. Our model is on the lattice, thus for 3 origins we have O_1 located at 0, O_2 is located at N_1 and O_3 at $N_1 + N_2$. We consider initially the Okazaki fragment (OF) density profile F of a single duplicated genome, so F is piece-wise constant, see Figures 2A,B, S1. Define the *licensed set* S of origins that are licensed to fire during S-phase. Forks progress bidirectionally from each origin at speed v, OFs being laid out complementary to the lagging 5' to 3' strand (DNA synthesis is 5' to 3'). Some origins will be obscured if the fork from a neighbouring origin reaches that origin before it can fire. Thus, the probability of leaving an origin signature in the OF profile is $\tilde{q}_i = q_i \times P(\textit{origin is not obscured}|\textit{licensed})$. Consider the OFs of strand 5' to 3' lying left to right. Between two origins O_i, O_{i+1}, assuming both fire, the forks collide at t^* given by

$$t^* = \frac{1}{2}\left(\frac{(x_{i+1} - x_i)}{v} + t_i + t_{i+1}\right)$$

Thus, the OFs lie in the interval $[x^*_{i+1}, x_{i+1})$, $x^*_{i+1} = x_{i+1} - v(t^* - t_{i+1})$. Collision points have to be integers whilst firing times are continuous; thus we round down to the nearest integer. When O_1 and O_2 forks meet their collision point is $x^*_2 \in \{0, 1, \ldots, N_1 - 1\}$. Similarly for O_2 and O_3, $x^*_3 \in \{N_1, N_1 + 1, \ldots, N_1 + N_2 - 1\}$. Collision points are therefore only dependent on differences in firing times. Obscuring generates collision points outside these intervals. Specifically we require $x^*_{i+1} > x_i$, otherwise O_i is obscured from the fork coming from the right, and $x_i - x_{i-1} < v(t_i - t_{i-1})$ or the origin is obscured by the fork on the left. When licensing is included, these conditions generalise, $x_{lnb(i)} < x^*_i < x_{rnb(i)}$, where $lnb(i), rnb(i)$ are the left/right realised neighbours of i, ie forks of i meet those of $lnb(i)$ and $rnb(i)$ between the respective origins. This profile is a piece-wise constant function,

$$F_j = f_2 I\{x_{21} < N_1\} I\{x_{32} \geq N_1\} \left(I\{j \geq f_1 x_{21}, j < N_1\} + I\{j \geq f_3 x_{32} + (1 - f_3)(N_2 + N_1)\}\right) + \left(1 - f_2 I\{x_{21} < N_1\} I\{x_{32} \geq N_1\}\right) I\{j \geq x_{31}\} \quad (1)$$

at position $j \in \{0, 1, \ldots N_1 + N_2 - 1\}$, where f_2 is an indicator of whether the second origin was licensed, x_{21}, x_{32}, x_{31} are the collision points of the fork between first and second, second and third and first and third origins respectively.

The observed data corresponds to the population average of genome replications in individual cells,

$$F^{ave}(\{q_i, \mu_i, \sigma_i\}) = \sum_S \pi(S) \int \left(\prod_{i \in S} dt_i \pi(t_i | q_i, \mu_i, \sigma_i)\right) F(S, \{t_i, x_i\}_S) \quad (2)$$

where the licensing set S has probability $\pi(S) = \prod_{i \notin S}(1 - q_i) \prod_{i \in S} q_i$, and $F(S, \{t_i, x_i\}_S)$ is the profile generated from licensing set S and firing times t_i of origin $i \in S$. Obscuring reduces the origins that actually fire; define the realised firing set $\{S^*, \{t_i, x_i\}_{i \in S^*}\}$ (unobscured origins) giving $F(S, \{t_i, x_i\}_S) \equiv F(S^*, \{t_i, x_i\}_{S^*})$ as unrealised firing times do not affect the profile.

If origin O_i fails to fire (due to being obscured or not being licensed) its position can be overrun either by the fork coming from O_{i+1} or by the fork coming from O_{i-1}. For the case of an end origin it can be overrun only from one side. For a single profile (for one replicated cell), the profile for the case of O_i being overrun from the right would have a value 1 on the interval $[x_i, x_{i+1})$ ($[0, N_1)$ for $i = 1$, $[N_1, N_1 + N_2)$ for $i = 2$), Fig. 2B of main text. In case of being overrun from the left it would have a value 0 on the interval $[x_{i-1}, x_i)$ ($[0, N_1)$ for $i = 2$, $[N_1, N_1 + N_2)$ for $i = 3$). Thus, the shape of the averaged out profile will be dependent on how often origin O_i fires.

S1.1.1 Profile patterns

Key features in the OF profiles relate to obscuring and licensing events. Firstly, the intercepts at each origin are a direct measure of the frequency, f_l, f_r, with which forks from the left and right respectively overrun that origin (irregardless of whether this is through obscuring or failure of the origin to be licensed), *i.e.* the intercepts on the origin's immediate left, right are $1 - f_l, f_r$. Specifically, if the fork at O_i failed to fire $a\%$ of the time the value of the profile at x_i will be a provided that in all the cases it was overrun by the left-moving fork. On the other hand, cases when O_i is being overrun from the left decrease the value of the averaged out profile at $x_i - 1$ ($N_1 - 1$ for $i = 2$, $N_1 + N_2 - 1$ for $i = 3$), *i.e.* if the fork at O_i failed to fire $d\%$ of the time due to being overrun by a right moving fork the value of the averaged out profile at $x_i - 1$ will be $1 - d$. Therefore by looking at the value of the profile at 0 ($N_1 + N_2 - 1$) we can deduce how often origin O_1 (O_3) failed to fire due to being overrun from the right (left). Origin O_2 can be overrun both from the left and from the right. Given that this is indicated by the values at $N_1 - 1$ and N_1 respectively we can also compute how often this origin fired by subtracting the value of the profile at N_1 from the value of the profile at $N_1 - 1$. Examples are shown on Figs. 2, S2.

Secondly, if the gradients at the origin are flat this indicates that obscuring is not occuring since the fork collision/termination sites are distant from the origin. Our assumption that firing times are Gaussian is important here, as it means that the termination distribution is Gaussian, *i.e.* 'rogue' terminations away from the bulk of the distribution are not present. Thus partial origin obscuring gives rise to sharp profile gradients at the origins. Complete (100%) obscuring is equivalent to a failure to license. For example, in Fig. S3A origin O_2 is obscured 70% of the time by the fork from O_3 on the right; the profile intercept at O_2 (immediate right) is 70% and has a non-zero gradient since fork collision points are occuring right up to the origin. This constrasts to the case when O_2 is licensed 30% of the time and forks collide at a distance; the profile is then near flat close to O_2, Fig. S3B. Similar, but inverted profiles occur to the left of an origin, Fig. S3C/D. Obscuring equally from both neighbouring origins is shown in Fig. S3E, where both sides have high gradients.

Thirdly, distortions of profile shape away from the tanh-like profile of Fig. 2C (main text) occur as fork collisions from multiple origin pairs occur, a consequence of partial licensing of intermediate origins, Fig. S3B/D/F. Obscuring coupled with partial licensing can generate complex profiles, Fig. S3F: the O_1O_3 fork collisions give rise to the sharp transitions at O_2, whilst O_2O_1, O_2O_3 collisions (occuring 30% of the time) result in bumps in the profiles away from O_2 giving one possible signature for partial licensing. Finally, the sharpness of the profile is determined by the standard deviation of the distribution of the difference in the firing times of the two origins that generated the colliding

forks.

S1.2 Likelihood

We assume a Log-normal error model for OF coverage of each site with a background contamination of random fragmentation,

$$\begin{aligned} X_j^f &= ((1-b)F_j^{ave} + 0.5b)e^{N(-0.5\tau^{-1},\tau^{-1})} \\ X_j^r &= ((1-b)(1-F_j^{ave}) + 0.5b)e^{N(-0.5\tau^{-1},\tau^{-1})} \end{aligned} \quad (3)$$

where X_j^f are the (normalised) read counts on the forward (3' to 5') strand and X_j^r - on the reverse strand for position j. Other noise models could be used; the key requirements are $X_j^{r/f} > 0$ and noise is not additive. The data suggests that a log-normal is appropriate, Fig. S4, S5.

Equations (3) assume the same levels of noise for both strands. However the model can be easily extended to the case when they are subject to noise with different characteristics. We introduce parameters τ_1 and τ_2 for log-normal distributions and a parameter α for background noise b. We obtain the following normalised counts at genome position j,

$$\begin{aligned} X_j^f &= ((1-b)F_j^{ave} + \alpha b)e^{N(-0.5\tau_1^{-1},\tau_1^{-1})} \\ X_j^r &= ((1-b)(1-F_j^{ave}) + (1-\alpha)b)e^{N(-0.5\tau_2^{-1},\tau_2^{-1})}, \end{aligned} \quad (4)$$

All these models satisfy the normalisation condition $\mathbf{E}\left[X_j^f + X_j^r\right] = 1$. Although there was evidence that noise on the two strands was different, with the forward strand being noiser (*ARS210-212*), other aspects of the model were invariant, section S1.9, Supplementary Fig. S6. Thus, we only present results for model (3).

We would like to compute the posterior $\pi(\{q_i,\mu_i,\sigma_i\}, b, \tau | X_j, \{x_i\}, v)$. However, expression (2) involves high dimensional integrals and a sum over licensed sets S so the likelihood is intractable. The simplest approach is to approximate (2) with a sample,

$$F^{ave}(\{q_i,\mu_i,\sigma_i\}) = \frac{1}{M}\sum_k F(S_k, \{t_i, x_i\}_{S_k})$$

where the sum is over M independent samples from the distribution $\pi(S)\prod_i \pi(t_i|q_i,\mu_i,\sigma_i)$. This gives the log-Normal model at position j,

$$\log X_j^f = \log\left((1-b)\frac{1}{M}\sum_k F_j^k + \frac{1}{2}b\right) + N(-0.5\tau^{-1},\tau^{-1}), \quad (5)$$

F^k being the kth sample, $F^k = F(S_k, \{t_i, x_i\}_{S_k})$. The posterior is then given by,

$$\pi(\{q_i, \mu_i, \sigma_i\}, b, \tau | X_j^f, X_j^r) \propto \pi(b)\pi(\tau) \prod_i \pi(\mu_i)\pi(\sigma_i^{-2})\pi(q_i)$$

$$\sum_k \pi(S_k|q) \int \prod_i dt_i \pi(t_i^k|q_i, \mu_i, \sigma_i) \prod_j \left[\tau \exp -\frac{1}{2}\tau \left(\log X_j^f - \log((1-b)\frac{1}{M}\sum_k F_j^k + 0.5b) + \frac{\tau^{-1}}{2}\right)^2 \times \right.$$

$$\left. \exp -\frac{1}{2}\tau \left(\log X_j^f - \log((1-b)\frac{1}{M}\sum_k F_j^k + 0.5b) + \frac{\tau^{-1}}{2}\right)^2 \right] \quad (6)$$

where the prefactors are independent priors, and t_i^k are the firing times for origin i in the kth realisation F^k.

Each F^k requires the sampling of which origins are licensed (set S_k) and then sampling the firing times. It is easiest to introduce indicator variables f_i^k to indicate if origin i is licensed, $\pi(f_i^k = 1) = q_i$, then $S_k = \{i | f_i^k = 1\}$. We obtain the augmented posterior,

$$\pi(\{f_i, t_i\}, \{q_i, \mu_i, \sigma_i\}, b, \tau \quad | \quad X_j^f, X_j^r) \propto \pi(b)\pi(\tau) \prod_i \pi(\mu_i)\pi(\sigma_i^{-2})\pi(q_i)$$

$$\prod_j \tau \exp\left(-\frac{\tau(\left(\log X_j^f - \log((1-b)\frac{1}{M}\sum_k F_j^k + 0.5b) + \frac{\tau^{-1}}{2}\right)^2}{2}\right) \quad (7)$$

$$\prod_j \exp\left(-\frac{\tau(\left(\log X_j^r - \log((1-b)(1-\frac{1}{M}\sum_k F_j^k) + 0.5b) + \frac{\tau^{-1}}{2}\right)^2}{2}\right) \quad (8)$$

$$\prod_i \sigma_i^{-M} \exp\left(-\sum_k \frac{\left(t_i^k - \mu_i\right)^2}{2\sigma_i^2}\right) \prod_k \prod_{i|f_i^k=1} q_i \prod_{i|f_i^k=0} (1-q_i) \quad (9)$$

Here we have used a Gaussian pseudo-prior for the firing times for the origins that are unlicenced, ie their firing time is not present in the sampler. We set the pseudo-prior equal to the firing time distribution $N(\mu_i, \sigma_i^2)$, physical and non-physical firing times are then not distinguished in the above simplifying the expression.

The likelihood can be modified to allow for missing data; specifically allowing for unsequencable regions which are simply left out of the likelihood, (7), (8). The associated MCMC algorithm follows.

Priors. We use uninformative priors. We use a Gaussian zero-mean prior on parameter b with precision $\tau_b = 10^{-7}$ truncated to $(0, 1)$ and a Gamma prior on noise parameter τ with shape parameter $\alpha_\tau = 0.01$ and rate parameter $\beta_\tau = 0.01$. Priors on $\{\mu_i\}$ are also zero-mean Gaussian with precisions $\{\tau_i\} = 10^{-8}$. Priors on $\{\sigma_i^{-2}\}$ are Gamma with shape parameters $\{\alpha_i\}$ equal to 3 and large rate parameters $\{\beta_i\} = 6 \times 10^4$. Finally, priors on q_i are uniform on $[0, 1]$.

Time identifiability. There is a time symmetry allowing the origin of time to be shifted arbitrarily; thus the $\text{mean}_{i \in S}(t_i)$ will diffuse and only time differences should be analysed. We fix the time symmetry by setting the sum $\sum_k \left(t_1^k + t_2^k + t_3^k\right) = 0$; other options are possible.

Profile identifiability. At extreme time differences there is the possibility that F^k is flat with an end fork obscuring all others. This introduces an identifiability issue since such an F is

indistinguishable from a shift in b. Such profiles are prohibited from occuring (termed illegal states in the following).

Model reduction. The hierarchical layer comprising μ_i, σ_i is simple to update; in essence it calculates the mean and variance of t_i which we can easily compute from the samples. Removing this layer, (removing μ_i, σ_i), giving posterior $\pi(\{f_i, t_i\}, \{q_i\}, b, \tau | X_j, \{x_i\}, v)$ as might be anticipated to be appropriate for a more general analysis (to remove the Gaussian firing time assumption) however leads to an improper probability density as the tail t_i is unconstrained (when obscured) unless a proper prior is used for t_i. However, this prior would then affect the frequency of $f_i = 1$, and thus would bias the estimation of the priming probability. Thus, the layer functions to define the firing time t_i as Gaussian, controlling the tail to give the correct licensing probability and crucially, controls the tail of t_i when the origin is obscured.

S1.3 MCMC

The MCMC sampler has Gibbs and Metropolis-Hastings (MH) updates; we update variables separately except for the firing times of an individual profile which are updated together. To sample the posterior (9) the updates are as follows:

Random fragmentation/Signal weight b: A Metropolis-Hastings update with a random walk (RW) proposal, $\Delta b \sim N(0, s_b^2)$, constrained with $b \in [0, 1]$. The step size standard deviation s_b is tuned during the burn-in to give an acceptance rate within the range (0.2, 0.4), [Roberts et al., 1997].

Log-normal noise precision τ: A random walk proposal is used $\Delta\tau \sim N(0, s_\tau^2)$, with constraint $\tau > 0$. The RW standard deviation s_τ is tuned during the burn-in to give an acceptance rate within range (0.2, 0.4).

Origin firing time: mean μ_i: The conditional distribution $\pi(\mu_i|\cdot)$ is Gaussian, so we use a Gibbs update $\mu_i|. \sim N(\frac{1}{M}\sum_k t_i^k, \sigma_i^2/(M + \tau_i\sigma_i^2))$.

Origin firing time: precisions σ_i^{-2}: The conditional distribution $\pi(\sigma_i^{-2}|\cdot)$ is Gamma, thus we use a Gibbs update $\sigma_i^{-2}|. \sim \Gamma(\frac{M}{2} + \alpha_i, \frac{1}{2}\sum_k (t_i^k - \mu_i)^2 + \beta_i)$.

Licensing probability q_i: The conditional distribution $\pi(q_i|\cdot)$ is Beta, so we use a Gibbs update $\sim Beta(\sum_k f_i^k + 1, M - \sum_k f_i^k + 1)$.

Licenced origin flag $\{f_i^k\}$: A Metropolis-Hastings update with a multinomial proposal. We propose a licensing configuration for all 3 origins together, each realisation $k \in \{1, \ldots, M\}$ updated separately. The distribution P of the proposal is computed in a following way

$$P(f_1^k f_2^k f_3^k) = \frac{\pi(f_1^k f_2^k f_3^k)}{Z_f(t_1^k, t_2^k, t_3^k)}$$

where $\pi(f_1^k f_2^k f_3^k) = q_1^{f_1^k} q_2^{f_2^k} q_3^{f_3^k}$ provided state $f_1^k f_2^k f_3^k$ is not an (illegal) flat profile given the firing times, e.g. through obscuring, or licensing. The denominator $Z_f(t_1^k, t_2^k, t_3^k)$ is a normalisation factor. This removes states where two or more consecutive indicators are equal to zero, the middle origin is obscured by one of the end ones and the other end one is inactive, or $|t_3 - t_1| > N_1 + N_2$ and the middle origin is inactive. The MH acceptance ratio then reduces to the ratio of likelihood terms (7),(8) which depend only on the profile F_j^k.

Profile firing times t_i: We use a reversible jump sampler since the number of variables changes depending on obscuring and activation. To minimise correlations, we update separately the sum $\sum_i t_i^k$ and the differences $t_2^k - t_1^k$, $t_2^k - t_3^k$ and $t_3^k - t_1^k$ for each origin separately. Only two of these

differences are independent, the choice of which two differences to sample is dependent on which times are physical and is explained below.

For $\sum_i t_i^k$ a Gaussian move with the distribution $N(mean_k\left(\sum_i t_i^k\right), \text{var}_k(\sum_i t_i^k))$ is used, where $mean_k, var_k$ are taken over the M realisations. The MH acceptance ratio is for proposal y is

$$\frac{\pi(y|t,\sigma,\mu)q(t_1^k+t_2^k+t_3^k|y)}{\pi(t_1^k+t_2^k+t_3^k|t,\sigma,\mu)q(y|t_1^k+t_2^k+t_3^k)},$$

where $\pi(\cdot|t,\sigma,\mu)$ are the relevant terms of likelihood and q is the proposal density. Here, since the likelihood/profile is independent of the sum $t_1^k+t_2^k+t_3^k$, terms (7) and (8) cancel. We update the $\sum_i t_i^k$ for each profile k individually. If the proposed value y is accepted a linear shift by $-(y-t_1^l-t_2^l-t_3^l)/M$ is performed for each of the M realisations of $\sum_i t_i^k$ in order to preserve the constraint $\sum_{k,i} t_i^k = 0$.

To update the time differences for profile k, we construct a reversible jump sampler as follows (for convenience we suppress index k from the notation). We define physically meaningful regions in the time difference space depending on whether any origin is obscured conditional on the values of $\{f_i\}_{i=1}^3$. Cases when two origins are obscured by an end origin are illegal since the resulting flat profile is indistinguishable from a shift in the fragmentation parameter b. For the case when $\sum_i f_i = 3$ we have the following 5 regions (the superscript refers to the origins which are licensed, all 3 in this case) :

$$R_0^{123} = \{t_1,t_2,t_3 : -N_1 < t_2-t_1 < N_1, -N_2 < t_2-t_3 < N_2\}$$
$$R_2^{123} = \{t_1,t_2,t_3 : (t_2-t_1 \geq N_1)||(t_2-t_3 \geq N_2), -N_1-N_2 < t_3-t_1 < N_1+N_2\}$$

$$R_1^{123} = \{t_1,t_2,t_3 : t_1-t_2 \geq N_1, -N_2 < t_3-t_2 < N_2\}$$

$$R_3^{123} = \{t_1,t_2,t_3 : t_3-t_2 \geq N_2, -N_1 < t_1-t_2 < N_1\}$$

$$R_{13}^{123} = \{t_1,t_2,t_3 : t_1-t_2 \geq N_1, t_3-t_2 \geq N_2\}$$

Regions are labelled by superscripts indicating the licensed origins. R_0^{123} corresponds to the case when no obscuring occurs. R_2^{123} corresponds to the middle origin obscured by either one of the end ones. R_i^{123}, $i=1,3$ corresponds to an end origin being obscured by the middle one (and the other one is not). R_{13}^{123} - both end origins are obscured by the middle one.

We associate a system of coordinates with each of the regions. For R_0^{123}, R_i^{123}, R_{13}^{123} it will be $\{t_2-t_1, t_2-t_3\}$, for R_2^{123}, $\{t_3-t_1, t_2-t_1\}$ or $\{t_3-t_1, t_2-t_3\}$ depending on which origin obscures the middle one. This means that whenever we are to make a move into one of the first three regions we update $\{t_2-t_1, t_2-t_3\}$ and derive the remaining time difference from these two. In case of R_2^{123} we update t_3-t_1 and depending on the obtained value we update t_2-t_1 or t_2-t_3.

We propose a jump between these regions using an equiprobable distribution, *i.e.* choose a region with probability 1/5 (including the current region). Then within each region we draw the appropriate time differences using truncated Gaussian distributions. As a mean and standard deviation of the Gaussian distribution we use the mean and standard deviation over all the active realisations of the corresponding firing time differences, *i.e.* the difference $t_i^h - t_j^h$ for profile h is included into the computation of the proposal if and only if $f_i^h = f_j^h = 1$ and therefore also

includes obscuring events when one of the times may not be realised. We update each of the M profiles individually, which means that for each $k = 1, \dots M$ we sample for region, and then for that region draw the required time differences. Note that the mean and variance of the Gaussian distribution for the time difference does not depend on the region we are moving into, only the truncation points vary. For example to jump into the region R_0 we draw $t_2 - t_1$ and $t_2 - t_3$ from a truncated (between $-N_1$ and N_1, $-N_2$ and N_2 respectively) Gaussian distribution with corresponding mean and variance. In case of R_2 we will draw $t_3 - t_1$ from truncated Gaussian on the interval $(-N_1 + N_2, N_1 + N_2)$. Then if $t_3 - t_1$ is larger than $N_1 - N_2$ we draw $t_2 - t_1$ from the truncated Gaussian distribution on $[N_1, \infty)$, otherwise we draw $t_2 - t_3$ from the distribution with the same mean and variance but on $[N_2, \infty)$.

If one of the origins is not licensed the set of proposals changes depending on which time differences are physical. For example if the middle origin was not licensed the only physical quantity will be $t_3 - t_1$. In this case there are no constraints on the values of $t_2 - t_1$ and $t_2 - t_3$ and hence we do not have to sample them from a truncated distribution. The only constraints are that the illegal states leading to a flat profile are not allowed. The sampler works in a same way as before. Two values are generated: one for $t_3 - t_1$, since it is the physical difference and another one either for $t_2 - t_1$ or for $t_2 - t_3$ is chosen depending on which difference has more realisations.

$$R^{13} = \{t_1, t_2, t_3 : -\infty < t_i - t_2 < \infty, -N_1 - N_2 < t_3 - t_1 < N_1 + N_2,\ i = 1 \text{ } or \text{ } 3\}$$

If the middle origin is the only licensed origin (the two others are not) we propose values for $t_2 - t_1$ and $t_2 - t_3$. Again we do not set any constraints on the proposal besides the profile identifiability constraint to prevent flat profiles occuring. This corresponds to the region

$$R^2 = \{t_1, t_2, t_3 : -\infty < t_1 - t_2 < \infty, -\infty < t_3 - t_2 < \infty\}$$

The remaining two states correspond to the cases when one of the end origins is not licensed. Only the (licensed) end origin can be obscured; obscuring of the middle origin is illegal. We can define two regions for licensing of origins 1 and 2,

$$R_0^{12} = \{t_1, t_2, t_3 : -N_1 < t_1 - t_2 < N_1, f_3 = 0\}$$

$$R_0^{23} = \{t_1, t_2, t_3 : -N_2 < t_3 - t_2 < N_2, f_1 = 0\}$$

$$R_1^{12} = \{t_1, t_2, t_3 : t_1 - t_2 \geq N_1,\ f_3 = 0\}$$

$$R_3^{23} = \{t_1, t_2, t_3 : t_3 - t_2 \geq N_2,\ f_1 = 0\}$$

The principle is the same as before. We randomly choose which region to move into with equiprobable weights and then generate the appropriate time differences, $t_2 - t_j$ from a non-truncated Gaussian distribution since O_j, $j = 1, 3$ is not licensed and another one for $t_2 - t_i$, $i \neq j$ from a truncated Gaussian depending on whether the target region includes obscuring events. Truncation is done in a similar way to the case when all origins are licensed.

With the proposals constructed as above, we perform a Metropolis-Hastings acceptance/rejection step in a reversible jump sampler framework ([Hastie and Green, 2012]); specifically we work in a

2D space with the likelihood being dependent on 0,1 or 2 physical dimensions. This means that the acceptance probability of the state x' at a state x, $\alpha(x, x')$ is

$$\alpha(x, x') = \min\left\{1, \frac{\pi(x')j_m(x')g'_m(x', u')}{\pi(x)j_m(x)g_m(x, u)}\left|\frac{\partial(x', u')}{\partial(x, u)}\right|\right\} \tag{10}$$

where π is a likelihood, g_m is a density of the proposal to move into the mth region, g'_m is a density of the reverse proposal, $\left|\frac{\partial(x',u')}{\partial(x,u)}\right|$ is a Jacobian of the transformation from current coordinate system of (x, u) to coordinate system (x', u') (here x are the physical dimensions, u being the supplementary dimensions used to facilitate the jump sampler and keep the combined dimension constant).

Our sampler works as follows. Let x denote the physical firing time differences, and u unrealised (because of obscuring) time differences. x is 2-dimensional in the case of R_0^{123}, 1-dimensional in case of R_i^{123}, $i = 1, 2, 3$, R_0^{23}, R_0^{21}, R^{13} and null in the case of R^2, R_{13}^{123}, R_1^{12}, R_3^{23}. Generate values (x', u') in accordance with the coordinate system of the new region we are proposing to move to. If we are trying to move into the region R_0^{123} with two dimensions, u' is null since both firing time differences are realised. Similarly, for the one-dimensional region (R_i^{123}, $i = 1, 2, 3$, R_0^{23}, R_0^{21}, R^{13}) or a region corresponding to a single fork profile (R^2, R_{13}^{123}, R_1^{12}, R_3^{23}) u' will either have one or two dimensions respectively. The coordinate system of each region is defined via two of the firing time differences $\{t_2 - t_1, t_2 - t_3, t_3 - t_1\}$. The Jacobian of the transformation from any one system of coordinates to any other is therefore trivially equals to 1. Given that we switch between regions uniformly at random the j_m terms cancel out and hence expression (10) simplifies to

$$\alpha(x, x') = \min\left\{1, \frac{\pi(x')g'_m(x', u')}{\pi(x)g_m(x, u)}\right\}$$

S1.4 Improving algorithm efficacy

The mixing and convergence of the above algorithm can be improved by two strategies. Firstly, the weights with which the different configurations R_i^s are proposed can be updated at each step according to the current state of the chain using the frequencies of the states R_i^s (fixed licensing s) amongst the M profiles. We used proposal weights $Z^{-1}\max(\epsilon, n_i/M)$, with n_i the number of profiles out of M in R_i^s, Z a normalising factor, thereby always retaining a probability ϵ/Z, $\epsilon = 0.02$ of proposing any unobserved configurations. Secondly, we observed that convergence times increased with M. Thus, we modified the algorithm to use low M initially, $M = 312$, which we doubled 4 times during burn-in to reach $M = 4992$. Doubling involved duplicating all profiles. There were 50000 steps before the first doubling, 25000 between the first and the second doubling, 12500 between the second and the third and finally 6250 steps between the third and the fourth one. This was sufficient for duplicated profiles to become as divergent as nonduplicated profiles since no statistically significant differences were detected between them.

S1.5 Convergence diagnostics

We used the Gelman-Rubin multiple chain convergence diagnostic [Gelman and Rubin, 1992], with 6 multiple chains initialised as below. We used a threshold of 1.05 on the dimension corrected GR statistic on each of the global variables b, τ, μ_i, σ_i, and the summaries $\sum_j t_j, var(t_j)$.

For data shown, Markov chain initialisation was partially random using randomised firing times: $t_2^k - t_3^k$ and $t_3^k - t_1^k$ were initialised from uniform distributions $U([-3N_2, 3N_2])$ and $U([-N_2 - N_1, N_1 +$

N_2]) respectively and $t_2^k - t_1^k$ was derived from these, for $k = 1 \dots M$. Other variables were fixed: $b = 5{\times}10^{-6}$, $\tau = 10^{-6}$, $\mu_i = 0$, $\sigma_i = 5{\times}10^5$, $q_i = 1$, $f_i^k = 1$, $t_1^k{+}t_2^k{+}t_3^k = 0$ for $i = 1, 2, 3$, $k = 1 \dots M$. We also compared to fully random initial conditions on all the data sets as follows. Firing times were initialised as bove, parameters b, τ, μ_i, σ_i, q_i were initialised from their prior distributions (specified in S1.2), f_i^k were initialised using q_i and the identifiability constraints analogous to the proposal for fs (S1.3), $i = 1, \dots, 3$, $k = 1, \dots, M$. Convergence on these chains took marginally longer (figure S7), but converged to the same states. Those chains which did not converge within 300,000 iterations converged after being relaunched with a $\times 2$ longer burn-in and doubled ϵ (see section S1.4).

S1.6 Simulation studies

In this section we demonstrate that our inference algorithm accurately estimates the model parameters on simulated data. We include an example with no obscuring and all origins being licensed all the time (figure S8), an end origin with partial licensing (figure S9), middle origin being partially licensed (figure S10), obscuring of one of the end origins (figure S11), obscuring of the middle one (figure S12) and a profile with both obscuring and partial licensing (figure S13). In all these cases inference is based on MCMC runs with burnin 50000 and 150000 post-burnin with $M = 200$, $N_1 = N_2 = 1000$.

S1.7 Priors on firing times

As indicated in section S1.3 we assume Gaussian prior on the origin firing times. As previously shown in [Retkute et al., 2012] approximation of the profiles by using different probability distributions leads to similar values of mean and standard deviations.

To confirm that Gaussian priors on the firing time distribution is a fair assumption we ran our algorithm on a triple *ARS1018*, *ARS1019*, *ARS1021* with a very broad Gaussian prior with pre-specified mean of 0, and standard deviation of around 220 krbp (cf figure S14). Nothing can be said about the firing time distribution beyond the point of obscuring since we simply do not possess the data on when it would fire if it were not obscured, and therefore the distribution there will inevitably follow the distribution of the prior.

The absense of a strong prior also leads to a drop in the convergence rate so if the initial firing times are sampled from a broad enough interval there will be several ones out of M realisations sitting in the obscuring zone for a while.

After getting rid of the latter ones we can compare the firing time distributions provided by the algorithm with a strong Gaussian prior with no pre-specified parameters and the ones with a broad prior and see that although the first inference produces a much smoother curve the general trends are the same.

S1.8 Noise analysis

We chose a model with multiplicative log-Normal noise since it fits the Okazaki fragment sequencing data ([Smith and Whitehouse, 2012], [Petryk et al., 2016]) well and does not require many additional parameters to be included in the model.

Indeed, assume that the noise is additive and for simplicity normally distributed. Then the data X_j at any point on the chromosome j can be written as $X_j = a_j + N(\mu, \sigma)$, where a_j is the value of the signal at a point j, μ and σ are noise parameters. Then the standard deviation of X_j, σ will

stay constant for all j. Figure S4B,C is a direct contradiction to this statement since it shows that when moving average (a signal approximation) is subtracted from the data the standard deviation of the noise is proportional to it.

On the other hand, a log-Normal model with multiplicative log-Normal noise can be written as $X_j = a_j \exp\{N(\mu, \sigma)\}$ using the same notation. Therefore $X_j \sim \exp\{N(\log(a_j) + \mu, \sigma)\}$, which in turn means that the standard deviation of X_j in this case will be linearly proportional to the value of a_j, *i.e.* it will be equal to $a_j\sqrt{\sigma^2 - 1}$. This is what the figure S5A shows for both strands. We note that there is a number of outliers present both on the forward and reverse strands, but their overall amount is no more than 5% of all data points. The approximation of $\log(X_j/a_j)$ (where X_j/a_j is read counts divided by its moving average) by a normal distribution which is demonstrated on figure S5B does not exhibit significant discrepancy, which therefore supports the assumption of the log-Normality. Note that the exponent of the approximating distribution $N(-0.09, 0.5)$ has a mean of 1.03 which is close to 1. Moreover, the slope of the least squares lines (yellow line) on the figures S5A in both cases is around 0.5, which is in agreement with the approximating distribution, where $\sqrt{\sigma^2 - 1}$ is approximately 0.53.

We also performed analysis for the sum of the read counts from both strands. For each of the chromosomes we computed the strand bias according to formula (**2**) and analysed the series $\{c_i^f + b_{chr}c_i^r\}_i$ (in the notation of (**2**)). The log-transform of these series is well approximated by a normal distribution (figure S4). This again confirms the validity of a multiplicative log-normal noise model (3). Note that the data has not been boxed at this point.

S1.9 Different noise levels on the two strands

To analyse if there is strand specific noise, we examined the inference of the generalised model (4) on experimental data. Specifically, we fitted three models: α a free parameter and $\tau = \tau_1 = \tau_2$, with α fixed to 0.5 and different τ_1 and τ_2, and with the 3 free parameters α, τ_1 and τ_2. We illustrate on three consecutive origins *ARS718-20* and plotted the inferred firing time differences on figure S6. Although according to figure S6A,B there is a difference in noise levels between two strands, Figure S6C does not suggest a significant difference in the inferred firing times.

S1.10 Effect of M

As M increases the inferred profile becomes smoother and the inferred firing time histograms become smoother. This suggests that at lower M noise is being over-interpreted. This is illustrated on the time difference distributions for two origin triples, Figure S15 for various M. The distributions for $M = 4992$ are much smoother than those for lower M although there is no significant difference in the mean and standard deviation of the distribution with increase in M. For each of the examples in figure S15 we used a 2 sample F-test and t-test on 1000 samples from each pair of distributions corresponding with different values of M to decide whether there is a statistically significant difference between the mean values and the standard deviations for different Ms. In the case of chromosome 7, the lowest p-values were 0.0875 for the F-test and 0.098 for the t-test (corresponding to pair $M = 312$ and $M = 2496$) suggesting that there is no statistically significant difference between these distribution moments. The largest difference between the means and standard deviations were 13% and 9% for the pair $M = 312$ and $M = 2496$ respectively. For chromosome 8, the lowest p-value was 0.1373 for F-test and 0.0589 for t-test (corresponding to the pair $M = 624$ and $M = 2496$) and the maximal difference between the mean values and standard

deviations was 11% and 7% for pairs $M = 312$, $M = 4992$ and $M = 624$, $M = 2496$ respectively. This indicates that posterior distribution moments are invariant to M, justifying the increase in M utilised in the MCMC algorithm to improve its convergence, section S1.4.

S1.11 Fork velocity variability

In this section we demonstrate that our algorithm is robust towards variable fork velocity. We simulate four data-sets with same firing time and licensing parameters close to the ones inferred from the left triple chromosome 7 example (*ARS717-719*) adjusted to 50 bp boxing: $\bar{t}_2 - \bar{t}_1 = 90$, $\bar{t}_3 - \bar{t}_2 = -240$, $sd(t_2 - t_1) = 197$, $sd(t_3 - t_2) = 297$, $q_1 = 0.75$, $q_2 = q_3 = 1$ and $M = 312$. First data-set we simulated with constant fork velocity $v = 1$. Further we assumed that all four replication forks observed in the given origin triplet move with speeds coming from the same distribution and both forks emanating from the middle origin have the same speed. Therefore we introduced three additional M-dimensional parameters v_i, $i \in \{1, 2, 3\}$. Second data-set we generated with the use of Uniform distribution, $1/v_i \sim U[0.75, 1.25]$, third and fourth data-sets we generated with normally distributed fork velocities $v_i \sim N(1, 0.25^2)$ and $v_i \sim N(1, 0.5^2)$ truncated to the interval $(0, 2)$ respectively. The accuracy of firing time inference for each of the data-sets is shown on figure S16.

S1.12 Firing time standard deviation trends

We analysed the standard deviations of the firing times for trends in the separation distance between the origins. We used all the paired end data sets described in the main text, and two additional triples from [Smith and Whitehouse, 2012]. Overall we have data from 4 chromosomes (5,7,8,10) divided into 4 sets (2 quadruples, one quintuple and one region of 16 origins). We plot the standard deviations of firing times from these datasets against the distances between each pair of consecutive origins (Figure 12 of main text).

A correlation analysis of the standard deviation vs distances between the origins shows no correlation, $p-$value=0.13, correlation coefficient 0.29. Figure 12 suggests that standard deviations of firing times are more related for origin pairs from the same chromosome. To test this we performed a permutation test using the ratio of between-group variance to the total variance as a test statistic. On 10000 random permutations over the distances between origins, we obtained a $p-$value < 0.0001 thereby confirming heterogeneity.

S1.13 Median replication times T_{rep}

To determine the median replication times for the regions between the origins we proceed as follows: we assigned the earliest (median) inferred firing time the value zero and computed the values for T_{rep} in this case. We used fork velocity given in [Hawkins et al., 2013], v=1.6 kb/min and boxed the data to 1kb partitions (boxing our analysis by 20 as performed on a 50bp partition) to match the format of the data from [Müller et al., 2014]. Then we added to the profile the median replication time in the corresponding region observed in [Müller et al., 2014].

References

[Gelman and Rubin, 1992] Gelman, A. and Rubin, D. B. (1992). Inference from iterative simulation using multiple sequences. *Statist. Sci.*, 7(4):457–472.

[Hastie and Green, 2012] Hastie, D. I. and Green, P. J. (2012). Model choice using reversible jump markov chain monte carlo. *Statistica Neerlandica*, 66(3):309–338.

[Hawkins et al., 2013] Hawkins, M., Retkute, R., Müller, C. A., Saner, N., Tanaka, T. U., de Moura, A. P. S., and Nieduszynski, C. A. (2013). High-resolution replication profiles define the stochastic nature of genome replication initiation and termination. *Cell Reports*, 5(4):1132–1141.

[McGuffee et al., 2013] McGuffee, S. R., Smith, D. J., and Whitehouse, I. (2013). Quantitative, Genome-Wide Analysis of Eukaryotic Replication Initiation and Termination. *Molecular Cell*, 50(1):123–135.

[Müller et al., 2014] Müller, C. A., Hawkins, M., Retkute, R., Malla, S., Wilson, R., Blythe, M. J., Nakato, R., Komata, M., Shirahige, K., de Moura, A. P., and Nieduszynski, C. A. (2014). The dynamics of genome replication using deep sequencing. *Nucleic Acids Research*, 42(1):e3.

[Petryk et al., 2016] Petryk, N., Kahli, M., d'Aubenton Carafa, Y., Jaszczyszyn, Y., Shen, Y., Silvain, M., Thermes, C., Chen, C.-L., and Hyrien, O. (2016). Replication landscape of the human genome. *Nature Communications*, 7:10208 EP –. Article.

[Retkute et al., 2012] Retkute, R., Nieduszynski, C. A., and de Moura, A. (2012). Mathematical modeling of genome replication. *Phys. Rev. E*, 86:031916.

[Roberts et al., 1997] Roberts, G. O., Gelman, A., and Gilks, W. R. (1997). Weak convergence and optimal scaling of random walk metropolis algorithms. *Ann. Appl. Probab.*, 7(1):110–120.

[Smith and Whitehouse, 2012] Smith, D. J. and Whitehouse, I. (2012). Intrinsic coupling of lagging-strand synthesis to chromatin assembly. *Nature*, 483(7390):434–438.

S2 Supplementary tables

	O_1	O_2	O_3	O_4	O_1O_2	O_2O_3	O_3O_4
mean μ	4427	-20539, -21685	16052, 10819	10867	-25021	36591,32504	49
sd μ	499	554, 871	557,1482	789	837	921, 2267	2194
mean σ	6396	24188, 15664	12731, 24056	6385	25067	27418,28769	24924
sd σ	1474	906, 1463	2171, 1981	1207	781	951,1585	1911
mean *obsc*	0.14*	0	0.17*,0.17*	0.09*			
sd *obsc*	0.01*	0	0.01*,0.04*	0.01*			
mean q	0.99	0.97,1	0.99,0.9	1			
sd q	0.01	0.01,0	0.01,0.04	0			
mean $\pi(t_{i+1} < t_i)$					0.84	0.09,0.13	0.5
sd $\pi(t_{i+1} < t_i)$					0.01	0.01	0.04

Table S1 **Inferred origin characteristics** ***ARS813-18*****.** Posterior mean and standard deviations of the firing time parameters μ_i and σ_i for origin O_i, and their differences between pairs of neighbouring origins. Time measured in rbp. For each triplet $\sum_i \mu_i = 0$ (up to sampling error) because of the normalisation of realised firing times to sum to zero. The obscuring probabilities of each origin are given in row 5 (standard deviation row 6), given as 0 when the MCMC output was 1e-3. Same holds for licensing probabilities (row 7 and 8) respectively. * indicates probabilities significantly different from 0 and 1 ($p < 0.05$ assuming normal distribution). The mean and standard deviations of the probabilities $\pi(t_{i+1} < t_i)$, $i = 1, 2, 3$ are computed for each neighbouring pair of origins based on a Gaussian model with mean $\mu_{i+1} - \mu_i$ and standard deviation $\sqrt{\sigma_i^2 + \sigma_{i+1}^2}$. For pair O_2O_3 the first value is inferred from the triplet $O_1O_2O_3$ and the second one from $O_2O_3O_4$. Posterior mean and standard deviations of the firing time parameters μ_i and σ_i for origin O_i. Mean and standard deviations of the probabilities $\pi(t_{i+1} < t_i)$, $i = 1, 2, 3$ that in a pair of consecutive origins the right one fires first. For $i = 2, 3$ the first value is inferred from the triplet $O_1O_2O_3$ and the second one from $O_2O_3O_4$. We also give the obscuring probabilities of O_1, O_3 and O_4.

	O_1	O_2	O_3	O_1O_2	O_2O_3	O_3O_1
mean μ	6957	-9804	2849	-16761	12654	-4107
sd μ	507	811	404	1285	1180	409
mean σ	8840	4507	4378	9948	6311	9897
sd σ	514	740	718	552	841	388
mean *obsc*	0.09*	0	0.01			
sd *obsc*	0.01*	0	0.01			
mean q	1	0.12*	1			
sd q	0	0.01*	0			
mean $\pi(t_{i+1} < t_i)$				0.95	0.03	0.66
sd $\pi(t_{i+1} < t_i)$				0.01	0.02	0.02

Table S2 **Inferred origin characteristics** ***ARS207.5-208.*** Posterior mean and standard deviations of the firing time parameters μ_i and σ_i for origin O_i, and their differences between pairs of neighbouring origins. Time measured in rbp. For each triplet $\sum_i \mu_i = 0$ (up to sampling error) because of the normalisation of realised firing times to sum to zero. The obscuring probabilities of each origin are given in row 5 (standard deviation row 6), given as 0 when the MCMC output was 1e-3. Same holds for licensing probabilities (row 7 and 8) respectively. * indicates probabilities significantly different from 0 and 1 ($p < 0.05$ assuming normal distribution). The mean and standard deviations of the probabilities $\pi(t_{i+1} < t_i)$, $i = 1, 2, 3$ are computed for each neighbouring pair of origins based on a Gaussian model with mean $\mu_{i+1} - \mu_i$ and standard deviation $\sqrt{\sigma_i^2 + \sigma_{i+1}^2}$.

	$O_1O_2O_3$	$O_2O_3O_4$	$O_3O_4O_5$	$O_4O_5O_6$	$O_5O_6O_7$	$O_6O_7O_8$	$O_7O_8O_9$	$O_8O_9O_{10}$
q_1	1	-	-	-	-	-	-	-
q_2	0.64	1	-	-	-	-	-	-
q_3	1	0.80	0.71	-	-	-		
q_4	-	1	1	1	-	-	-	-
q_5	-	-	0.86	0.83	1	-	-	-
q_6	-	-	-	1	0.82	1	-	-
q_7	-	-	-	-	1	0.93	0.94	-
q_8	-	-	-	-	-	1	1	0.98
q_9	-	-	-	-	-	-	1	1
q_{10}	-	-	-	-	-	-	-	1
obsc1	0.11	-	-	-	-	-	-	-
obsc21	0.02	-	-	-	-	-	-	-
obsc23	0.14	0.46	-	-	-	-	-	-
obsc32	0	0	-	-	-	-	-	-
obsc34	-	0.05	0.01	-	-	-	-	-
obsc43	-	0.01	0	-	-	-	-	-
obsc45	-	-	0	0	-	-	-	-
obsc54	-	-	0.63	0.55	-	-	-	-
obsc56	-	-	-	0	0	-	-	-
obsc65	-	-	-	0	0	-	-	-
obsc67	-	-	-	-	0.1	0.21	-	-
obsc76	-	-	-	-	0	0	-	-
obsc78	-	-	-	-	-	0	0	-
obsc87	-	-	-	-	-	0.37	0.32	-
obsc89	-	-	-	-	-	-	0	0
obsc98	-	-	-	-	-	-	0.02	0.03
obsc910	-	-	-	-	-	-	-	0.08
obsc10	-	-	-	-	-	-	-	0.01

Table S3 Annotation for the mean obscuring and licensing probabilities for each of the 8 consecutive triplets (*ARS1001*-*ARS1011*) for figure S26. Notation as in table 2 of the Main text.

Origins	Licensing q estimate	Efficiency estimate	Licensing 1	Efficiency 1	Efficiency 2	Efficiency 3a	Efficiency 3b
ARS207.5	0.9998	0.9135	0.918	0.585	0.598	0.864	0.944
ARS207.8	0.1211	0.1211	0.869	0.313	0.0	NA	NA
ARS208	0.9998	0.9944	0.793	0.695	0.513	1.0	0.76
ARS717	0.7457	0.7457	0.83	0.773	0.53	0.74	0.816
ARS718	0.9989,0.9984	0.9946,0.9984	0.913	0.819	0.625	0.872	0.972
ARS719	0.9851,0.9978	0.9851,0.9978	0.809	0.771	0.627	1.0	1.0
ARS720	0.9272	0.9272	0.557	0.484	0.588	0.957	1.0
ARS813	0.9913	0.8523	0.98	0.834	0.6	0.742	0.794
ARS815	0.9700,0.9988	0.9689,0.9983	0.953	0.908	0.652	0.774	0.865
ARS816	0.9921,0.9031	0.8266,0.7562	0.779	0.699	0.364	0.641	0.751
ARS818	0.9992	0.9133	0.53	0.462	0.409	0.706	0.582
ARS1001	0.9996	0.8886	0.275	0.215	NA	NA	NA
ARS1004	0.6374,0.9992	0.4846,0.5352	0.961	0.337	0.173	0.273	0.327
ARS1005	0.9996, 0.7973,0.7052	0.9996,0.7496,0.6965	0.94	0.834	0.445	0.581	0.674
ARS1006	0.9986,0.9995,0.9982	0.9931,0.9991,0.998	0.289	0.068	0.608	0.746	0.785
ARS1007	0.8644,0.8259,0.9994	0.2378,0.2753,0.9974	0.732	0.662	0.152	NA	NA
ARS1007.5	0.9997,0.8152,0.9968	0.9983,0.7182,0.7855	0.806	0.679	0.434	0.577	0.0.722
ARS1008	0.9981,0.9289,0.9426	0.9954, 0.9273,0.9421	0.963	0.845	0.599	0.859	0.963
ARS1009	0.9970,0.9964, 0.9794	0.6285,0.6812, 0.9793	0.755	0.449	0.331	0.342	0.509
ARS1010	0.9995,0.9950,0.9997	0.9757,0.8868,0.9628	0.813	0.678	0.603	0.676	0.908
ARS1011	0.9924,0.2601,0.9984	0.9857,0.2585, 0.4801	0.554	0.494	0.199	0.281	0.158
ARS1013	0.9998,0.8679,0.8648	0.9997,0.867,0.8646	0.82	0.79	0.562	0.838	0.886
ARS1014	0.9968,0.9992,0.9982	0.9968,0.9992,0.9982	0.817	0.769	0.556	0.923	1.0
ARS1015	0.8989,0.9212,0.9909	0.8989,0.9212,0.9909	0.4	0.271	0.59	0.927	0.953
ARS1018	0.9971,0.9495,0.9552	0.9971,0.9495,0.9552	0.986	0.936	0.606	0.64	0.751
ARS1019	0.9862,0.9985	0.9862,0.9985	0.984	0.889	0.624	0.685	0.725
ARS1021	0.9026	0.9026	0.983	0.836	0.645	0.74	0.732
Correlation	-	-	0.07	0.31	0.93	0.81	0.85

Table S4 **Comparison table for origin efficiencies and licensing probabilities inferred using our MCMC algorithm and the values estimated in [Hawkins et al., 2013] and [McGuffee et al., 2013]**. Multiple values correspond to the cases when inference was performed using more than one triple of origins. Licensing 1 and Efficiency 1 columns correspond to the values given in [Hawkins et al., 2013] obtained by analysing the time course data and Efficiency 2 are the ones given in [Hawkins et al., 2013] by analysing Okazaki fragment data from [Smith and Whitehouse, 2012], Efficiency 3a,b are the origin efficiensies given in [McGuffee et al., 2013] for WT A and WT B respectively. Last row is the correlation coefficients between the inferred licensing probabilities and origin efficiencies for both datasets.

	O_1	O_2	O_3	O_4	O_1O_2	O_2O_3	O_3O_4
mean μ	-55520	36070,27778	19467,22600	-50423	-91590	-16603,-5179	73023
sd μ	2279	1558, 2432	2431,1347	2300	3286	2950, 3064	3018
mean σ	8767	70624, 118450	137560, 8642	103790	71298	154710,118840	104240
sd σ	4400	4077, 5670	9131, 4070	10512	4141	8680,5561	10395
mean *obsc*	0.0	0.0	0.0,0.01	0.0			
sd *obsc*	0.0	0.0	0.0,0.0	0.0			
mean q	0.92*	1.0,0.97	0.99,1.0	0.81*			
sd q	0.01*	0.0,0.1	0.01,0.0	0.01*			
mean $\pi(t_{i+1} < t_i)$					0.1	0.54,0.53	0.76
sd $\pi(t_{i+1} < t_i)$					0.01	0.01,0.01	0.02

Table S5 **Inferred origin characteristics, human chromosome 2 (98.25 - 99.3 Mb).** Posterior mean and standard deviations of the firing time parameters μ_i and σ_i for origin O_i, and their differences between pairs of neighbouring origins. Time measured in rbp. For each triplet $\sum_i \mu_i = 0$ (up to sampling error) because of the normalisation of realised firing times to sum to zero. The obscuring probabilities of each origin are given in row 5 (standard deviation row 6), given as 0 when the MCMC output was 1e-3. Same holds for licensing probabilities (row 7 and 8) respectively. * indicates probabilities significantly different from 0 and 1 ($p < 0.05$ assuming normal distribution). The mean and standard deviations of the probabilities $\pi(t_{i+1} < t_i)$, $i = 1, 2, 3$ are computed for each neighbouring pair of origins based on a Gaussian model with mean $\mu_{i+1} - \mu_i$ and standard deviation $\sqrt{\sigma_i^2 + \sigma_{i+1}^2}$. For pair O_2O_3 the first value is inferred from the triplet $O_1O_2O_3$ and the second one from $O_2O_3O_4$. Posterior mean and standard deviations of the firing time parameters μ_i and σ_i for origin O_i. Mean and standard deviations of the probabilities $\pi(t_{i+1} < t_i)$, $i = 1, 2, 3$ that in a pair of consecutive origins the right one fires first. For $i = 2, 3$ the first value is inferred from the triplet $O_1O_2O_3$ and the second one from $O_2O_3O_4$. We also give the obscuring probabilities of O_1, O_3 and O_4.
