# Supplementary material for: Bayesian inference of origin firing time distributions, origin interference and licencing probabilities from Next Generation Sequencing data
Source: Nucleic Acids Res. 2019 Feb 14;47(5):2229–43. doi: 10.1093/nar/gkz094 (PMC6412128; doi:10.1093/nar/gkz094)
Supplement: Supplementary Data [file gkz094_supplemental_files.zip › BazarovaSuppFigsS18-S33.pdf]

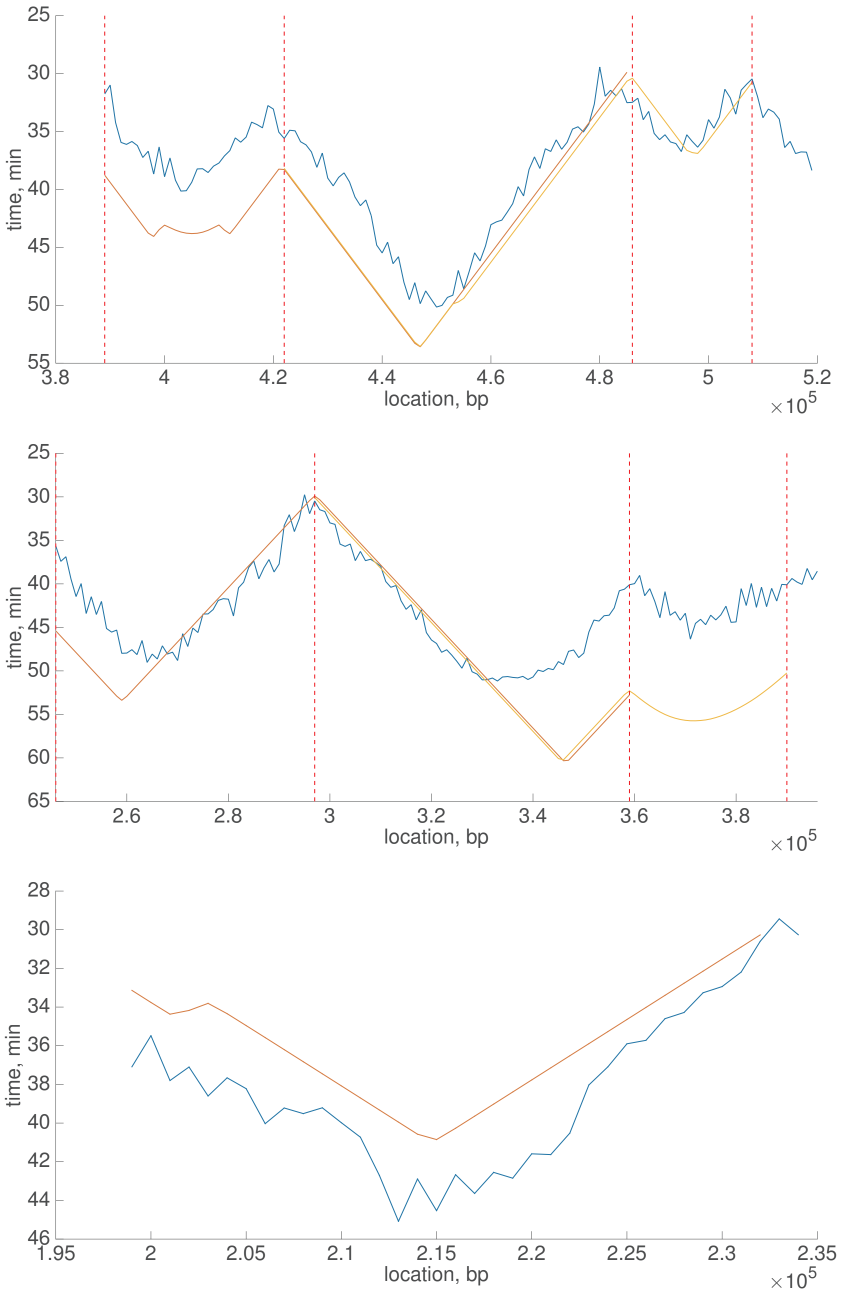


Figure S18 **Median replication times of** ***ARS717-720*****,** ***ARS813-818*****,** ***ARS207.5-208*** **of main text.** Median replication times $T_{rep}$ given in [Müller et al., 2014] (blue) and the ones derived from the inferred parameters (orange for the left triplet, yellow for the right one). Regions *ARS717-720* (upper panel) *ARS813-818* (middle panel), *ARS207.5-208* (lower panel). Origins shown by dashed vertical lines.

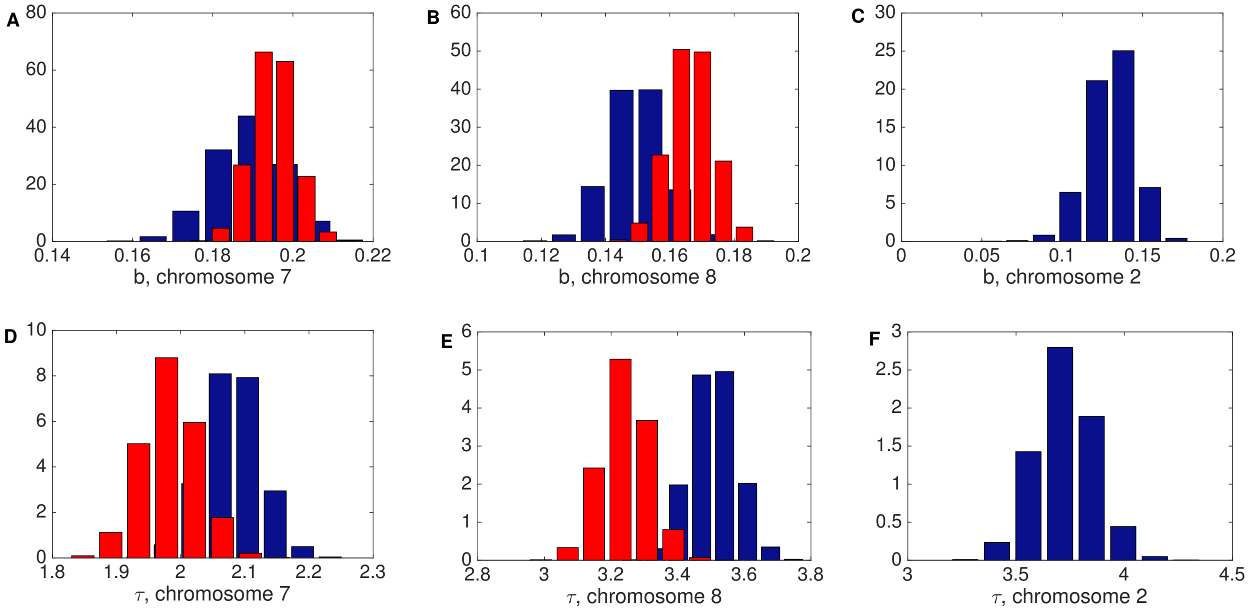


Figure S19 **Distributions of b and $\tau$ parameters. A, D:** chromosome 7 from section Lack of origin obscuring in strong origin triplets: *ARS717-20*, **B, E:** chromosome 8 from section Triplets with higher obscuring rates: *ARS813-18*, **C, F:** chromosome 2 from section Early, poorly licenced origin: *ARS207.5*, *ARS207.8*, *ARS208*. In case of two colours blue corresponds to the values inferred from the left triplet and the red to the values from the right triplet

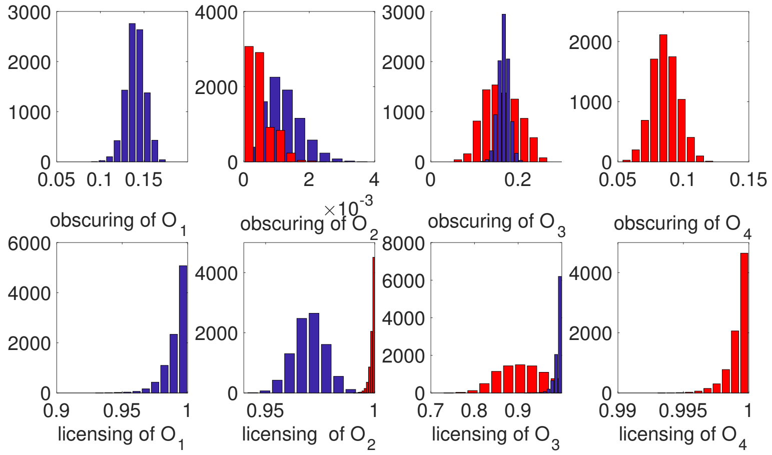


Figure S20 *ARS813-818* **obscuring (upper panel) and licensing (lower panel) probabilities**. Red are the histograms inferred from the right triplet, blue are the ones inferred from the left triplet. For corresponding reconstructed profiles and distributions of firing time differences see Fig. 5 of the Main Text

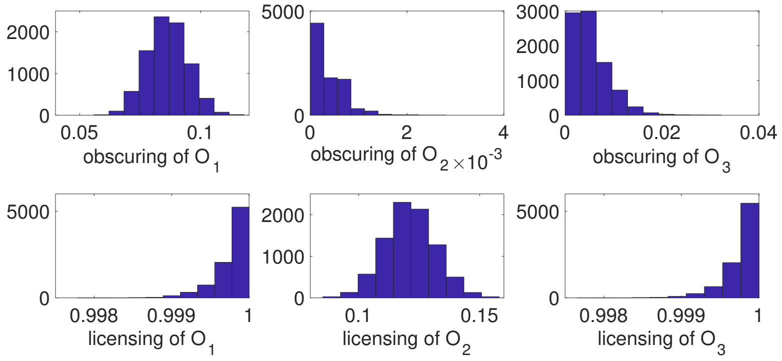


Figure S21 *ARS207.5-207.8* **obscuring (upper panel) and licensing (lower panel) probabilities.** For profile reconstruction and the inferred distributions of firing time differences see Fig. 6 of the Main Text

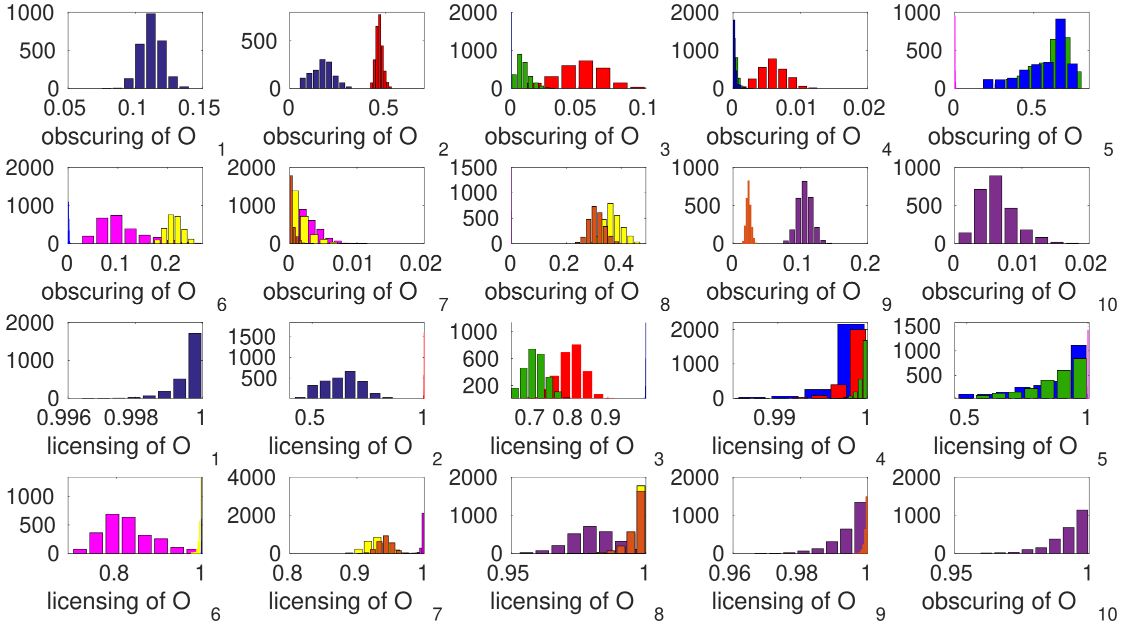


Figure S22 *ARS1001-1011* **obscuring (A, B) and licensing (C, D) probabilities.** Histograms inferred from $O_1O_2O_3$ (dark blue), $O_2O_3O_4$ (red), $O_3O_4O_4$ (green), $O_4O_5O_6$ (blue), $O_5O_6O_7$ (pink), $O_6O_7O_8$ (yellow), $O_7O_8O_9$ (orange), $O_8O_9O_{10}$ (purple). For profile reconstruction and the inferred distributions of firing time differences see Fig. S26
.

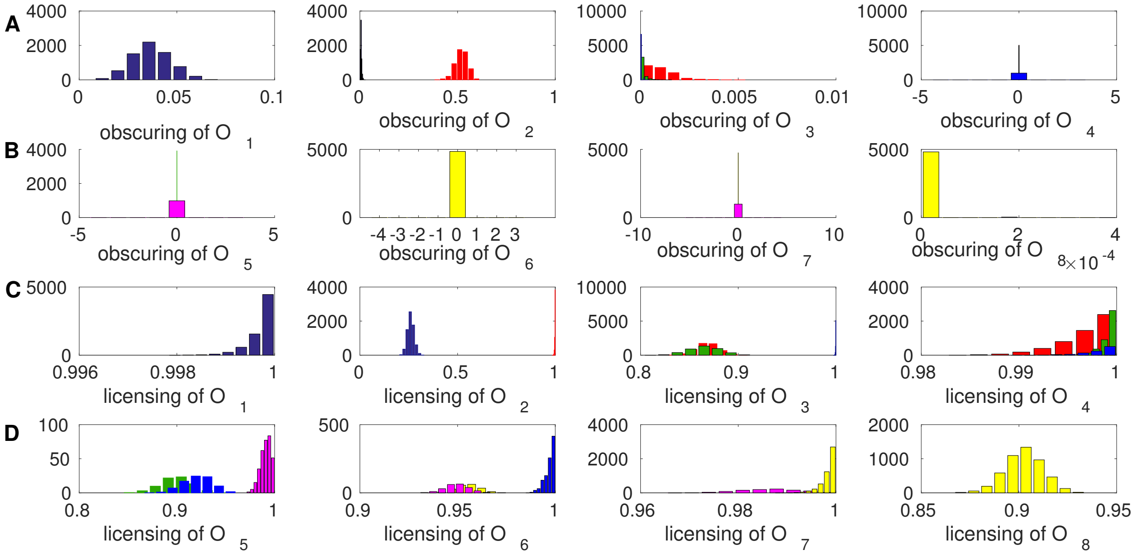


Figure S23 *ARS1010-1021* **obscuring (A, B) and licensing (C, D) probabilities.** Histograms inferred from $O_1O_2O_3$ (dark blue), $O_2O_3O_4$ (red), $O_3O_4O_4$ (green), $O_4O_5O_6$ (blue), $O_5O_6O_7$ (pink), $O_6O_7O_8$ (yellow). For profile reconstruction and the inferred distributions of firing time differences see Fig. 7 of the Main Text

.

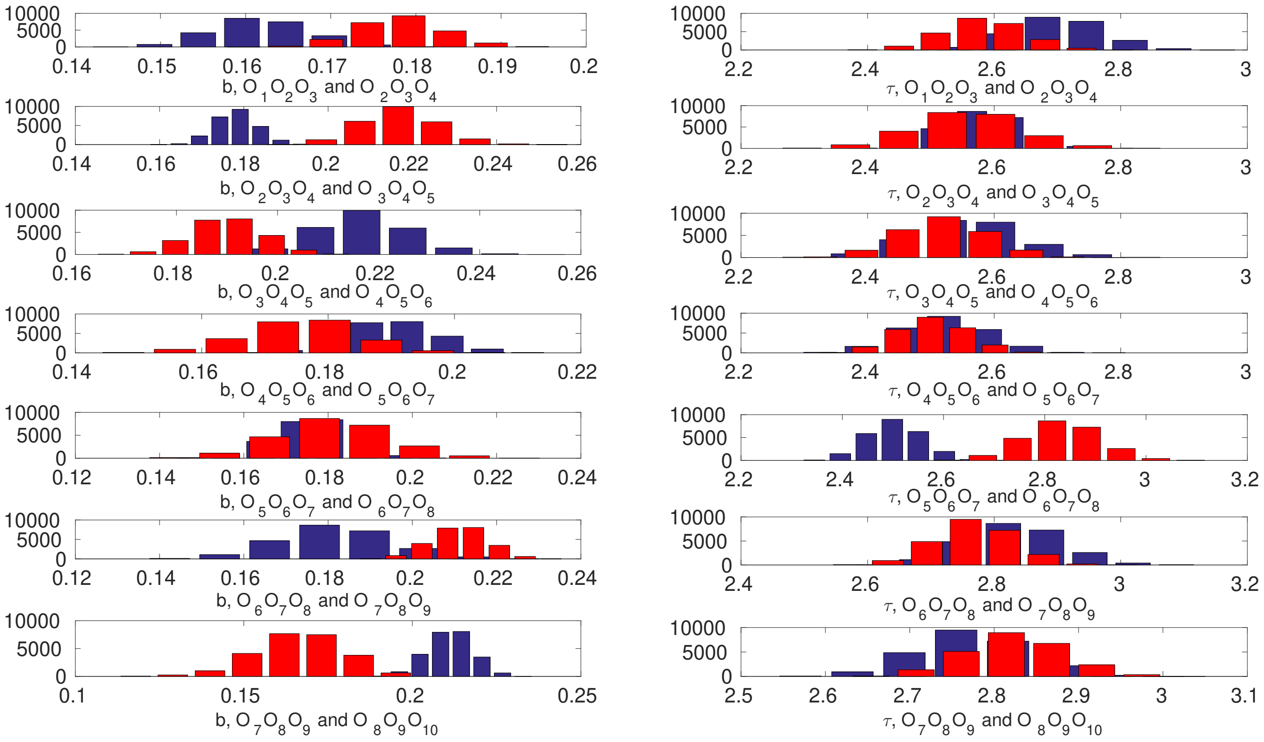


Figure S24 **Distributions of b and $\tau$ parameters, chromosome 10,** *ARS1001-ARS1011*. **Left panels:** parameter b **Right panels:** parameter $\tau$ . Blue corresponds to the values inferred from the left triplet and the red to the values from the right triplet. Analysis given for the region on chromosome 10 from section Analysis of chromosome 10

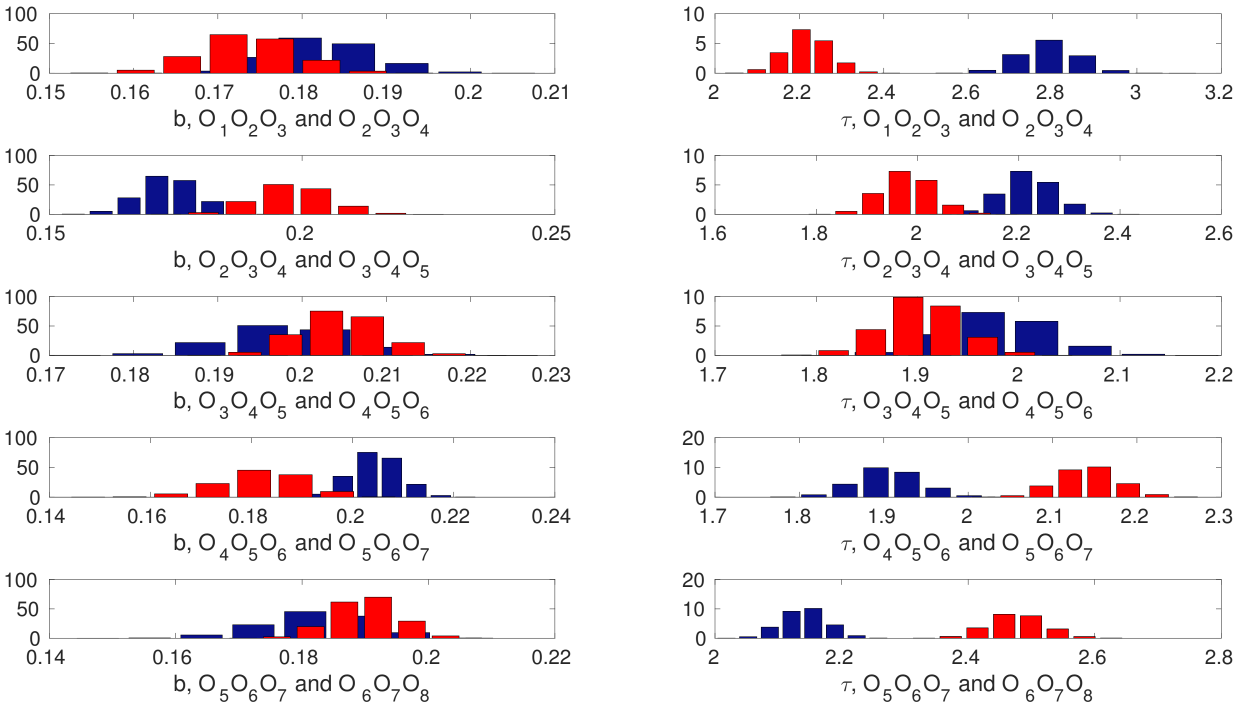


Figure S25 **Distributions of b and $\tau$ parameters, chromosome 10,** ***ARS1010-ARS1021*****. Left panels:** parameter b **Right panels:** parameter $\tau$ . Blue corresponds to the values inferred from the left triplet and the red to the values from the right triplet. Analysis given for the region on chromosome 10 from section Analysis of chromosome 10

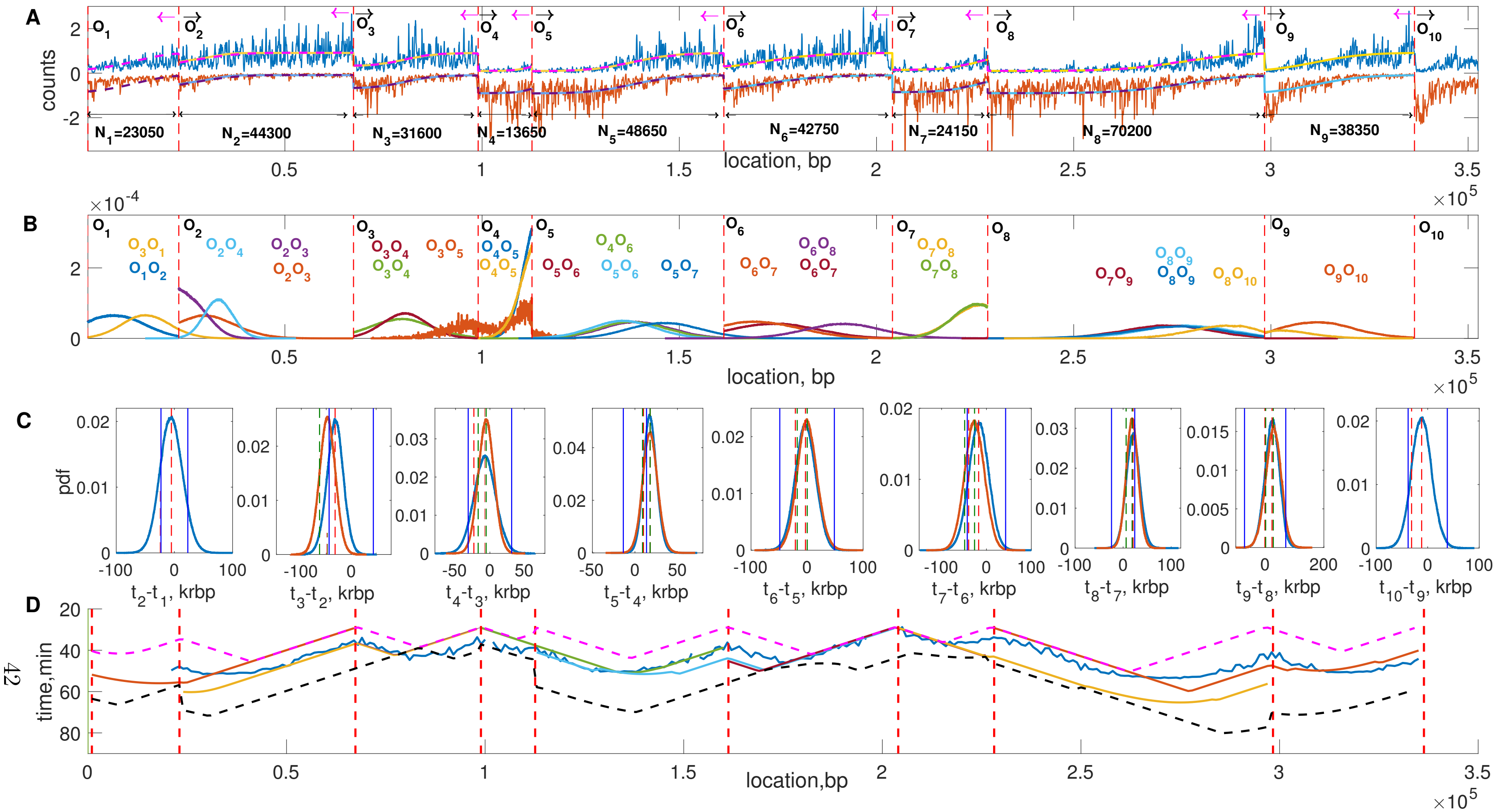


Figure S26 **Chromosome 10 analysis: consecutive origins** ***ARS1001-11*****.** Analysis of the region between *ARS1001* and *ARS1011* on chromosome 10 obtained by applying the algorithm separately on eight consecutive triplets with the 10 origins *ARS1001*, *ARS1004-1011*,**A**: Data on the forward (blue) and reverse (red) strands and reconstructed fragment profiles (dashed magenta and purple for the left triplets, solid yellow and blue for right ones). Dashed vertical lines indicate the locations of the eight origins $O_1 - O_{10}$. Notation as in figure 7 of the Main Text. **B**: Probability density distributions of the realised collision points between neighbouring origins as indicated, and non neighbours. The text colour of the annotation corresponds to the distribution of the same colour **C**:Inferred distributions of the firing time differences between neighbouring origins conditioned on origin being licensed. Notation as in Fig. 3 of the Main Text. **D**: Median replication times $T_{rep}$ across chromosome 10. Notation as in figure 8 of the Main Text. For licensing and obscuring histograms see Fig. S22. Inference based on a single MCMC run with burn-in 100000, and 100000 samples post burn-in. See section S1.1 for MCMC information section S1.1.

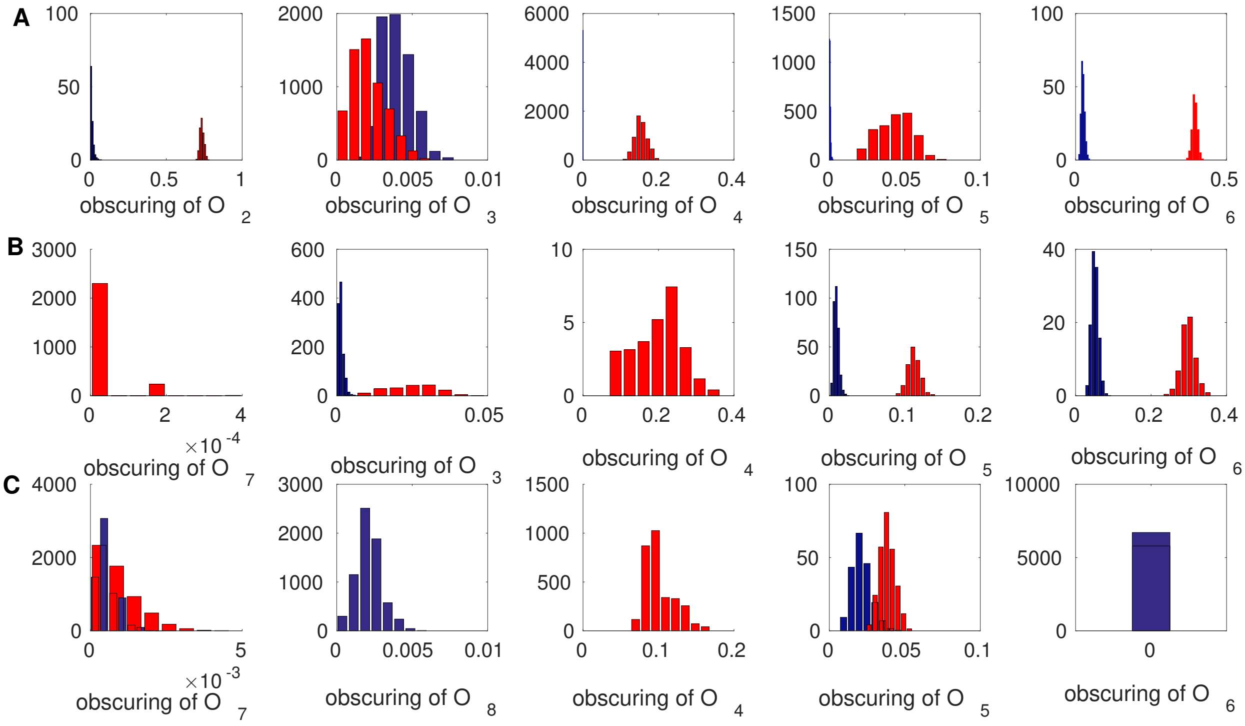


Figure S27 **Obscuring data for WT (blue) and *rat1-1* inactivation (red).** Where only one colour is represented means that the other one corresponds to zero obscuring. In cases of multiple plots for an origin the upper panel corresponds to the values inferred from the left triplet (A), middle one - from the middle triplet (B), lower one - from the right triplet (C).

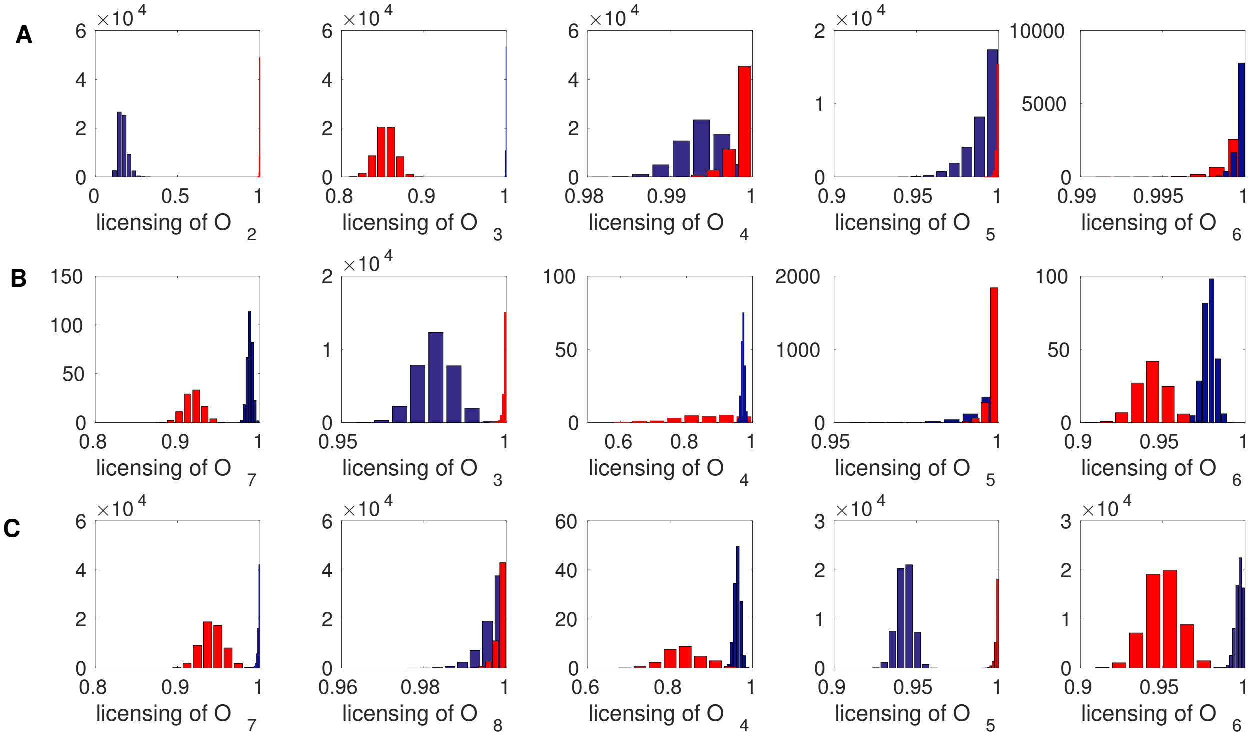


Figure S28 **Licensing data for WT (blue) and *rat1-1* inactivation (red).** In cases of multiple plots for an origin the lower panel corresponds to the values inferred from the left triplet (A), middle one - from the middle triplet (B), upper one - from the right triplet (C).

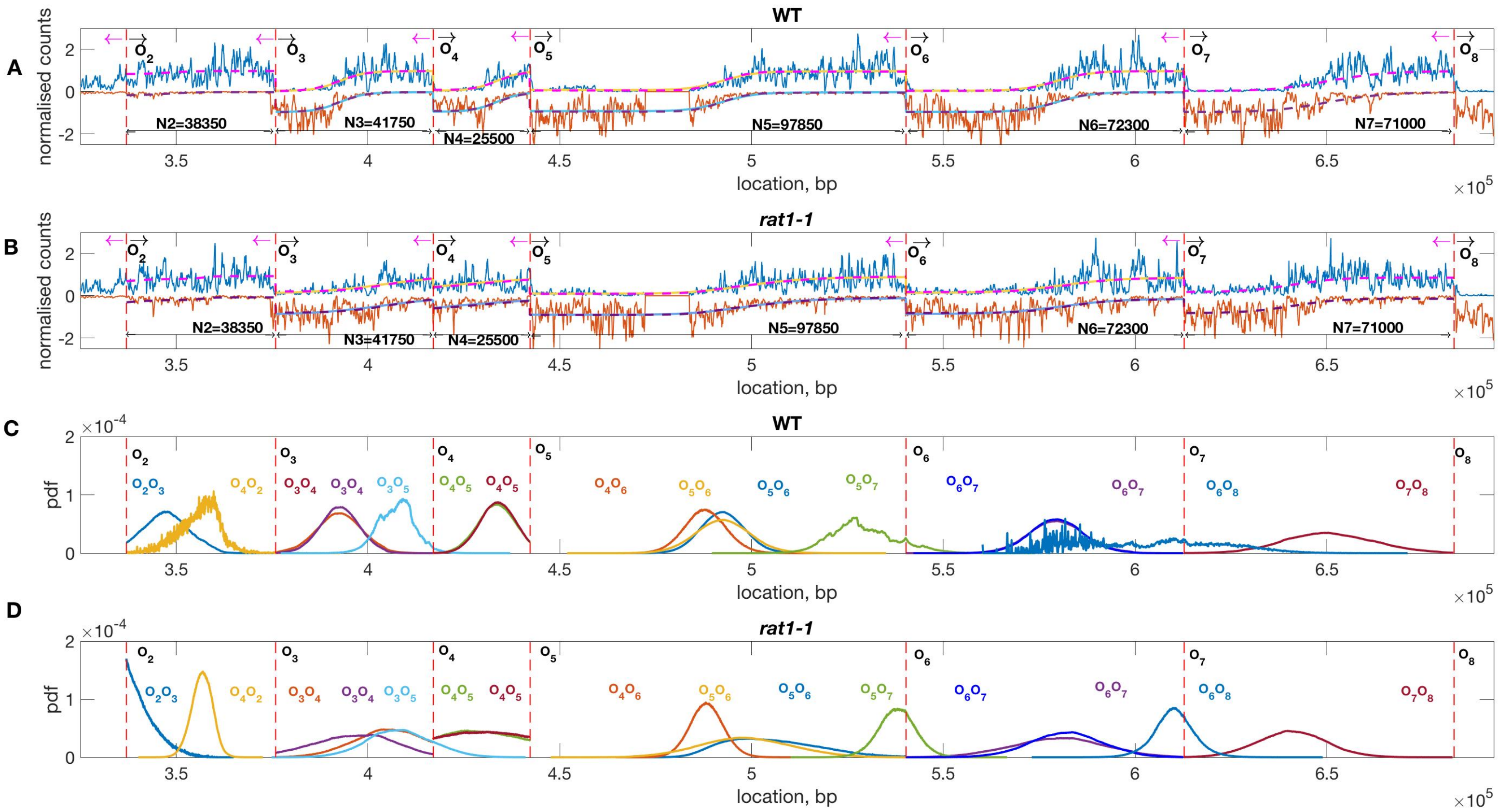


Figure S29 **Comparison of the inference for WT (A,C) and *rat1-1* inactivation (B,D) for chromosome 10 (*ARS1011-1021*).** Profile reconstructions and collision points distributions for WT and *RAT1* inactivation data. Notation as in Figure 7A,B. Note origin labels $O_i$ shifted by 1 relative to Figure 7.

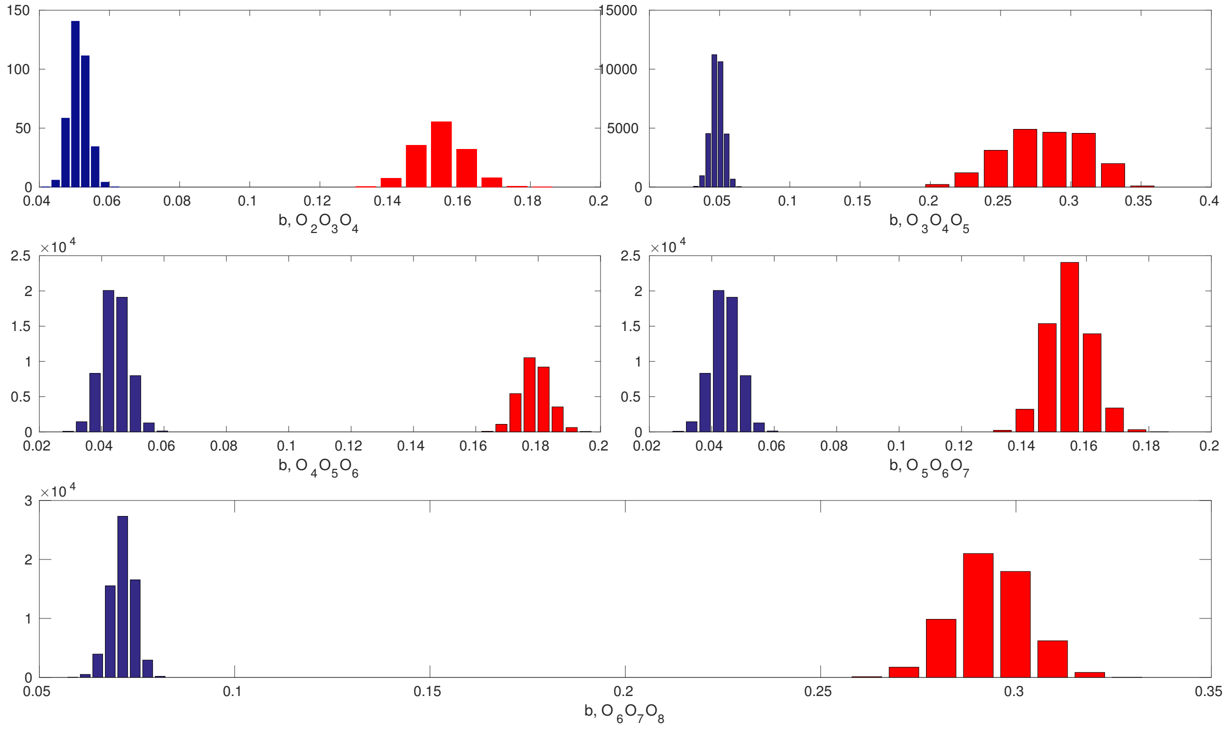


Figure S30 **Parameter b inference for WT (blue) and *rat1-1* inactivation (red).** Each of the panels corresponds to one of the 5 analysed triplets of chromosome 10 from section Results: *rat1-1* inactivation reduces licensing and increases obscuring of the Main Text.

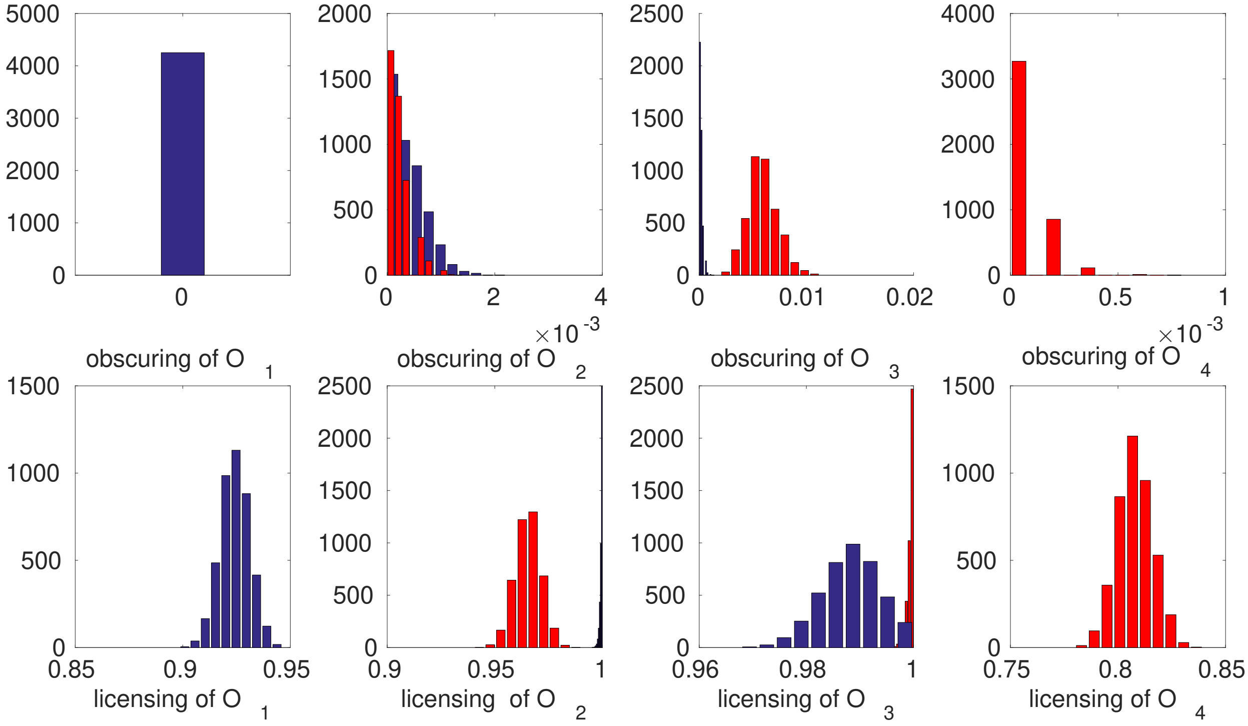


Figure S31 **OK-seq data, chromosome 2 (98.25 - 99.3 Mb), obscuring (upper panel) and licensing (lower panel) probabilities**. Red are the histograms inferred from the right triplet, blue are the ones inferred from the left triplet. For corresponding reconstructed profiles and distributions of firing time differences see Fig. 11 of the Main Text

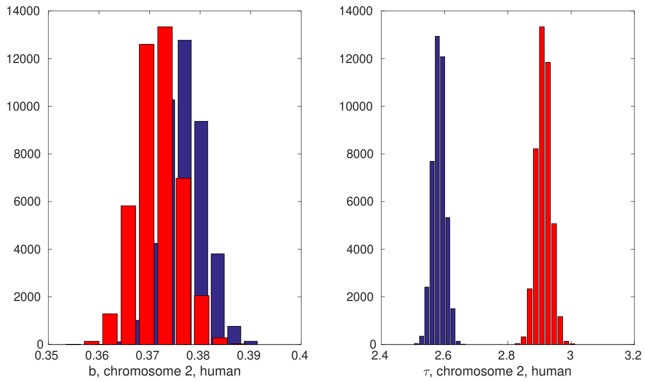


Figure S32 **Distributions of b and $\tau$ parameters for OK-seq data.** Chromosome 2 (98.25 - 99.3 Mb), blue corresponds to the values inferred from the left triplet and the red to the values from the right triplet. Analysis given for the region on chromosome 2 from section A region with minimal origin obscuring and strong origins in Human cells

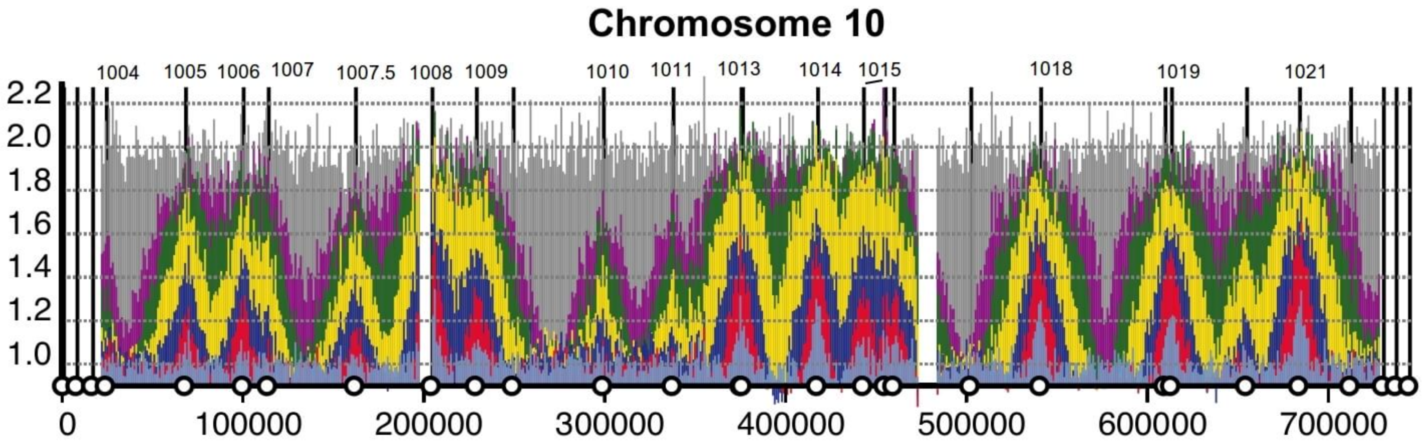


Figure S33 **Time course data for chromosome 10**. Reproduced from [Müller et al., 2014] annotated with origins discusseded in this study. (vertical lines).
